# Synthesis of 1-(2-Hydroxyphenyl)- and (3,5-Dichloro-2-hydroxyphenyl)-5-oxopyrrolidine-3-carboxylic Acid Derivatives as Promising Scaffolds for the Development of Novel Antimicrobial and Anticancer Agents

**DOI:** 10.3390/ijms24097966

**Published:** 2023-04-27

**Authors:** Monika Bertašiūtė, Povilas Kavaliauskas, Rita Vaickelionienė, Birutė Grybaitė, Vidmantas Petraitis, Rūta Petraitienė, Ethan Naing, Andrew Garcia, Jūratė Šiugždaitė, Raimundas Lelešius, Vytautas Mickevičius

**Affiliations:** 1 Department of Organic Chemistry, Kaunas University of Technology, Radvilėnų Rd. 19, 50254 Kaunas, Lithuania; monika.bertasiute@ktu.lt (M.B.); pok4001@med.cornell.edu (P.K.); birute.grybaite@ktu.lt (B.G.); 2 Transplantation-Oncology Infectious Diseases Program, Division of Infectious Diseases, Department of Medicine, Weill Cornell Medicine of Cornell University, 1300 York Ave., New York, NY 10065, USA; vip2007@med.cornell.edu (V.P.); rop2016@med.cornell.edu (R.P.); etn2001@med.cornell.edu (E.N.); abg2014@med.cornell.edu (A.G.); 3 Institute for Genome Sciences, School of Medicine, University of Maryland Baltimore School of Medicine, 655 W. Baltimore Street, Baltimore, MD 21201, USA; 4 Institute of Infectious Diseases and Pathogenic Microbiology, Birštono Str. 38A, 59116 Prienai, Lithuania; 5 Biological Research Center, Lithuanian University of Health Sciences, Tilžės St. 18, 47181 Kaunas, Lithuania; 6 Department of Pathobiology, Lithuanian University of Health Sciences, Tilžės St. 18, 47181 Kaunas, Lithuania; siujur@gmail.com (J.Š.); raimundas.lelesius@lsmu.lt (R.L.)

**Keywords:** pyrrolidinone, hydrazone, azole, Gram-positive bacteria, antimicrobial activity, anticancer activity

## Abstract

Increasing antimicrobial resistance among Gram-positive pathogens and pathogenic fungi remains one of the major public healthcare threats. Therefore, novel antimicrobial candidates and scaffolds are critically needed to overcome resistance in Gram-positive pathogens and drug-resistant fungal pathogens. In this study, we explored 1-(2-hydroxyphenyl)-5-oxopyrrolidine-3-carboxylic acid and its 3,5-dichloro-2-hydroxyphenyl analogue for their in vitro antimicrobial activity against multidrug-resistant pathogens. The compounds showed structure-dependent antimicrobial activity against Gram-positive pathogens (*S. aureus*, *E. faecalis*, *C. difficile*). Compounds **14** and **24b** showed promising activity against vancomycin-intermediate *S. aureus* strains, and favorable cytotoxic profiles in HSAEC-1 cells, making them attractive scaffolds for further development. 5-Fluorobenzimidazole, having a 3,5-dichloro-2-hydroxyphenyl substituent, was found to be four-fold, and hydrazone, with a thien-2-yl fragment, was two-fold stronger than clindamycin against methicillin resistant *S. aureus* TCH 1516. Moreover, hydrazone, bearing a 5-nitrothien-2-yl moiety, showed promising activity against three tested multidrug-resistant *C. auris* isolates representing major genetic lineages (MIC 16 µg/mL) and azole-resistant *A. fumigatus* strains harboring TR34/L98H mutations in the CYP51A gene. The anticancer activity characterization demonstrated that the 5-fluorobenzimidazole derivative with a 3,5-dichloro-2-hydroxyphenyl substituent showed the highest anticancer activity in an A549 human pulmonary cancer cell culture model. Collectively these results demonstrate that 1-(2-hydroxyphenyl)-5-oxopyrrolidine-3-carboxylic acid derivatives could be further explored for the development of novel candidates targeting Gram-positive pathogens and drug-resistant fungi.

## 1. Introduction

Infections caused by multidrug-resistant (MDR) Gram-positive bacteria and drug-resistant (DR) fungi remain a major healthcare problem, with the majority of the cases occurring in critically ill individuals or patients undergoing chemotherapy or solid organ transplantation. Among MDR Gram-positive pathogens, methicillin-resistant *Staphylococcus aureus* (MRSA), vancomycin-resistant enterococci (VRE), and *Clostridioides difficile* (*C. difficile*) are responsible for the majority of cases. Moreover, bloodstream infections caused by MRSA and other Gram-positive pathogens often have a poor prognosis and result in the death of the patients. Therefore, it is crucial to develop novel compounds targeting Gram-positive pathogens [1].

Vancomycin and other structurally related glycopeptides are the last resort antimicrobials used to treat severe infections caused by Gram-positive pathogens. The resistance to vancomycin was first reported in 1986, and nowadays, resistance is often observed in clinical settings [2,3]. The molecular determinants encoding the resistance to vancomycin are encoded by a transposon located in the plasmids, thus permitting the lateral spread of numerous Gram-positive pathogens. Vancomycin resistance is now being observed in various aerobic and anaerobic Gram-positive pathogens, such as *S. aureus*, *Enterococcus* spp., and *C. difficile* [4,5]. The rapid spread of the resistance determinants among Gram-positive pathogens, as well as the rising number of cases associated with vancomycin-intermediate or vancomycin-resistant strains, urges the development of novel therapies to restore susceptibility to last-line drugs or provide new candidates selectively targeting drug-resistant pathogens.

Pathogenic fungi are associated with approximately 1.5 million deaths and 1.7 billion superficial infections every year, resulting in a massive economic burden on healthcare [6,7]. Yeast belonging to *Candida* species (predominantly *C. albicans*), as well as molds belonging to *Aspergillus* (predominantly *A. fumigatus*), are responsible for the majority of invasive fungal infection (IFI) cases worldwide. Various azole antifungal drugs (fluconazole, voriconazole, itraconazole, etc.) are the first line of drugs to treat IFIs caused by *Candida* spp. and *A. fumigatus.* Moreover, increasingly drug-resistant *Candida* species, such as *C. auris* harboring numerous resistance determinants, are increasingly being isolated from critically ill patients, resulting in a shortage of available treatment options and increased mortality. Furthermore, the emergence of azole-resistant *A. fumigatus* strains, harboring an azole-resistance (AR) phenotype associated TR34/L98H mutations in the *CYP51A* gene [8,9], makes these infections caused by AR *A. fumigatus* extremely lethal and requires various compassionate care or investigational therapeutic options. Therefore, new antifungal drug candidates are critically needed to overcome antifungal resistance in highly drug-resistant *Candida* species, as well as AR *A. fumigatus* with TR34/L98H mutations in the *CYP51A* gene. In addition to that, since fungi are eucaryotic organisms, many targets located in fungal cells overlap with host cellular targets. Therefore, compounds with antifungal activity could be also further explored for their anticancer properties.

Azoles are a diverse and important class of five-membered, nitrogen-containing, heterocyclic organic molecules that may possess other non-carbon atoms, such as oxygen or sulfur, thus making them an extremely structurally versatile class of molecules.

Diversely functionalized azole analogues are considered to be one of the most important frameworks for the development of numerous pharmacologically active compounds with antifungal, antibacterial, antidiabetic, and anticancer activities [10,11,12,13,14,15,16,17,18]. Interestingly, several studies have shown that azole derivatives containing naphthalene show selective and Gram-positive-bacteria-directed antimicrobial activity [19]. Moreover, the conjugation of metal nanoparticles with azole antimicrobial derivatives greatly enhances antibacterial activity against pan-susceptible and drug-resistant *S. aureus*, suggesting that azole derivatives could be explored as Gram-positive-bacteria-directed antimicrobials [20]. The chemical versatility of azoles and the ability to incorporate numerous substitutions in the core structure makes azoles an attractive scaffold for the development of novel dual active antimicrobial candidates targeting Gram-positive bacterial and fungal pathogens.

The identification of potent pharmacophores is paramount for the development of novel broad-spectrum antimicrobial candidates. As an example, benzimidazole core incorporation in target structures greatly enhances the pharmacological properties of various molecules due to the formation of fused ring benzimidazole compounds.

Compounds bearing various fused-ring benzimidazole moieties demonstrate promising antimicrobial activities against numerous pathogens [21,22]. Benzimidazole is an electron-rich pharmacophore that can easily accept or donate protons and easily form a variety of weak interactions leading benzimidazole pharmacophores to bind different cellular targets. With that in mind, benzimidazole core-containing compounds have been previously reported to show anticancer activity in different cancer cell culture models [22,23,24]. Therefore, possibly, compounds containing azole and benzimidazole moieties could show potential antifungal and antibacterial activity targeting drug-resistant fungal and bacterial pathogens [25,26,27,28,29,30].

The increasing antimicrobial resistance among Gram-positive bacterial pathogens to last-line antimicrobials urges the identification of novel candidates for further pre-clinical antimicrobial drug development. With growing numbers of cancer and chemotherapy-associated immunosuppression cases, drug-resistant fungal species can often co-infect individuals suffering from infections caused by Gram-positive pathogens. Therefore, the development of novel antimicrobial candidates, targeting multidrug-resistant Gram-positive pathogens and drug-resistant fungi, is critically needed. Our previous studies [31,32] on the development of novel compounds targeting multidrug-resistant pathogens have been reasonably successful in finding effective antimicrobial agents and showed higher bactericidal properties than ampicillin; therefore, we have continued the studies in this field. As the results demonstrate that 5-oxopyrolidine derivatives are attractive cores for the further development of potential candidates targeting multidrug-resistant Gram-positive pathogens and drug-resistant fungi with genetically defined resistance mechanisms, herein, we report the synthesis and bioevaluation of compounds having *N*-(2-hydroxyphenyl)- and *N*-(3,5-dichloro-2-hydroxyphenyl)-5-oxopyrrolidin-3-yl cores, as well their decyclization products, *γ*-amino acid derivatives. Their antimicrobial properties were focused on activity against *Staphylococcus aureus*, *Acinetobacter baumannii*, *Klebsiella pneumoniae*, *Pseudomonas aeruginosa*, *Clostridioides difficile*, *Candida auris*, and *Aspergillus fumigatus* strains. In addition to that, we characterized the *in vitro* cytotoxic and anticancer properties of novel compounds using the A549 human lung cell culture model. 

## 2. Results and Discussion

### 2.1. Chemistry

**Scheme 1:**
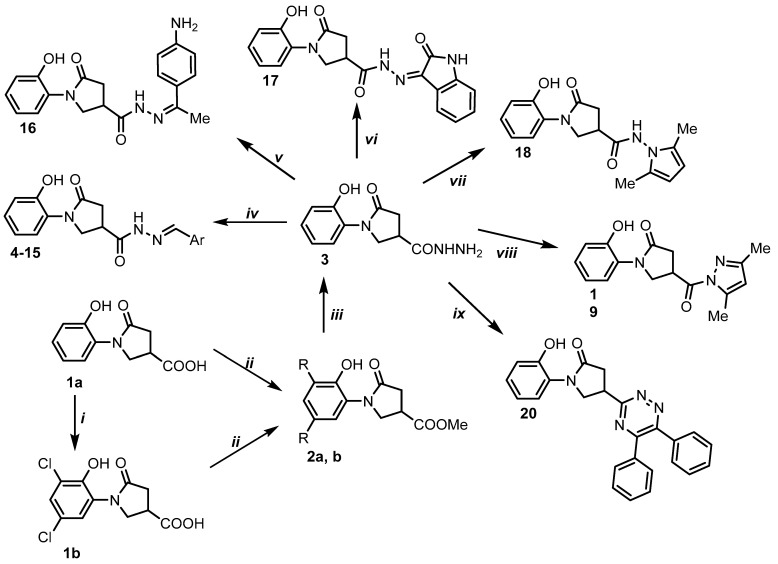
Synthesis of compounds **1**–**20. 2a** R = H; **2b** R = Cl; **4** Ar = C_6_H_5_, **5** Ar = 4-ClC_6_H_4_, **6** Ar = 4-BrC_6_H_4_, **7** Ar = 4-O_2_NC_6_H_4_, **8** Ar = 4-Me_2_NC_6_H_4_, **9** Ar = 4-MeOC_6_H_4_, **10** Ar = 2,4-di(MeO)C_6_H_3_, **11** Ar = 2,3,4-tri(MeO)C_6_H_2_, **12** Ar = 3,4,5-tri(MeO)C_6_H_2_, **13** Ar = 1-naphthyl, **14** Ar = thien-2-yl, **15** Ar = 5-nitrothien-2-yl. *Reagents and conditions:* ***i*** 6M HCl, H_2_O, H_2_O_2_, 25 °C, 1 h; ***ii*** MeOH, H_2_SO_4_, Δ, 2 h; 5% Na_2_CO_3_; ***iii*** N_2_H_4_·H_2_O, 2-PrOH, Δ, 2.5 h; ***iv*** ArCHO or CarbCHO, 2-PrOH, Δ, 2 h (for **4**–**13**, **15**) or 40 min (for **14**) h; ***v*** 4′-aminoacetophenone, 2-PrOH, Δ, 15 h; ***vi*** isatin, 2-PrOH, Δ, 2 h; ***vii*** hexane-2,5-dione, 2-PrOH, AcOH, Δ, 4 h, H_2_O; ***viii*** pentane-2,4-dione, HCl, Δ, 2.5 h, H_2_O; ***ix*** 1,2-diphenyl-1,2-ethanedione, AcOH, NH_4_OAc, Δ, 24 h, H_2_O; 5% HCl, Δ, 5 min.

The synthesis was started from the preparation of key carboxylic acid **1a** (Supplementary materials, Figures S1 and S2), which was obtained from 2-aminophenol and itaconic acid according to the method described in [27]. The dichloro-substituted derivative **1b** was synthesized by treating 1-(2-hydroxyphenyl)-5-pyrrolidine-3-carboxylic acid (**1a**) with HCl in the presence of hydrogen peroxide (Scheme 1). To expand the library of 3-substituted 1-(2-hydroxy- and 2-hydroxy-3,5-dichlorophenyl)-5-oxopyrrolidines, compounds **1a, b** were esterified to the corresponding esters **2a** and **2b** with methanol in the presence of sulfuric acid as a catalyst. Then, the resulting ester **2a** (Supplementary materials, Figures S3 and S4) was reacted with hydrazine monohydrate in refluxing propan-2-ol. 

The reaction resulted in the formation of hydrazide **3** (Supplementary materials, Figures S5 and S6) with an 88.5% yield. To obtain hydrazones **4**–**15**, hydrazide **3** was applied for the condensation with 10 different aromatic aldehydes and thiophene-2-carbaldehyde, as well as its 5-nitro derivative. The reactions were carried out in propan-2-ol at reflux and afforded 1-(2-hydroxyphenyl)-*N*′-substituted-5-oxopyrrolidine-3-carbohydrazides at good to excellent yields (60–90%). Inspection of the ^1^H NMR spectra of compounds 4–15 (Supplementary Materials, Figures S7–S22, S63 and S64) revealed two sets of singlets corresponding to the CONH and CH=N protons, confirming the presence of a mixture of *Z* and *E* conformational isomers, caused by the restricted rotation around the amide bond, where as it is known [33,34], the *Z*-form usually predominates. The intense ratio of singlets of the rotamers appeared to be 65:35 for the synthesized hydrazones, while for compound **13** bearing the 1-naphthyl fragment, the ratio of the *Z*-form to *E*-form was found to be 60:40, describing a more stable molecule. Compound **16** was obtained by the reaction of acid hydrazide **3** with 1-(4-aminophenyl)ethan-1-one in propan-2-ol at reflux for 15 h (Supplementary materials, Figures S23 and S24). 

The 3-methyleneindolin-2-one moiety is a part of naturally occurring compounds and is widely used for the design of biologically active compounds [35,36]. Thus, to incorporate this structural unit into the designed structure, hydrazide **3** was treated with indoline-2,3-dione in refluxing propan-2-ol for 2 h. The product **17** was isolated with a 73% yield (Supplementary materials, Figures S25 and S26).

The target azole **18** and diazole **19** were synthesized via the acid-catalyzed condensation of **3** and hexane-2,5-dione or pentane-2,4-dione, respectively. The presence of drops of glacial acetic acid (**18**) or hydrochloric acid (**19**) led to the formation of the target *N*-(2,5-dimethyl-1*H*-pyrrol-1-yl)-1-(2-hydroxyphenyl)-5-oxopyrrolidine-3-carboxamide (**18**) or 4-(3,5-dimethyl-1*H*-pyrazole-1-carbonyl)-1-(2-hydroxyphenyl)pyrrolidin-2-one (**19**) (Supplementary materials, Figures S27–S30).

A mixture of carbohydrazide **3** and benzil in refluxing glacial acetic acid containing a 10-fold excess of ammonium acetate produced 1,2,4-triazine derivative **20**. The spectral and microanalysis data of the compound were in good agreement with the structure (Supplementary materials, Figures S31 and S32). 

**Scheme 2:**
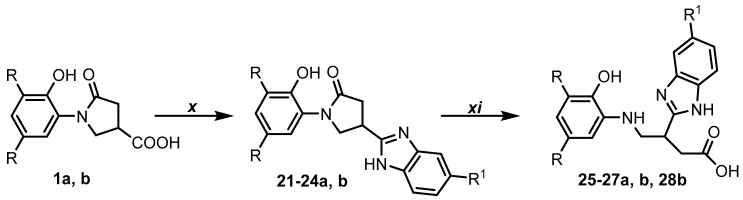
Synthesis of benzimidazole derivatives **21**–**28. a** R = H, **b** R = Cl; **21**, **25** R^1^ = H, **22**, **26** R^1^ = Me, **23**, **27** R^1^ = Cl, **24**, **28** R^1^ = F. *Reagents and conditions:* ***x*** the corresponding benzene-1,2-diamine, 6N HCl, Δ, 24 h; 15% NH_4_OH to pH 8; ***xi*** 20% NaOH, Δ, 4 h; diluted AcOH to pH 6.

Next, a series of benzimidazole derivatives variously substituted at 5^th^ position of the benzimidazole fragment was prepared and identified. The synthesis of compounds **21**–**24a, b** (Scheme 2) was accomplished via the condensation of carboxylic acids **1a, b** with the appropriate benzene-1,2-diamine in 6 M hydrochloric acid at reflux for 24 h. The corresponding benzimidazoles **21**–**24a, b** were isolated from the reaction mixtures through the alkalinization of the mixtures with 15% ammonium hydroxide to pH 8. From their ^1^H NMR spectra, a characteristic singlet in the range of 10.21–12.58 ppm proved the presence of the NH proton, and an increase in resonances in the aromatic area (^1^H and ^13^C) showed the presence of a new fused aromatic structure in the molecules. Finally, the obtained 5-oxopyrrolidine derivatives **21**–**24a, b** were applied for the preparation of the corresponding *γ*-amino acids **25**–**27a, b** and **28b** using the method described previously [37]. Due to the instability of the pyrrolidinone cycle in strong alkaline medium, the above-mentioned compounds **21**–**24a, b** were easily converted into the appropriate butanoic acids **25**–**28**. The comparison of the NMR spectra of study compounds **25**–**28** with the cleaved pyrrolidinone ring with the spectra of their parent cyclized analogues **21**–**24** showed the characteristic differences, as for instance, the resonances of the COOHs at approx. 173 ppm (^13^C NMR, **25**–**28**) are typical to the saturated open-chain carboxylic acids, while in the ^13^C NMR of cyclized derivatives **21**–**24**, the spectral lines of C=O resonated in the range of 171.89–172.54 ppm. Furthermore, the characteristic closer shifted spectral lines of the carbons of the alkyl chain NHCH_2_CHCH_2_CO in the **25**–**28**-series in comparison with the resonances of the corresponding carbons of the cyclized derivatives **21**–**24** were also observed (Supplementary materials, Figures S33–S62, S65 and S66).

### 2.2. Novel 1-(2-Hydroxyphenyl)-5-oxopyrrolidine-3-carboxylic Acid Derivatives Show Gram-Positive Bacteria-Directed Antimicrobial Activity

To understand the antimicrobial properties of novel 1-(2-hydroxyphenyl)-5-oxopyrrolidine-3-carboxylic acid derivatives **1a**–**28b** bearing hydrazone, azole, and azine moieties, libraries of multidrug-resistant bacterial and fungal pathogens were used for the screening assays. The pathogens were selected to represent major WHO-priority bacterial and fungal pathogens harboring genetically defined antimicrobial-resistance determinants [38]. The compounds **1a**–**28b** were screened using the broth microdilution technique with Clinical Laboratory Standard Institute recommendations. 

Carboxylic acids **1a, b** showed no antibacterial and antifungal activity (MIC > 128 µg/mL), while the transformation of compound **1b** to ester **2b** resulted in antimicrobial activity against methicillin-resistant *S. aureus* TCH 1516 (USA 300 lineage) (MIC 64 µg/mL) and New Delhi carbapenemase 1 (NDM-1)-producing *A. baumannii* AR-0033 (MIC 128 µg/mL) (Table 1). 

Compounds **3**–**6** showed no antibacterial or antifungal activity (MIC > 128 µg/mL), while hydrazone **7** (R = 4-O_2_NC_6_H_4_) showed activity against *S. aureus* TCH1516 (MIC 64 µg/mL) and *C. difficile* AR-1067 (MIC 16 µg/mL) (Table 1 and Table 2). Furthermore, the incorporation of the 4-Me_2_NC_6_H_4_ substituent (**8**) abolished antimicrobial activity against *C. difficile* AR-1067 (MIC > 128 µg/mL) without affecting antimicrobial activity against *S. aureus* TCH 1516 (MIC 64 µg/mL). In addition to that, the hydrazone **14**-bearing thien-2-yl group showed good activity against *S. aureus* TCH 1516 (MIC 16 µg/mL) and *C. difficile* AR-1067 (MIC 32 µg/mL), although no activity was observed against Gram-negative or fungal pathogens (MIC > 128 µg/mL) (Table 1 and Table 2). Interestingly, a 5-nitrothien-2-yl substitution (**15**) resulted in the loss of antibacterial activity (MIC > 128 µg/mL), although broad-spectrum antifungal activity was observed (Table 2). Compound **15** showed antifungal activity against three tested multidrug-resistant *C. auris* isolates representing major genetic lineages (MIC 16 µg/mL) (Table 2). Surprisingly, compound **15** showed antifungal activity against azole-resistant *A. fumigatus* strains with TR34/L98H mutations in the CYP51A gene (Table 2). On the other hand, compound **15** showed significant cytotoxicity in a non-cancerous HSAEC-1 pulmonary cell model, suggesting that the targets modulated by compound **15** are also found in pathogenic fungi (Figure S67). Triazine **20** showed activity against *S. aureus* TCH 1516 (MIC 32 µg/mL) and *P. aeruginosa* AR-0064, although no activity was observed against other tested bacterial or fungal pathogens (Table 1). 

Among benzimidazoles **21**–**24a, b,** compound **21b** (R = 3,5-diCl) showed activity against *S. aureus* TCH 1516 and *C. difficile* AR-1067 (MIC 32 and 64 µg/mL). 5-Methyl benzimidazole **22b** showed activity against *S. aureus* TCH 1516 (MIC 64 µg/mL), while 5-chloro benzimidazole **23a** showed considerably decreased antibacterial activity against *S. aureus* TCH 1516 (MIC 128 µg/mL). On the other hand, 5-chloro benzimidazole **23b** showed higher antibacterial activity against *S. aureus* TCH 1516 (MIC 64 µg/mL) in comparison to the **23a** analogue. Interestingly, 5-fluoro benzimidazole **24b** demonstrated broad-spectrum antibacterial activity against Gram-positive *S. aureus* TCH 1516 and *C. difficile* AR-1067 (MIC 8 and 128 µg/mL, respectively), as well Gram-negative NDM-1-producing *K. pneumoniae* AR-0049 (MIC 128 µg/mL) and *P. aeruginosa* AR-0064 (MIC 64 µg/mL) (Table 1). Furthermore, compound **24b** showed favorable low cytotoxicity in HSAEC-1 cells, making compound **24b** an attractive candidate for further hit-to-lead optimization (Figure S67). 

Among γ-amino acid derivatives **25**–**27a, b, 28b**, only low antibacterial (**25b,** MIC 128 µg/mL) activity against Gram-positive *S. aureus* TCH 1516 and *C. difficile* AR-1067 and antifungal (**26b** and **27b**) activity against the *C. auris* AR-381 isolate with the same MIC of 128 µg/mL were observed (Table 1 and Table 2).

### 2.3. Compounds ***14*** and ***24*b** Demonstrate Antibacterial Activity against Vancomycin-Intermediate Staphylococcus aureus Strains

After observing the promising antimicrobial activity of novel 3-substituted 1-(2-hydroxyphenyl)-5-oxopyrrolidines against multidrug-resistant *S. aureus*, we further evaluated if the most promising compounds **14** and **24b** are active against vancomycin-intermediate-resistant *S. aureus* strains with multiple pre-existing resistance mechanisms. To do so, we performed an antimicrobial activity determination of compounds **14** and **24b** against five representative strains with the vancomycin-intermediate-resistance phenotype. 

Compounds **14** and **24b**, bearing thien-2-yl and 5-fluoro benzimidazole substitutions, showed favorable activity against multidrug-resistant *S. aureus* strains with a vancomycin-intermediate-resistance phenotype and multiple resistance mechanisms (Table 3). The antibacterial activity of compound **14** (MIC 4–16 µg/mL) was comparable to that of vancomycin (VAN). Compound **24b** showed promising antibacterial (MIC 2–8 µg/mL) activity against *S. aureus* isolates with challenging antimicrobial resistance mechanisms and was comparable to the antimicrobial activity of daptomycin (DAP). 

Collectively, these results demonstrated that 3-substituted 1-(2-hydroxyphenyl)-5-oxopyrrolidines show promising antibacterial activity directed to *S. aureus* harboring a multidrug-resistant phenotype with emerging multidrug-resistance determinants. Compounds **14** and **24b** could be further explored for hit-to-lead optimization or as a scaffold for the development of new compounds with activity against vancomycin-intermediate *S. aureus*. 

### 2.4. Novel 1-(2-Hydroxyphenyl)-5-oxopyrrolidine-3-carboxylic Acid Derivatives Demonstrate Structure-Dependent Anticancer Activity

The in vitro anticancer activity of compounds 3-substituted 1-(2-hydroxyphenyl)-5-oxopyrrolidines **1a**–**28b** bearing hydrazone, azole, and azine moieties was determined using viability-based MTT assays. A549 cells were used as a well-described cell culture model to study non-small cell lung adenocarcinoma. The compound treatment-induced cytotoxicity was compared to that of cisplatin (CP), a powerful DNA-binding cytotoxic drug, and the S cycle inhibitor cytosine arabinoside (AraC). 

**Figure 1:**
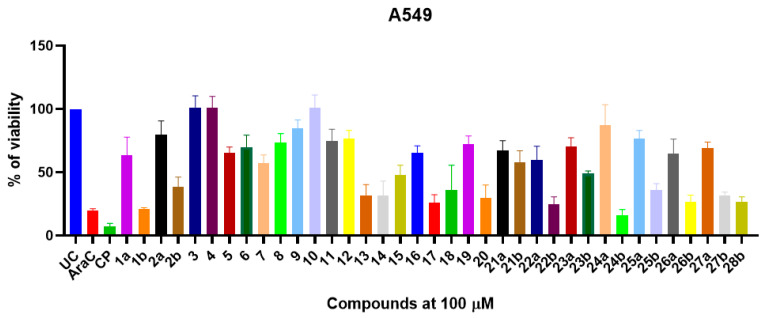
The viability of A549 non-small cell lung cancer cells after 24 h treatment with compounds 1a–28b and reference drugs: cisplatin (CP) and cytosine arabinoside (AraC) at a fixed concentration of 100 µM. The post-treatment viability was evaluated via MTT assays, and the % viability was calculated from the untreated controls. Data shown are the mean ± SD values from three separate experiments for each group.

To understand the cytotoxicity of compounds **1a**–**28b,** the A549 cells were exposed to a fixed 100 µM concentration of each compound or cytotoxicity control drugs (CP and AraC) for 24 h. The 3-substituted 1-(2-hydroxyphenyl)-5-oxopyrrolidines **1a**–**28b** demonstrated structure-dependent anticancer activity against A549 cells by affecting the A549 viability by 16.5–101% (Figure 1). The carboxylic acid **1a** (R = H) significantly reduced the A549 viability to 63.4% in comparison to that in the untreated controls (*p* < 0.05). The addition of 3,5-dichloro substitution (**1b**) greatly enhanced the in vitro anticancer activity by significantly reducing A549 viability to 21.2%. The ester **2a** was able to decrease A549 viability (71.3%), although no significant anticancer activity was observed. Interestingly, further transformation of **1b** to ester **2b** containing a 3,5-dichloro substitution on the phenyl ring was able to significantly (*p* < 0.001) decrease the anticancer activity against A549 cells (38.3%) (Figure 1). 

Hydrazide **3** and hydrazone **4** showed no anticancer activity and failed to significantly affect the A549 viability (101.2 and 101.3%, respectively). The addition of halogen substitutions on the phenyl ring resulted in compounds **5** and **6** with moderate anticancer activity. The hydrazone **5**-bearing 4-ClC_6_H_4_ substitution significantly decreased A549 viability (65.5%), while chlorine substitution with bromine (4-BrC_6_H_4_) (**5**) slightly decreased anticancer activity (69.9%) (*p* = 0.0001 and *p* = 0.001, respectively). The addition of a 4-O_2_NC_6_H_4_ substitution (**7**) enhanced anticancer activity (57.1%), while the incorporation of a 4-Me_2_NC_6_H_4_ moiety (**8**) resulted in decreased anticancer activity (73.8%) (*p* = 0.007) (Figure 1). Moreover, the addition of methoxy groups greatly affected the anticancer activity. The addition of 4-MeOC_6_H_4_ (**9**) or 2,4-di(MeO)C_6_H_3_ (**10**) moieties resulted in the loss of significant anticancer activity against A549 cells (84.8% and 100.9%, respectively). Interestingly, the addition of 2,3,4-tri(MeO)C_6_H_2_ (**11**) or 3,4,5-tri(MeO)C_6_H_2_ (**12**) moieties resulted in the restoration of anticancer activity, since compounds **11** and **12** containing an MeO substitution in the 2, 3, 4- and 3,4,5-positions of the phenyl ring resulted in 74.5% and 76.4% post-treatment viability (*p* = 0.0095 and *p* = 0.0254, respectively). On the other hand, the addition of 1-naphthyl (**13**) or thien-2-yl (**14**) strongly enhanced the anticancer activity of hydrazones by decreasing the viability to 31.4 and 31.7%, respectively (*p* < 0.0001). Moreover, further incorporation of a 5-nitrothien-2-yl moiety (**15**) resulted in the slight loss of anticancer activity (47.7%) (*p* < 0.001). The incorporation of an aminoacetophenone substitution (**16)** resulted in decreased anticancer activity (65.1%) (*p* < 0.001), while the addition of a 2-oxoindolin-3-ylidene fragment (**17**) resulted in strikingly enhanced anticancer activity (26.2%) (Figure 1). 

The pyrrole **18** and triazine **20** derivatives showed promising anticancer activity, while pyrazole **19** was found to decrease it. Compounds **18** and **20** significantly reduced A549 viability to 36.2 and 29.7%, respectively, while compound **19** only reduced the A549 viability to 72.4% (*p* < 0.05) (Figure 1). 

Benzimidazole derivatives **21**–**24a, b** were able to significantly reduce the viability of A549 cells (*p* < 0.05). Benzimidazole **21a** significantly reduced A549 viability by 67.4% (*p* = 0.003). The 5-methyl analogue **22a** reduced the A549 viability to 59.5%, while the addition of the 3,5-dichloro-2-hydroxyphenyl substituent (**22b**) significantly enhanced the anticancer activity by reducing the viability to 24.5% (*p* < 0.0001). Benzimidazole **23a**, containing a 5-Cl radical, demonstrated reduced anticancer activity (70.3%), while the incorporation of a 3,5-dichloro substitution on the 2-hydroxyphenyl fragment (**23b**) resulted in the restoration of anticancer activity (49.5%) (*p* < 0.0001). The 5-fluorobenzimidazole **24a** showed weak anticancer activity (87.4%), while the incorporation of a 3,5-dichloro-2-hydroxyphenyl substituent resulted in compound **24b** with strikingly increased anticancer activity (16.1%) (*p* < 0.0001). Notably, the anticancer effect exerted by compound **24b** was comparable to that of cytidine arabinoside (AraC, 19.7%) (Figure 1). 

Among synthesized γ-amino acid derivatives **25a**–**28b**, compounds were able to significantly decrease A549 viability in comparison to UC (*p* < 0.05). Compound **25a** reduced viability to 76.6% (*p* < 0.024), while the 3,5-dichloro derivative **25b** showed significantly increased anticancer activity (36.0%) (*p* < 0.0001). Furthermore, the addition of a 5-Me substituent (**26a, b**) or 5-Cl substituent (**27a, b**) did not significantly affect anticancer activity in comparison to that of the primary compounds, while the 5-F analogue **28b** showed slightly stronger anticancer activity against A549 cells (Figure 1). 

## 3. Materials and Methods

### 3.1. Synthesis 

Reagents, antibiotics, and solvents were obtained from Sigma-Aldrich (St. Louis, MO, USA) and used without further purification. The reaction course and purity of the synthesized compounds were monitored via TLC using aluminum plates precoated with Silica gel with F254 nm (Merck KGaA, Darmstadt, Germany). Melting points were determined with a B-540 melting point analyzer (Büchi Corporation, New Castle, DE, USA) and were uncorrected. NMR spectra were recorded on a Brucker Avance III (400, 101 MHz) spectrometer (Bruker BioSpin AG, Fällanden, Switzerland). Chemical shifts were reported in (d) ppm relative to tetramethylsilane (TMS) with the residual solvent as an internal reference (DMSO-*d_6_*, δ = 2.50 ppm for ^1^H and d = 39.5 ppm for ^13^C). Data were reported as follows: chemical shift, multiplicity, coupling constant (Hz), integration, and assignment. IR spectra (ν, cm^−1^) were recorded on a Perkin–Elmer Spectrum BX FT–IR spectrometer (Perkin– Elmer Inc., Waltham, MA, USA) using KBr pellets. Mass spectra were obtained on a Bruker maXis UHR-TOF mass spectrometer (Bruker Daltonics, Bremen, Germany) with ESI ionization. Elemental analyses (C, H, N) were conducted using the Elemental Analyzer CE-440 (Exeter Analytical, Inc., Chelmsford, MA, USA); their results were found to be in good agreement (±0.3%) with the calculated values.

*1-(2-Hydroxyphenyl)-5-oxopyrrolidine-3-carboxylic acid* (**1a**). To a solution of itaconic acid (6.5 g, 50 mmol) in water (16 mL), *o*-aminophenol (4.91 g, 45 mmol) was added and the mixture was refluxed for 12 h, then cooled down, and the formed precipitate was filtered off, washed with water, and dried. 

Pale brown solid, yield 74.4%, m.p. 178–179 °C (from water). 

^1^H NMR (DMSO-*d_6_*, 400 MHz), δ: 2.56–2.72 (m, 2H, CH_2_CO), 3.34–3.42 (m, 1H, CH), 3.78–3.95 (m, 2H, NCH_2_), 6.80 (t, *J* = 7.5 Hz, 1H, H_arom_), 6.90 (d, *J* = 8.2 Hz, 1H, H_arom_), 7.10–7.24 (m, 2H, H_arom_), 9.56 (s, 1H, OH), 12.71 (s, 1H, COOH) ppm.

^13^C NMR (DMSO-*d_6_*, 101 MHz), δ: 33.71, 36.23, 50.96 (CH_2_CO, CH, NCH_2_), 116.80, 119.18, 125.50, 128.21, 128.30, 152.69 (C_arom_), 172.20, 174.37 (2C=O) ppm.

IR (KBr), ν_max_: 1633, 1737 (2CO); 3115 (OH) cm^−1^.

Calcd. for C_11_H_11_NO_4_, %: C 59.73; H 5.01; N 6.33. Found, %: C 60.09; H 5.14; N 6.54.

*1-(3*,*5-Dichloro-2-hydroxyphenyl)-5-oxopyrrolidine-3-carboxylic acid* (**1b**). To a mixture of acid **1a** (13.27 g, 60 mmol), conc. HCl (15 mL) and water (120 mL) 30% H_2_O_2_ was added dropwise over 5 min, and the mixture was stirred at 50 °C for 2 h. Then, the mixture was cooled down, and the formed precipitate was filtered off and purified by dissolving it in 5% sodium hydroxide solution, filtering and acidifying the filtrate with hydrochloric acid to pH 1–2.

The experimental data of **1b** are in excellent agreement with those reported in [33].

*General procedure of the preparation of esters* **2a, b**

A mixture of the corresponding carboxylic acid **2a, b** (85 mmol), methanol (150 mL), and sulfuric acid (2 mL) was heated at reflux for 2 h and then cooled down and evaporated at reduced pressure. The residue was poured with aqueous 5% sodium carbonate solution (50 mL) and stirred for 5 min and left to cool down. The formed precipitated was filtered off, washed with water, and dried.

*Methyl 1-(2-hydroxyphenyl)-5-oxopyrrolidine-3-carboxylate* (**2a**). *Light grey solid, yield 78.8%, m.p. 128–129 °C (from MeOH)*.

^1^H NMR (DMSO-*d_6_*, 400 MHz), δ: 2.60–2.73 (m, 2H, CH_2_CO), 3.45–3.53 (m, 1H, CH), 3.68 (s, 3H, OCH_3_), 3.80–3.93 (m, 2H, NCH_2_), 6.80 (t, *J* = 7.6 Hz, 1H, H_arom_), 6.89 (d, *J* = 8.3 Hz, 1H, H_arom_), 7.09–7.19 (m, 2H, H_arom_), 9.59 (s, 1H, OH) ppm.

^13^C NMR (DMSO-*d_6_*, 101 MHz), δ: 33.55, 36.00, 50.69 (CH_2_CO, CH, NCH_2_), 52.11 (OCH_3_), 116.74, 119.12, 125.33, 128.22, 128.316, 152.68 (C_arom_), 171.84, 173.25 (2C=O) ppm.

IR (KBr), ν_max_: 1656, 1741 (2CO); 3073 (OH) cm^−1^.

Calcd. for C_12_H_13_NO_4_, %: C 61.27; H 5.57; N 5.95. Found, %: C 61.03; H 5.80; N 6.13.

*Methyl 1-(3,5-dichloro-2-hydroxyphenyl)-5-oxopyrrolidine-3-carboxylate* (**2b**).

White solid, yield 78.8%, m.p. 149–150 °C (from MeOH).

^1^H NMR (DMSO-*d_6_*, 400 MHz), δ: 2.59–2.71 (m, 2H, CH_2_CO), 3.41–3.49 (m, 1H, CH), 3.69 (s, 3H, OCH_3_), 3.81–3.87 (m, 2H, NCH_2_), 7.27 (s, 1H, H_arom_), 7.49 (s, 1H, H_arom_), 9.61 (s, 1H, OH) ppm.

^13^C NMR (DMSO-*d_6_*, 101 MHz), δ: 33.56, 35.98, 50.70 (CH_2_CO, CH, NCH_2_), 52.18 (OCH_3_), 122.38, 122.49, 126.81, 128.13, 128.34, 148.52 (C_arom_), 172.04, 173.10 (2C=O) ppm.

IR (KBr), ν_max_: 1659, 1753 (2CO); 3172 (OH) cm^−1^.

Calcd. for C_12_H_11_Cl_2_NO_4_, %: C 47.39; H 3.65; N 4.61. Found, %: C 47.44; H 3.61; N 4.66.

*1-(2-Hydroxyphenyl)-5-oxopyrrolidine-3-carbohydrazide* (**3**).

A mixture of ester **2** (8.5 g, 35 mmol), hydrazine monohydrate (5 g, 10 mmol), and propan-2-ol (150 mL) was heated at reflux for 2.5 h and then cooled down. The formed precipitate was filtered and washed with propan-2-ol and dried.

Brown solid, yield 88.5%, m.p. 188–189 °C (from 2-PrOH).

^1^H NMR (DMSO-*d_6_*, 400 MHz), δ: 2.51–2.62 (m, 2H, CH_2_CO), 3.15–3.26 (m, 1H, CH), 3.66–3.83 (m, 2H, NCH_2_), 4.32 (s, 2H, NH_2_), 6.81 (t, *J* = 7.5 Hz, 1H, H_arom_), 6.91 (d, *J* = 8.2 Hz, 1H, H_arom_), 7.10–7.16 (m, 2H, H_arom_), 9.29 (s, 1H, NH), 9.58 (s, 1H, OH) ppm.

^13^C NMR (DMSO-*d_6_*, 101 MHz), δ: 34.38, 35.59, 51.62 (CH_2_CO, CH, NCH_2_), 116.77, 119.18, 125.47, 128.26, 128.36, 152.77 (C_arom_), 171.77, 172.43 (2C=O) ppm.

IR (KBr), ν_max_: 1682 (2CO); 2967–3330 (OH, NH, NH_2_) cm^−1^.

Calcd. for C_11_H_13_N_3_O_3_, %: C 56.16; H 5.57; N 17.86. Found, %: C 56.52; H 5.66; N 18.11.

*General procedure for the preparation of hydrazones* **4–15**

To a hot solution of hydrazide **3** (0.7 g, 3 mmol) in propan-2-ol (15 mL), the corresponding aromatic or non-aromatic aldehyde (4 mmol) was added, and the mixture was heated at reflux for 2 h or 40 min for **14** and then cooled down. The obtained solid was filtered off, washed with propan-2-ol, and dried to give the title compounds **4–15**.

*N′-benzylidene-1-(2-hydroxyphenyl)-5-oxopyrrolidine-3-carbohydrazide* (**4**).

White solid, yield 73%, m.p. 224–225 °C (from 2-PrOH).

^1^H NMR (DMSO-*d_6_*, 400 MHz), δ: *Z/E* 65/35, 2.62–2.83 (m, 2H, CH_2_CO), 3.36–3.45 (m, 0.35H, CH), 3.78–4.18 (m, 2H, NCH_2_, 0.65H, CH), 6.82 (t, *J* = 7.5 Hz, 1H, NH), 6.87–6.95 (m, 1H, H_arom_), 7.08–7.19 (m, 2H, H_arom_), 7.35–7.51 (m, 3H, H_arom_), 7.62–7.74 (m, 2H, H_arom_), 8.03, 8,22 (2s, 1H, CH=N), 9.58, 9.60 (2s, 1H, OH), 11.56, 11.61 (2s, 1H, NH) ppm. 

^13^C NMR (DMSO-*d_6_*, 101 MHz), δ: 33.29, 34.15, 34.17, 36.24, 51.08, 51.43 (CH_2_CO, CH, NCH_2_), 116.74, 116.79, 119.16, 125.45, 125.55, 126.82, 127.10, 128.22, 128.28, 128.83, 128.87, 129.90, 130.13, 134.14, 143.57, 147.03, 152.66, 152.72, 168.75, 172.22, 172.41, 173.68 (C_arom_, N=CH, 2C=O) ppm.

IR (KBr), ν_max_: 1588 (C=N); 1671 (2CO); 2959–3183 (OH, NH) cm^−1^.

Calcd. for C_18_H_17_N_3_O_3_, %: C 66.86; H 5.30; N 13.00. Found, %: C 66.92; H 5.23; N 13.08.

*N′-(4-chlorobenzylidene)-1-(2-hydroxyphenyl)-5-oxopyrrolidine-3-carbohydrazide* (**5**).

White solid, yield 60%, m.p. 209–210 °C (from 2-PrOH).

^1^H NMR (DMSO-*d_6_*, 400 MHz), δ: *Z/E* 65/35, 2.55–2.78 (m, 2H, CH_2_CO), 3.35–3.42 (m, 0.35H, CH), 3.76–4.18 (m, 2H, NCH_2_, 0.65H, CH), 6.82 (t, *J* = 7.5 Hz, 1H, H_arom_), 6.91 (d, *J* = 8.0 Hz, 1H, H_arom_), 7.03–7.26 (m, 2H, H_arom_), 7.50 (t, *J* = 8.1 Hz, 2H, H_arom_), 7.63–7.81 (m, 2H, H_arom_), 8.01, 8.20 (2s, 1H, CH=N), 9.58, 9.60 (2s, 1H, OH), 11.62, 11.67 (2s, 1H, NH) ppm.

IR (KBr), ν_max_: 1593 (C=N); 1671 (2CO); 2953–3074 (OH, NH) cm^−1^.

Calcd. for C_18_H_16_ClN_3_O_3_, %: C 60.43; H 4.51; N 11.74. Found, %: C 60.35; H 4.46; N 11.73.

*N′-(4-bromobenzylidene)-1-(2-hydroxyphenyl)-5-oxopyrrolidine-3-carbohydrazide* (**6**).

White solid, yield 90%, m.p. 217–218 °C (from 2-PrOH).

^1^H NMR (DMSO-*d_6_*, 400 MHz), δ: *Z/E* 65/35, 2.59–2.85 (m, 2H, CH_2_CO), 3.35–3.44 (m, 0.35H, CH), 3.76–4.19 (m, 2H, NCH_2_, 0.65H, CH), 6.82 (t, *J* = 7.5 Hz, 1H, H_arom_), 6.91 (d, *J* = 8.0 Hz, 1H, H_arom_), 6.97–7.34 (m, 2H, H_arom_), 7.38–7.87 (m, 4H, H_arom_), 8.00, 8.19 (2s, 1H, CH=N); 9.58, 9.59 (2s, 1H, OH), 11.62; 11.68 (2s, 1H, NH) ppm.

^13^C NMR (DMSO-*d_6_*, 101 MHz), δ: 33.29, 34.10, 34.14, 36.22, 51.01, 51.39 (CH_2_CO, CH, NCH_2_), 116.74, 116.78, 119.15, 123.36, 125.44, 125.53, 128.21, 128.27, 128.70, 131.84, 133.44, 142.39, 145.78, 152.64, 152.71,168.83, 172.18, 172.35, 173.74 (C_arom_, N=CH, 2C=O) ppm.

IR (KBr), ν_max_: 1592 (C=N); 1671 (2CO); 2954–3076 (OH, NH) cm^−1^.

Calcd. for C_18_H_16_BrN_3_O_3_, %: C 53.75; H 4.01; N 10.45. Found, %: C 53.69; H 3.93; N 10.39.

*1-(2-Hydroxyphenyl)-N′-(4-nitrobenzylidene)-5-oxopyrrolidine-3-carbohydrazide* (**7**).

Light yellow solid, yield 83.6%, m.p. 210–211 °C (from 2-PrOH).

^1^H NMR (400 MHz, DMSO-*d_6_*), δ: *Z/E* 65/35, 2.63–2.82 (m, 2H, CH_2_CO), 3.39–3.48 (m, 0.35H, CH), 3.79–4.19 (m, 2H, NCH_2_, 0.65H, CH), 6.82 (t, *J* = 7.6 Hz, 1H, H_arom_), 6.91 (d, *J* = 8.2 Hz, 1H, H_arom_), 7.05–7.22 (m, 2H, H_arom_),7.88–8.03 (m, 2H, H_arom_), 8.12 (s, 0.65H, HCN); 8.18–8.37 (m, 2H, H_arom_, 0.35H, CH=N), 9.61 (s, 1H, OH), 11.85; 11.90 (2s, 1H, NH) ppm.

IR (KBr), ν_max_: 1586 (C=N); 1673 (2CO); 2926–3079 (OH, NH) cm^−1^.

Calcd. for C_18_H_16_N_4_O_5_, %: C 58.69; H 4.38; N 15.21. Found, %: C 58.59; H 4.41; N 15.14.

*N′-(4-(dimethylamino)benzylidene)-1-(2-hydroxyphenyl)-5-oxopyrrolidine-3-carbohydrazide* (**8**).

Pale yellow solid, yield 78.1%, m.p. 170–171 °C (from 2-PrOH).

^1^H NMR (DMSO-*d_6_*, 400 MHz), δ: *Z/E* 65/35, 2.59–2.80 (m, 2H, CH_2_CO), 2.95, 2.96 (2s, 6H, N(CH_3_)_2_), 3.75–4.10 (m, 2H, NCH_2_, 1H, CH), 6.73 (t, *J* = 7.6 Hz, 2H, H_arom_), 6.82 (t, *J* = 7.5 Hz, 1H, H_arom_), 6.86–6.96 (m, 1H, H_arom_), 7.10–7.19 (m, 2H, H_arom_), 7.44–7.54 (m, 2H, H_arom_), 7.89, 8.06 (2s, 1H, CH=N), 9.57, 9.60 (2s, 1H, OH), 11.27, 11.30 (2s, 1H, NH) ppm.

IR (KBr), ν_max_: 1599 (C=N); 1659 (2CO); 2973–3442 (OH, NH) cm^−1^.

Calcd. for C_20_H_22_N_4_O_3_, %: C 65.56; H 6.05; N 15.29. Found, %: C 65.49; H 6.09; N 15.37.

*1-(2-Hydroxyphenyl)-N′-(4-methoxybenzylidene)-5-oxopyrrolidine-3-carbohydrazide* (**9**).

Light pink solid, yield 78%, m.p. 157–158 °C (from 2-PrOH).

^1^H NMR (DMSO-*d_6_*, 400 MHz), δ: *Z/E* 65/35, 2.58–2.87 (m, 2H, CH_2_CO), 3.36–3.41 (m, 0.35H, CH), 3.78, 3.80 (2s, 3H, OCH_3_), 3.82–4.15 (m, 2H, NCH_2_, 0.65H, CH), 6.82 (t, *J* = 7.5 Hz, 1H, H_arom_), 6.89–7.20 (m, 5H, H_arom_), 7.63 (t, *J* = 8.8 Hz, 1H, H_arom_), 7.97, 8.15 (2s, 1H, CH=N), 9.58, 9.60 (2s, 1H, OH), 11.43, 11.48 (2s, 1H, NH) ppm.

IR (KBr), ν_max_: 1592 (C=N); 1668 (2CO); 2952–3473 (OH, NH) cm^−1^.

Calcd. for C_19_H_19_N_3_O_4_, %: C 64.58; H 5.42; N 11.89. Found, %: C 64.50; H 5.48; N 11.81.

*1-(2-Hydroxyphenyl)-N′-(2,4-dimethoxybenzylidene)-5-oxopyrrolidine-3-carbohydrazide* (**10**).

White solid, yield 66.6%, m.p. 149–150 °C (from 2-PrOH).

^1^H NMR (DMSO-*d_6_*, 400 MHz), δ: *Z/E* 65/35, 2.58–2.80 (m, 2H, CH_2_CO), 3.28–3.34 (m, 0.35H, CH), 3.80, 3.81, 3.83, 3.85 (4s, 6H, 2OCH_3_), 3.84–4.14 (m, 2H, NCH_2_, 0.65H, CH), 6.55–6.66 (m, 2H, H_arom_), 6.76–7.96 (m, 2H, H_arom_), 7.05–7.22 (m, 2H, H_arom_), 7.74 (d, *J* = 8.5 Hz, 1H, H_arom_), 8.27, 8.46 (2s, 1H, CH=N), 9.58 (s, 1H, OH), 11.37, 11.46 (2s, 1H, NH) ppm.

IR (KBr), ν_max_: 1589 (C=N); 1670 (2CO); 2841–3182 (OH, NH) cm^−1^.

Calcd. for C_20_H_21_N_3_O_5_, %: C 62.65; H 5.52; N 10.96. Found, %: C 62.58; H 5.52; N 10.89.

*1-(2-Hydroxyphenyl)-N′-(2,3,4-trimethoxybenzylidene)-5-oxopyrrolidine-3-carbohydrazide* (**11**).

White solid, yield 87.2%, m.p. 234–235 °C (from 2-PrOH).

^1^H NMR (DMSO-*d_6_*, 400 MHz), δ: *Z/E* 65/35 2.61–2.80 (m, 2H, CH_2_CO), 3.35–3.41 (m, 0.35H, CH), 3.76, 3.77, 3.81, 3.82, 3.84 (5s, 9H, 3OCH_3_), 3.86–4.15 (m, 2H, NCH_2_, 0.65H, CH), 6.78–6.95 (m, 3H, H_arom_), 7.09–7.19 (m, 2H, H_arom_), 7.56 (dd, *J* = 8.8, 5.6 Hz, 1H, H_arom_), 8.21, 8.37 (2s, 1H, CH=N), 9.57, 9.59 (2s, 1H, OH), 11.43, 11.55 (2s, 1H, NH) ppm.

IR (KBr), ν_max_: 1590 (C=N); 1669 (2CO); 2948–3322 (OH, NH) cm^−1^.

Calcd. for C_21_H_23_N_3_O_6_, %: C 61.01; H 5.61; N 10.16. Found, %: C 60.97; H 5.68; N 10.12.

*1-(2-Hydroxyphenyl)-N′-(3,4,5-trimethoxybenzylidene)-5-oxopyrrolidine-3-carbohydrazide* (**12**).

Light grey solid, yield 82.3%, m.p. 166–167 °C (from 2-PrOH).

^1^H NMR (DMSO-*d_6_*, 400 MHz), δ: *Z/E* 65/35 2.61–2.84 (m, 2H, CH_2_CO), 3.37–3.42 (m, 0.35H, CH), 3.68, 3.70 (2s, 3H, OCH_3_), 33.80, 3.82, (2s, 6H, 2OCH_3_), 3.83–4.16 (m, 2H, NCH_2_, 0.65H, CH), 6.82 (q, *J* = 7.2 Hz, 1H, H_arom_), 6.87–6.94 (m, 1H, H_arom_), 6.98, 7.01 (2s, 2H, H_arom_), 7.08–7.20 (m, 2H, H_arom_), 7.93, 8.13 (2s, 1H, CH=N), 9.57, 9.61 (2s, 1H, OH), 11.58, 11.59 (2s, 1H, NH) ppm.

IR (KBr), ν_max_: 1580 (C=N); 1663 (CO); 2940–3540 (OH, NH) cm^−1^.

Calcd. for C_21_H_23_N_3_O_6_, %: C 61.01; H 5.61; N 10.16. Found, %: C 61.11; H 5.68; N 10.09.

*1-(2-Hydroxyphenyl)-N′-(naphth-1-ylmethylene)-5-oxopyrrolidine-3-carbohydrazide* (**13**).

White solid, yield 83.1%, m.p. 184–186 °C (from 2-PrOH).

^1^H NMR (DMSO-*d_6_*, 400 MHz), δ: *Z/E* 65/35, 2.58–2.94 (m, 2H, CH_2_CO), 3.39–3.51 (m, 0.4H, CH), 3.76–4.16 (m, 2H, NCH_2_), 4.16–4.32 (m, 0.6H, CH), 6.83 (q, *J* = 7.3 Hz, 1H, H_arom_), 6.92 (t, *J* = 8.0 Hz, 1H, H_arom_), 7.00–7.30 (m, 2H, H_arom_), 7.44–8.20 (m, 6H, H_arom_), 8.59, 8.87 (2d, *J* = 8.5 Hz, 1H, H_arom_), 8.73, 8.83 (2s, 1H, CH=N), 9.60 (s, 1H, OH), 11.61, 11.73 (2s, 1H, NH) ppm.

IR (KBr), ν_max_: 1587 (C=N); 1665 (2CO); 2969–3176 (OH, NH) cm^−1^.

Calcd. for C_22_H_19_N_3_O_3_, %: C 70.76; H 5.13; N 11.25. Found, %: C 70.70; H 5.19; N 11.18.

*1-(2-Hydroxyphenyl)-5-oxo-N′-(thien-2-ylmethylene)pyrrolidine-3-carbohydrazide* (**14**).

Light yellow solid, yield 85%, m.p. 224–225 °C (from 2-PrOH).

^1^H NMR (DMSO-*d_6_*, 400 MHz), δ: *Z/E* 65/35 2.60–2.81 (m, 2H, CH_2_CO), 3.34–3.41 (m, 0.35H, CH), 3.73–4.08 (m, 2H, NCH_2_, 0,65H, CH), 6.77–6.96 (m, 2H, H_arom_), 7.01–7.25 (m, 3H, H_arom_), 7.41, 7.46 (2d, *J* = 3.2 Hz, 1H, H_arom_), 7.61, 7.66 (2d, *J* = 5.0 Hz, 1H, H_arom_), 8.19, 8.42 (2s, 1H, CH=N), 9.58, 9.59 (2s, 1H, OH), 11.54, 11.56 (2s, 1H, NH) ppm.

^13^C NMR (DMSO-*d_6_*, 101 MHz), δ: 33.14, 34.12, 34.24, 35.22, 51.05,51.42 (CH_2_CO, CH, NCH_2_),116.73, 116.77, 119.16, 125.44, 125.50, 127.86, 127.98, 128.26, 128.43, 129.01, 130.38, 13,108, 138.57, 142.20, 152.71, 168.59, 172.34, 173.26 (C_arom_, N=CH, 2C=O) ppm.

IR (KBr), ν_max_: 1589 (C=N); 1670 (2CO); 2883–3097 (OH, NH) cm^−1^.

HRMS (ESI) for C_16_H_15_N_3_O_3_S + H^+^, calcd. 330.0912, found 330.0908 [M + H^+^].

Calcd. for C_16_H_15_N_3_O_3_S, %: C 58.35; H 4.59; N 12.76. Found, %: C 58.49; H 4.62; N 12.79.

*1-(2-Hydroxyphenyl)-5-oxo-N′-(5-nitrothien-2-ylmethylene)pyrrolidine-3-carbohydrazide* (**15**).

White solid, yield 82.9%, m.p. 172–173 °C (from 2-PrOH).

^1^H NMR (DMSO-*d_6_*, 400 MHz), δ: *Z/E* 65/35 2.62–2.81 (m, 2H, CH_2_CO), 3.38–3.45 (m, 0.35H, CH), 3.75–4.10 (m, 2H, NCH_2_, 0.65H, CH), 6.82 (t, *J* = 7.5 Hz, 1H, H_arom_), 6.91 (d, *J* = 8.1 Hz, 1H, H_arom_), 7.04–7.26 (m, 2H, H_arom_), 7.04–7.26 (m, 2H, H_arom_), 7.52, 7.56 (2d, *J* = 4.2 Hz, 1H, H_arom_), 8.11 (dd, *J* = 6.6, 4.5 Hz, 1H, H_arom_), 8.19, 8.47 (2s, 1H, CH=N), 9.58, 9.60 (2s, 1H, OH), 11.96 (s, 1H, NH) ppm.

^13^C NMR (DMSO-*d_6_*, 101 MHz), δ: 33.98, 34.05, 36.28, 50.93, 51.24 (CH_2_CO, CH, NCH_2_), 116.73, 116.79, 119.17, 125.46, 128.29, 129.20, 130.65, 136.83. 140.56, 146.53, 146.58, 150.53, 150.88, 152.72, 169.25, 172.06, 172.22, 173.92 (C_arom_, N=CH, 2C=O) ppm.

IR (KBr), ν_max_: 1565 (C=N); 1663 (2CO); 2965–3183 (OH, NH) cm^−1^.

HRMS (ESI) for C_16_H_14_N_4_O_5_S + Na^+^, calcd. 397.0583, found 397.0577 [M + Na^+^].

Calcd. For C_16_H_14_N_4_O_5_S, %: C 51.33; H 3.77; N 14.97. Found, %: C 51.40; H 3.85; N 14.89.

*N′-(1-(4-aminophenyl)ethylidene)-1-(2-hydroxyphenyl)-5-oxopyrrolidine-3-carbohydrazide* (**16**)

A mixture of hydrazide 3 (1.18 g, 5 mmol), 1-(4-aminophenyl)ethan-1-one (0.68 g, 5 mmol) and propan-2-ol (15 mL) was heated at reflux for 15 h, then cooled down, and the formed precipitate was filtered of, washed with propan-2-ol, and dried. 

White solid, yield 72.2%, m.p. 226–227 °C (from 2-PrOH).

^1^H NMR (DMSO-*d_6_*, 400 MHz), δ: *Z/E* 65/35, 2.15, 2.17 (2s, 3H, CH_3_), 2.59–2.81 (m, 2H, CH_2_CO), 3.52–3.61 (m, 0.35H, CH), 3.72–4.19 (m, 2H, NCH_2_, 0.65H, CH), 5.43, 5.47 (2s, 2H, NH_2_), 6.55 (d, *J* = 8.3 Hz, 2H, H_arom_), 6.74–7.00 (m, 2H, H_arom_), 7.03–7.24 (m, 2H, H_arom_), 7.34–7.66 (m, 2H, H_arom_), 9.57, 9.60 (2s, 1H, OH), 10.34, 10.47 (2s, 1H, NH) ppm.

^13^C NMR (DMSO-*d_6_*, 101 MHz), δ: *Z/E* 65/35, 13.24, 13.81 (CH_3_), 33.39, 34.42, 36.03 (CH_2_CO, CH), 51.29, 51.84 (NCH_2_), 113.11, 113.26, 116.72, 116.79, 119.15, 125.02, 125.22, 125.51, 125.57, 127.19, 127.61, 128.19, 128.27, 148.98, 150.01, 150.27, 152.64, 152.72, 153.78 (C_arom_), 168.80, 172.43, 172.57, 174.02 (C=O) ppm.

IR (KBr), ν_max_: 1591 (C=N); 1645; 1666 (CO); 2960–3464 (OH, NH) cm^−1^.

Calcd. for C_19_H_20_N_4_O_3_, %: C 64.76; H 5.72; N 15.90. Found, %: C 64.69; H 5.80; N 15.82.

*1-(2-Hydroxyphenyl)-5-oxo-N′-(2-oxoindolin-3-ylidene)pyrrolidine-3-carbohydrazide* (**17**).

White solid, yield 72.6%, m.p. 196–197 °C (from 2-PrOH).

^1^H NMR (DMSO-*d_6_*, 400 MHz), δ: *Z/E* 65/35, 2.65–2.84 (m, 2H, CH_2_CO), 3.83–4.37 (m, 2H, NCH_2_, 1H, CH), 6.82 (t, *J* = 7.6 Hz, 1H, H_arom_), 6.86–6.96 (m, 2H, H_arom_), 6.98–7.18 (m, 3H, H_arom_), 7.38 (t, *J* = 7.9 Hz, 1H, H_arom_), 8,07, 8.13 (2s, 1H, H_arom_), 9.60 (s, 1H, OH), 10.81 (s, 1H, NH), 11.35 (s, 1H, NH) ppm.

^13^C NMR (DMSO-*d_6_*, 101 MHz), δ: 33.22, 34.63 (CH_2_CO, CH), 51.12 (NCH_2_), 110.63, 115.21, 116.76, 119.17, 121.71, 125.46, 126.09, 126.41, 128.32, 132.60, 132.96, 143.85, 152.75, 164.58, 172.11 (C_arom_, 3C=O) ppm. 

IR (KBr), ν_max_: 1601 (C=N); 1694, 1716, 1732 (3CO); 3023–3380 (OH, NH) cm^−1^.

Calcd. for C_19_H_16_N_4_O_4_, %: C 62.63; H 4.43; N 15.38. Found, %: C 62.57; H 4.51; N 15.29.

*1-(2-Hydroxyphenyl)-N-(2,5-dimethyl-1H-pyrrol-1-yl)-5-oxopyrrolidine-3-carboxamide* (**18**).

To a solution of hydrazide **3** (0.7 g, 3 mmol) in propan-2-ol (15 mL), hexane-2,5-dione (1.10 g, 9.5 mmol) and acetic acid (3 drops) were added, and the mixture was refluxed for 4 h. After completion of the reaction (TLC), the cooled mixture was diluted with water (25 mL) and stirred for 15 min. The formed precipitate was filtered of, washed with propan-2-ol, and dried. 

Light yellow solid, yield 94.4%, m.p. 197–198 °C (from 2-PrOH).

^1^H NMR (DMSO-*d_6_*, 400 MHz), δ: 1.98, 2.01 (2s, 6H, 2CH_3_), 2.71 (d, J = 8.4 Hz, 2H, CH_2_CO), 3.46–3.58 (m, 1H, CH), 3.80–4.03 (m, 2H, NCH_2_), 5.66 (s, 2H, H_pyrr_), 6.83 (t, *J* = 7.6 Hz, 1H, H_arom_), 6.92 (d, *J* = 8.3 Hz, 1H, H_arom_), 7.08–7.19 (m, 2H, H_arom_), 9.62 (s, 1H, OH), 10.87 (s, 1H, NH) ppm.

^13^C NMR (DMSO-*d_6_*, 101 MHz), δ: 10.93, 10.97 (2CH_3_); 33.76, 35.31 (CH_2_CO, CH), 51.42 (NCH_2_), 103.10, 116.72, 119.18, 125.35, 126.75, 126.78, 128.37, 152.77 (C_arom_), 171.87, 172.06 (2C=O) ppm.

IR (KBr), ν_max_: 1666 (2CO); 2978–3272 (OH, NH) cm^−1^.

Calcd. for C_17_H_19_N_3_O_3_, %: C 65.16; H 6.11; N 13.41. Found, %: C 65.24; H 6.02; N 13.37.

*1-(2-Hydroxyphenyl)-4-(3,5-dimethyl-1H-pyrazol-1-carbonyl)pyrrolidin-2-one* (**19**).

To a solution of hydrazide **3** (0.7 g, 3 mmol) in propan-2-ol (15 mL), pentane-2,4-dione (0.6 g, 6 mmol) and hydrochloric acid (3 drops) were added, and the mixture was refluxed for 2.5 h. After completion of the reaction (TLC), the cooled mixture was diluted with water (25 mL) and stirred for 15 min. The formed precipitate was filtered of, washed with propan-2-ol, and dried. 

White solid, yield 68.3%, m.p. 139–140 °C (from 2-PrOH).

^1^H NMR (DMSO-*d_6_*, 400 MHz), δ: 2.19 (s, 3H, CH_3_), 2.50 (s, 3H, CH3, overlaps with the signal of the DMSO-*d_6_*), 2.64–2.86 (m, 2H, CH_2_CO); 3.82–4.09 (m, 2H, NCH_2_), 4.15–4.85 (m, 1H, CH), 6.22 (s, 1H, H_pyr_), 6.81 (t, *J* = 7.6 Hz, 1H, H_arom_), 6.90 (d, *J* = 7.9 Hz, 1H, H_arom_), 7.08–7.21 (m, 2H, H_arom_), 9.59 (s, 1H, OH) ppm.

^13^C NMR (DMSO-*d_6_*, 101 MHz), δ: 13.55, 14.08 (CH_3_), 33.46, 36.74 (CH_2_CO, CH), 51.21 (NCH_2_), 111.52 (CH_pyr_), 116.72, 119.14, 125.33, 128.32, 143.82, 152.07, 152.70 (C_arom_, C_pyr_), 171.84, 172.60 (2C=O) ppm.

IR (KBr), ν_max_: 1584 (C=N); 1660; 1725 (CO); 2932–3426 (OH) cm^−1^.

Calcd. for C_16_H_17_N_3_O_3_, %: C 64.20; H 5.72; N 14.04. Found, %: C 64.11; H 5.80; N 13.97.

*1-(2-Hydroxyphenyl)-4-(5,6-diphenyl-1,2,4-triazin-3-yl)pyrrolidine-2-one* (**20**)

A mixture of hydrazide **3** (2.35 g, 10 mmol), 1,2-diphenylethane-1,2-dione (2.10 g, 10 mmol), ammonium acetate (7.71 g, 100 mmol), and glacial acetic acid (40 mL) was heated at reflux for 24 h, then cooled down, and diluted with water (60 mL). The obtained oily mass was washed with hot water (2 × 60 mL), then poured with aqueous 5% hydrochloric acid solution (50 mL), and refluxed for 5 min. After cooling, the formed precipitate was filtered off, washed with water, and dried. To purify the solid, the crystalline product was dissolved in acetone (10 mL), and the solution was slowly poured into hexane (100 mL) in a thin stream. The formed solid was filtered off and washed with hexane. 

Light brown solid, yield 58.8%, m.p. 163–164 °C (acetone:hexane, 1:10).

^1^H NMR (DMSO-*d_6_*, 400 MHz), δ: 3.03 (d, *J* = 8.2 Hz, 2H, CH_2_CO), 4.15 (dd, *J* = 9.2, 5.4 Hz, 1H, NCH_2_), 4.25–4.38 (m, 1H, NCH_2_, 1H, CH), 6.83 (t, *J* = 7.6 Hz, 1H, H_arom_), 6.93 (d, *J* = 8.1 Hz, 1H, H_arom_), 7.14 (t, *J* = 7.8 Hz, 1H, H_arom_), 7.22 (d, *J* = 7.7 Hz, 1H, H_arom_), 7.37–7.55 (m, 10H, H_arom_), 9.63 (s, 1H, OH) ppm.

^13^C NMR (DMSO-*d_6_*, 101 MHz), δ: 35.96, 38.15 (CH_2_CO, CH), 53.22 (NCH_2_), 116.81, 119.13, 125.69, 128.20, 128.42, 128.53, 129.33, 129.47, 129.74, 130.68, 135.39, 135.46, 152.71, 155.87, 156.05, 167.07 (C_arom_), 172.51 (C=O) ppm.

IR (KBr), ν_max_: 1585 (C=N); 1656 (CO); 3067 (OH) cm^−1^.

Calcd. for C_25_H_20_N_4_O_2_, %: C 73.51; H 4.94; N 13.72. Found, %: C 73.44; H 5.02; N 13.68.

*General procedure for the preparation of benzimidazoles* **21**–**24a, b**.

A mixture of acid **1a** or **1b** (10 mmol), the corresponding *o*-phenylenediamine (30 mmol), and 6 N hydrochloric acid (30 mL) was refluxed for 24 h, then cooled down, and treated with aqueous 15% ammonium hydroxide solution to pH 8. The formed precipitate was filtered off, washed with water, and dried. The target benzimidazoles **21**–**24a, b** were recrystallized from propan-2-ol.

*4-(1H-benzo[d]imidazol-2-yl)-1-(2-hydroxyphenyl)pyrrolidine-2-one* (**21a**).

Dark yellow solid, yield 97.6%, m.p. 214–215 °C.

^1^H NMR (DMSO-*d_6_*, 400 MHz), δ: 2.62–3.08 (m, 2H, CH_2_CO), 3.77–4.28 (m, 3H, NCH_2_, CH), 6.84 (t, *J* = 7.5 Hz, 1H, H_arom_), 6.95 (d, *J* = 8.3 Hz, 1H, H_arom_), 7.15–7.22 (m, 4H, H_arom_), 7.50–7.58 (m, 2H, H_arom_), 10.49 (s, 1H, OH), 12.58 (s, 1H, NH) ppm.

^13^C NMR (DMSO-*d_6_*, 101 MHz), δ: 32.12, 36.67 (CH_2_CO, CH), 53.25 (NCH_2_), 114.73, 115.16, 116.97, 119.03, 121.89, 125.23, 128.48, 128.61, 153.37, 155.96 (C_arom_), 172.26 (C=O) ppm.

IR (KBr), ν_max_: 1598 (C=N); 1675 (CO); 2561–3175 (OH, NH) cm^−1^.

Calcd. for C_17_H_15_N_3_O_2_, %: C 69.61; H 5.15; N 14.33. Found, %: C 69.66; H 5.21; N 14.37.

*4-(1H-benzo[d]imidazol-2-yl)-1-(3,5-dichloro-2-hydroxyphenyl)pyrrolidin-2-one* (**21b**).

Light brown solid, yield 94.1%, m.p. 231–232 °C. 

^1^H NMR (DMSO-*d_6_*, 400 MHz), δ: 2.76–2.86 (m, 1H, CH_2_CO), 3.01–3.11 (m, 1H, CH_2_CO), 4.06–4.34 (m, 3H, NCH_2_, CH), 7.32–7.44 (m, 3H, H_arom_), 7.46–7.62 (m, 1H, H_arom_), 7.64–7.72 (m, 2H, H_arom_), 13.08 (s, 1H, NH) ppm.

^13^C NMR (DMSO-*d_6_*, 101 MHz), δ: 32.08, 36.27 (CH_2_CO, CH), 53.28 (NCH_2_), 114.25, 121.91, 122.27, 123.99, 127.34, 127.42, 128.59, 134.21, 140.01, 149.52, 155.36 (C_arom_), 172.06 (C=O) ppm.

IR (KBr), ν_max_: 1578 (C=N); 1680 (CO); 3065–3180 (OH, NH) cm^−1^.

Calcd. for C_17_H_13_Cl_2_N_3_O_2_, %: C 56.37; H 3.62; N 11.60. Found, %: C 56.32; H 3.67; N 11.55.

*4-(5-Methyl-1H-benzo[d]imidazol-2-yl)-1-(2-hydroxyphenyl)pyrrolidine-2-one* (**22a**).

White solid, yield 90.8%, m.p. 229–230 °C. 

^1^H NMR (DMSO-*d_6_*, 400 MHz), δ: 2.42 (s, 3H, CH_3_), 2.72 (dd, *J* = 16,4, 4.8 Hz, 1H, CH_2_CO), 3.00 (dd, *J* = 16.4, 8.0 Hz, 1H, CH_2_CO), 3.94–4.19 (m, 3H, NCH_2_, CH), 6.83 (t, *J* = 7.5 Hz, 1H, H_arom_), 6.95 (d, *J* = 8.3 Hz, 1H, H_arom_), 7.06 (d, *J* = 8.2 Hz, 1H, H_arom_), 7.11–7.25 (m, 2H, H_arom_), 7.36 (s, 1H, H_arom_), 7.45 (d, *J* = 8.1 Hz, 1H, H_arom_), 9.26 (br. s, 1H, OH), 11.36 (br. s, 1H, NH) ppm.

^13^C NMR (DMSO-*d_6_*, 101 MHz), δ: 21.23 (CH_3_), 31.91, 36.59 (CH_2_CO, CH), 53.13 (NCH_2_), 114.00, 114.46, 116.98, 119.01, 123.77, 125.16, 128.50, 128.63, 131.64, 135.60, 137.12, 153.41, 155.39 (C_arom_), 172.11 (C=O) ppm.

IR (KBr), ν_max_: 1597 (C=N); 1672 (CO); 2917–3121 (OH, NH) cm^−1^.

Calcd. for C_18_H_17_N_3_O_2_, %: C 70.34; H 5.58; N 13.67. Found, %: C 70.26; H 5.61; N 13.61.

*1-(3,5-Dichloro-2-hydroxyphenyl)-4-(5-methyl-1H-benzo[d]imidazol-2-yl)pyrrolidin-2-one* (**22b**).

Light yellow solid, yield 88.9%, m.p. 238–239 °C. 

^1^H NMR (DMSO-*d_6_*, 400 MHz), δ: 2.47 (s, 3H, CH_3_), 2.85–3.09 (m, 2H, CH_2_CO), 3.98–4.48 (m, 3H, NCH_2_, CH), 7.02–7.90 (m, 5H, H_arom_), 13.37 (s, 1H, NH) ppm.

^13^C NMR (DMSO-*d_6_*, 101 MHz), δ: 21.15 (CH_3_), 30.50, 35.99 (CH_2_CO, CH), 52.44 (NCH_2_), 113.50, 113.71, 122.01, 122.31, 126.14, 127.28, 127.48, 128.58, 131.03, 132.98, 134.49, 149.37, 154.39 (C_arom_), 171.89 (C=O) ppm.

IR (KBr), ν_max_: 1573 (C=N); 1684 (CO); 3055–3240 (OH, NH) cm^−1^.

Calcd. for C_18_H_15_Cl_2_N_3_O_2_, %: C 57.46; H 4.02; N 11.17. Found, %: C 57.52; H 3.98; N 11.19.

*4-(5-Chloro-1H-benzo[d]imidazol-2-yl)-1-(2-hydroxyphenyl)pyrrolidine-2-one* (**23a**).

Brown solid, yield 96.8%, m.p. 159–160 °C. 

^1^H NMR (DMSO-*d_6_*, 400 MHz), δ: 2.74–3.07 (m, 2H, CH_2_CO), 3.97–4.19 (m, 3H, NCH_2_, CH), 6.83 (t, *J* = 7.5 Hz, 1H, H_arom_), 6.95 (d, *J* = 8.0 Hz, 1H, H_arom_), 7.13–7.20 (m, 2H, H_arom_), 7.23–7.29 (m, 1H, H_arom_), 7.59 (d, *J* = 8.6 Hz, 1H, H_arom_), 7.64 (s, 1H, H_arom_), 9.39 (br. s, 1H, OH) ppm.

^13^C NMR (DMSO-*d_6_*, 101 MHz), δ: 31.79, 36.15 (CH_2_CO, CH), 52.98 (NCH_2_), 114.47, 115.81, 116.88, 119.09, 122.61, 124.92, 125.25, 126.72, 128.41, 128.51, 135.94, 138.32, 152.79, 153.10, 156.97 (C_arom_), 172.05 (C=O) ppm.

IR (KBr), ν_max_: 1600 (C=N); 1675 (CO); 2564–3098 (OH, NH) cm^−1^.

Calcd. for C_17_H_14_ClN_3_O_2_, %: C 62.30; H 4.31; N 10.82. Found, %: C 62.22; H 4.40; N 10.90.

*4-(5-Chloro-1H-benzo[d]imidazol-2-yl)-1-(3,5-dichloro-2-hydroxyphenyl)pyrrolidin-2-one* (**23b**).

Light grey solid, yield 93.4%, m.p. 191–192 °C. 

^1^H NMR (DMSO-*d_6_*, 400 MHz), δ: 2.98–3.02 (m, 2H, CH_2_CO), 4.12–4.18 (m, 2H, NCH_2_), 4.40–4.48 (m, 1H, CH), 7.22 (s, 1H, H_arom_), 7.40–7.43 (m, 1H, H_arom_), 7.45–7.50 (m, 2H, H_arom_), 7.71–7.78 (m, 1H, H_arom_), 9.25 (br. s, 1H, OH), 12.47 (br. s, 1H, NH) ppm.

^13^C NMR (DMSO-*d_6_*, 101 MHz), δ: 30.45, 35.80 (CH_2_CO, CH), 52.41 (NCH_2_), 113.87, 115.60, 122.14, 122.33, 125.01, 127.26, 126.56, 128.58, 138.64, 140.15, 149.23, 156.11 (C_arom_), 171.87 (C=O) ppm.

IR (KBr), ν_max_: 1606 (C=N); 1702 (CO); 3054–3298 (OH, NH) cm^−1^.

Calcd. for C_17_H_12_Cl_3_N_3_O_2_, %: C 51.48; H 3.05; N 10.59. Found, %: C 51.51; H 3.09; N 10.62.

*4-(5-Fluoro-1H-benzo[d]imidazol-2-yl)-1-(2-hydroxyphenyl)pyrrolidine-2-one* (**24a**).

Grey solid, yield 87%, m.p. 159–160 °C. 

^1^H NMR (DMSO-*d_6_*, 400 MHz), δ: 2.73–2.77 (m, 1H, CH_2_CO), 2.82–3.00 (m, 1H, CH_2_CO), 3.98–4.16 (m, 3H, NCH_2_, CH), 6.83 (t, *J* = 7.5 Hz, 1H, H_arom_), 6.91–7.22 (m, 4H, H_arom_), 7.34 (s, 1H, H_arom_), 7.46–7.62 (m, 1H, H_arom_), 10.16, 10.19 (2s, 1H, OH), 12.72 (s, 1H, NH) ppm.

^13^C NMR (DMSO-*d_6_*, 101 MHz), δ: 32.09, 36.41 (CH_2_CO, CH), 53.18 (NCH_2_), 109.58, 111.91, 112.03, 116.91, 119.09, 125.31, 128.40, 128.52, 153.16, 156.80, 157.67, 158.64 (C_arom_), 172.26 (C=O) ppm.

IR (KBr), ν_max_: 1600 (C=N); 1675 (CO); 2564–3098 (OH, NH) cm^−1^.

Calcd. for C_17_H_14_FN_3_O_2_, %: C 65.59; H 4.53; N 13.50. Found, %: C 65.64; H 4.54; N 13.46.

*1-(3,5-Dichloro-2-hydroxyphenyl)-4-(5-fluoro-1H-benzo[d]imidazol-2-yl)pyrrolidin-2-one* (**24b**).

Dark yellow solid, yield 89.5%, m.p. 183–184 °C. 

^1^H NMR (DMSO-*d_6_*, 400 MHz), δ: 2.57–2.68 (m, 1H, CH_2_CO), 2.98–3.08 (m, 1H, CH_2_CO), 3.93–4.20 (m, 3H, NCH_2_, CH), 7.01–7.15 (m, 1H, H_arom_), 7.24–7.47 (m, 2H, H_arom_), 7.48–7.64 (m, 2H, H_arom_), 12.40 (s, 1H, NH) ppm.

^13^C NMR (DMSO-*d_6_*, 101 MHz), δ: 32.32, 36.78 (CH_2_CO, CH), 52.96 (NCH_2_), 110.08, 110.34, 121.84, 122.18, 127.40, 127.46, 128.54, 149.61, 157.67, 158.59 (d, *J_C–F_* = 235.3 Hz) (C_arom_), 172.54 (C=O) ppm.

IR (KBr), ν_max_: 1601 (C=N); 1705 (CO); 3051–3290 (OH, NH) cm^−1^.

HRMS (ESI) for C_17_H_12_Cl_2_FN_3_O_2_ + H^+^, calcd. 380.0369, found 380.0365 [M + H^+^].

Calcd. for C_17_H_12_Cl_2_FN_3_O_2_, %: C 53.71; H 3.18; N 5.00. Found, %: C 53.66; H 3.15; N 4.96.

*General method for the preparation of butanoic acids* **25**–**27a, b***and* **28b**.

A mixture of the corresponding benzimidazole **21a, b**–**23a, b,** and **24b** (2 mmol) and aqueous 20% NaOH solution (10 mL) was heated at reflux for 4 h, then cooled down, and acidified with diluted acetic acid to pH 6. The form precipitate was filtered off, washed with water, and dried to give the title compounds **25**–**27a, b,** and **28b**. The products were purified by dissolving them in aqueous 2% sodium hydroxide solution and filtering and acidifying the filtrate with diluted acetic acid to pH 6–7.

*3-(1H-benzo[d]imidazol-2-yl)-4-((2-hydroxyphenyl)amino)butanoic acid* (**25a**).

Dark brown solid, yield 92%, m.p. 185 °C (decomp.).

^1^H NMR (DMSO-*d_6_*, 400 MHz), δ: 2.72–3.02 (m, 2H, CH_2_CO), 3.26–3.76 (m, 3H, NHCH_2_, CH), 6.28–6.75 (m, 2H, H_arom_), 7.09–7.17 (m, 4H, H_arom_), 7.48–7.58 (m, 2H, H_arom_), 11.70 (br. s, 3H, NH, 2OH) ppm.

^13^C NMR (DMSO-*d_6_*, 101 MHz), δ: 35.66, 36.88 (CH_2_CO, CH), 46.60 (NHCH_2_), 109.79, 113.49, 114.68, 115.96, 117.00, 118.98, 119.72, 121.19, 121.84, 128.49, 128.61, 136.99, 144.08, 153.46, 156.43 (C_arom_), 173.55 (C=O) ppm.

IR (KBr), ν_max_: 1579 (C=N); 1673 (CO); 2927–3369 (OH, NH) cm^−1^.

Calcd. for C_17_H_17_N_3_O_3_, %: C 65.58; H 5.50; N 13.50. Found, %: C 65.53; H 5.54; N 13.56.

*3-(1H-benzo[d]imidazol-2-yl)-4-((3,5-dichloro-2-hydroxyphenyl)amino)butanoic acid* (**25b**).

Brown solid, yield 92.9%, m.p. 199 °C (decomp.).

^1^H NMR (DMSO-*d_6_*, 400 MHz), δ: 2.71–2.99 (m, 2H, CH_2_CO), 3.26–3.74 (m, 3H, NHCH_2_, CH), 5.62 (s, 1H, NH), 6.25–6.75 (m, 2H, H_arom_), 7.09–7.18 (m, 2H, H_arom_), 7.49–7.53 (m, 2H, H_arom_), 12.30 (br. s, 2H, NH, OH) ppm.

^13^C NMR (DMSO-*d_6_*, 101 MHz), δ: 35.01, 35.78 (CH_2_CO, CH), 46.21 (NHCH_2_), 107.78, 114.56, 120.46, 121.33, 124.76, 138.47, 140.63, 155.70 (C_arom_), 173.14 (C=O) ppm.

IR (KBr), ν_max_: 1607 (C=N); 1711 (CO); 3062–3298 (OH, NH) cm^−1^.

Calcd. for C_17_H_15_Cl_2_N_3_O_3_, %: C 53.70; H 3.98; N 11.05. Found, %: C 53.78; H 4.01; N 11.06.

*4-((2-Hydroxyphenyl)amino)-3-(5-methyl-1H-benzo[d]imidazol-2-yl)butanoic acid* (**26a**).

Light orange solid, yield 96.4%, m.p. 160 °C (decomp.).

^1^H NMR (DMSO-*d_6_*, 400 MHz), δ: 2.39, 2.41 (2s, 3H, CH_3_), 2.64–3.02 (m, 2H, CH_2_CO), 3.19–3.75 (m, 3H, NHCH_2_, CH), 4.83 (br. s, 1H, NH), 6.30–7.57 (m, 7H, H_arom_), 9.22 (br. s, 1H, OH), 12.27 (br. s, 2H, NH) ppm.

^13^C NMR (DMSO-*d_6_*, 101 MHz), δ: 21.28 (CH_3_), 35.41, 36.91 (CH_2_CO, CH), 46.63 (NHCH_2_), 109.77, 113.49, 116.02, 117.02, 119.00, 119.73, 122.62, 123.28, 125.19, 128.52, 128.65, 130.33, 136.92, 144.06, 153.49, 155.61 (C_arom_), 173.17 (C=O) ppm.

IR (KBr), ν_max_: 1579 (C=N); 1673 (CO); 2920–3147 (OH, NH) cm^−1^.

Calcd. for C_18_H_19_N_3_O_3_, %: C 66.45; H 5.89; N 12.91. Found, %: C 66.39; H 5.83; N 12.97.

*4-((3,5-Dichloro-2-hydroxyphenyl)amino)-3-(5-methyl-1H-benzo[d]imidazol-2-yl)butanoic acid* (**26b**).

Grey solid, yield 93.7%, m.p. 177 °C (decomp.).

^1^H NMR (DMSO-*d_6_*, 400 MHz), δ: 2.37 (s, 3H, CH_3_), 2.50–2.59 (m, 2H, CH_2_CO), 3.19–3.55 (m, 2H, NHCH_2_), 3.56–3.76 (m, 1H, CH), 5.92 (br. s, 1H, NH), 6.24–6.70 (m, 2H, H_arom_), 6.90, 7.33 (2d, 2H, *J* = 8.5 Hz, H_arom_), 7.24 (s, 1H, H_arom_), 9.55 (br. s, 2H, NH, OH) ppm.

^13^C NMR (DMSO-*d_6_*, 101 MHz), δ: 23.19 (CH_3_), 33.43, 36.14 (CH_2_CO, CH), 46.84 (NHCH_2_), 107.08, 114.45, 119.86, 122.25, 129.83, 141.57, 157.20 (C_arom_), 173.97 (C=O) ppm.

IR (KBr), ν_max_: 1579 (C=N); 1689 (CO); 3104–3378(OH, NH) cm^−1^.

Calcd. for C_18_H_18_Cl_2_FN_3_O_3_, %: C 54.84; H 4.35; N 10.66. Found, %: C 54.89; H 4.30; N 10.70.

*3-(5-Chloro-1H-benzo[d]imidazol-2-yl)-4-((2-hydroxyphenyl)amino)butanoic acid* (**27a**).

Brown solid, yield 89.9%, m.p. 169 °C (decomp.).

^1^H NMR (DMSO-*d_6_*, 400 MHz), δ: 2.72–3.01 (m, 2H, CH_2_CO), 3.25–3.49 (m, 3H, NHCH_2_), 3.52–3.74 (m, 1H, CH), 4.85 (s, 1H, NH), 6.24–6.70 (m, 3H, H_arom_), 6.95–7.25 (m, 2H, H_arom_), 7.49–77.52 (m, 2H, H_arom_), 9.20, 12,41 (2br. s, 2H, NH, OH) ppm.

^13^C NMR (DMSO-*d_6_*, 101 MHz), δ: 35.47, 35.81 (CH_2_CO, CH), 46.59 (NHCH_2_), 109.75, 113.49, 116.04, 119.09, 119.70, 121.22, 121.44, 121.46, 125.64, 128.37, 128.49, 131.19, 136.82, 144.04, 153.09, 157.66 (C_arom_), 173.08 (C=O) ppm.

IR (KBr), ν_max_: 1576 (C=N); 1681 (CO); 3069–3360 (OH, NH) cm^−1^.

Calcd. for C_17_H_16_ClN_3_O_3_, %: C 59.05; H 4.66; N 12.15. Found, %: C 59.10; H 4.62; N 12.09.

*3-(5-Chloro-1H-benzo[d]imidazol-2-yl)-4-((3,5-dichloro-2-hydroxyphenyl)amino)butanoic acid* (**27b**).

Pale grey solid, yield 92.5%, m.p. 187 °C (decomp.).

^1^H NMR (DMSO-*d_6_*, 400 MHz), δ: 2.52–2.71 (m, 2H, CH_2_CO), 3.32–3.53 (m, 3H, NHCH_2_), 3.61–3.74 (m, 1H, CH), 5.86 (br. s, 1H, NH), 6.50 (s, 2H, H_arom_), 7.10, 7.45 (2d, 2H, *J* = 8.5 Hz, Harom), 7.52 (s, 1H, H_arom_), 10.47 (br. s, 3H, NH, 2OH) ppm.

^13^C NMR (DMSO-*d_6_*, 101 MHz), δ: 36.16, 39.00 (CH_2_CO, CH), 46.62 (NHCH_2_), 107.36, 114.50, 115.48, 120.26, 121.10, 122.74, 125.32, 140.76, 141.25 158.97 (C_arom_), 173.158 (C=O) ppm.

IR (KBr), ν_max_: 1589 (C=N); 1706 (CO); 3097–3391 (OH, NH) cm^−1^.

Calcd. for C_17_H_14_Cl_3_N_3_O_3_, %: C 49.24; H 3.40; N 10.13. Found, %: C 49.19; H 3.35; N 10.17.

*4-((3,5-Dichloro-2-hydroxyphenyl)amino)-3-(5-fluoro-1H-benzo[d]imidazol-2-yl)butanoic acid* (**28b**).

Light brown solid, yield 94.3%, m.p. 182 °C (decomp.).

^1^H NMR (DMSO-*d_6_*, 400 MHz), δ: 2.59–2.97 (m, 2H, CH_2_CO), 3.27–3.75 (m, 3H, NHCH_2_, CH), 5.62 (s, 1H, NH), 6.58–6.63 (m, 1H, H_arom_), 7.11–7.28 (m, 2H, H_arom_), 7.43–7.09 (m, 2H, H_arom_),11.90 (br. s, 3H, NH, 2OH) ppm.

^13^C NMR (DMSO-*d_6_*, 101 MHz), δ: 35.10, 35.67 (CH_2_CO, CH), 46.19 (NHCH_2_), 107.74, 109.11, 109.36, 114.54, 120.45, 122.24, 124.75, 127.40, 127.49, 128.52, 138.44, 140.60, 157.11, 157.75, 158.28 (d, *J_C-F_* = 234.1 Hz) (C_arom_), 173.11 (C=O) ppm.

IR (KBr), ν_max_: 1587 (C=N); 1704 (CO); 3088–3385 (OH, NH) cm^−1^.

HRMS (ESI) for C_17_H_14_Cl_2_FN_3_O_3_ + H^+^, calcd. 398.0474, found 398.0472 [M + H^+^].

Calcd. for C_17_H_14_Cl_2_FN_3_O_3_, %: C 51.28; H 3.54; N 10.55. Found, %: C 51.23; H 3.52; N 10.57.

### 3.2. Bacterial Strains and Culture Conditions

The multidrug-resistant and genetically defined isolates were obtained from the ARisolate bank at Centre for Disease Control (CDC, United States). *S. aureus* TCH 1516 (USA300) was obtained from the American Type Culture Collection and maintained as a laboratory strain [39,40] that was used for in vitro and in vivo pharmacological screening. Prior to the study, all strains were maintained in commercial cryopreservation systems at −80 °C. Bacterial strains were subcultured on Columbia Sheep Blood agar or Tryptic-Soy agar (Becton Dickenson, Franklin Lakes, NJ, USA), while fungi were propagated on Potato-Dextrose agar (Becton Dickenson, Franklin Lakes, NJ, USA). Unless otherwise specified, all antibacterial susceptibility studies were performed in Cation-Adjusted Mueller–Hinton broth (CAMBH) for liquid cultures (Liofilchem, Via Szia, Italy) or MOPS/RPMI media for fungal assays (Becton Dickenson, Franklin Lakes, NJ, USA).

### 3.3. Minimal Inhibitory Concentration Determination

The minimal inhibitory concentrations (MICs) of compounds **1a**–**28b**, as well as various antibiotics, were determined according to the recommendations of the Clinical and Laboratory Standards Institute (CLSI) [41]. The MICs for the compounds and comparator antibiotics were determined according to the testing standard broth microdilution methods described in CLSI document M07-A8 against the libraries of Gram-positive and Gram-negative pathogens, as well as pathogenic fungi. The compounds and antibiotics were dissolved in dimethyl sulfoxide (DMSO) to achieve a final concentration of 30 mg/mL. Series of dilutions were prepared in deep 96-well microplates to achieve 2× assay concentrations (0.5–128 µg/mL) and were then transferred to the assay plates. A standardized inoculum was prepared using a direct colony suspension. Within 15 min of preparation, the adjusted inoculum suspension was diluted in sterile CAMBH to achieve final concentrations of approximately 5 × 10^5^ CFU/mL (range, 2 × 10^5^ to 8 × 10^5^ CFU/mL) in each well. The inoculum was transferred to the assay plates to achieve a 1× assay concentration. 

For the anaerobic pathogens (*C*. *difficile*), the inoculum was prepared by using anaerobic Sheep Blood agar, and plates were incubated in an anaerobic chamber for 48 h. The inoculum was prepared as described elsewhere, and the plates containing investigational compounds were further incubated in an anaerobic chamber for 24–48 h [42]. Inoculated microdilution plates were incubated at 35 °C for 16 to 20 h in an ambient-air incubator within 15 min of the addition of the inoculum.

### 3.4. Cell Lines and Culture Conditions

The non-cancerous HSAEC1-KT cells (CRL-4050) were kindly provided by Dr. Arryn Craney (Orlando Health, Orlando, FL, USA) and were maintained in complete SAGM culture media (Lonza, Moristown, NJ, USA) containing growth supplements. The non-small cell human lung carcinoma A549 cells were obtained from the American Type Culture Collection (Rockville, MD, USA). Cells were maintained in Dulbecco’s Modified Eagle Medium/Nutrient Mixture F-12 (DMEM/F-12) (Gibco, Waltham, MA, USA) supplemented with 10% fetal bovine serum (10% FBS) (Gibco, Waltham, MA, USA) and 100 U/mL penicillin and 100 μg/mL streptomycin (P/S). Cells were cultured at 37 °C in a humidified atmosphere containing 5% CO_2_. Cells were fed every 2–3 days (for A549) or every 3 days (HSAEC1-KT) and subcultured upon reaching 70–80% confluence.

### 3.5. In Vitro Cytotoxic Activity Determination

The viability of A549 and HSAEC1-KT cells after the treatment with compounds or cisplatin, which served as the cytotoxicity control, was evaluated by using a commercial MTT (3-[4,5-methylthiazol-2-yl]-2,5-diphenyl-tetrazolium bromide) assay (ThermoFisher Scientific, Waltham, MA, USA). Briefly, cells were plated in the flat-bottomed 96-well microplates (1 × 10^4^ cells/well) and incubated overnight to facilitate the attachment. The test compounds were dissolved in hybridoma-grade DMSO (Sigma-Aldrich, St. Louis, MO, USA) and then further serially diluted in cell culture media containing 0.25% DMSO to achieve 100 µM for each compound. 

Subsequently, the media from the cells were aspirated, and the compounds were added to the microplates. The cells were incubated at 37 °C, with 5% CO_2_, for 24 h. After incubation, 10 μL of Vybrant^®^ MTT Cell Proliferation Reagent (ThermoFisher Scientific) was added, and cells were further incubated for 4 h. After incubation, the media were aspirated, and the resulting formazan was solubilized through the addition of 100 μL of DMSO. The absorbance was then measured at 570 nm using a microplate reader (Multiscan, ThermoFisher Scientific). The following formula was used to calculate the % of A549 viability: ([AE − AB]/[AC − AB]) × 100%. AE, AC, and AB were defined as the absorbance of experimental samples, untreated samples, and blank controls, respectively. The experiments were performed in triplicate.

### 3.6. Statistical Analysis

The results are expressed as the mean ± standard deviation (SD). Statistical analyses were performed with Prism (GraphPad Software, version 9, San Diego, CA, USA), using the Kruskal–Wallis test and two-way ANOVA. *p* < 0.05 was accepted as significant.

## 4. Conclusions

In the present study, a series of 1-(2-hydroxyphenyl)- and (3,5-dichloro-2-hydroxyphenyl)-5-oxopyrrolidine-3-carboxylic acids derivatives was synthesized, characterized, and evaluated for their antimicrobial activity using representative multidrug-resistant bacterial pathogens with emerging and genetically defined resistance mechanisms. In addition to that, the in vitro cytotoxic properties were characterized using A549 human lung cell culture models. 

The results revealed that the 1-(2-hydroxyphenyl)-5-oxopyrrolidine-3-carboxylic acid derivatives showed selective, Gram-positive bacteria-directed antimicrobial activity. The incorporation of a 2-thienyl fragment in the hydrazone structure significantly enhanced antibacterial activity against methicillin-resistant *S. aureus* TCH 1516 (USA 300 lineage) (MIC 16 µg/L) and *C. difficile* AR-1067 (MIC 32 µg/mL), although no activity was observed against Gram-negative or fungal pathogens (MIC > 128 µg/mL). This suggests that the 2-thienyl fragment plays an important role in the mechanism of action of these compounds against Gram-positive pathogens. A dechlorinated derivative with a 5-fluorobenzimidazole moiety resulted in an increase in the antimicrobial activity spectrum. A compound containing a 5-fluorobenzimidazole moiety showed 1-fold higher antimicrobial activity against *S. aureus* TCH 1516 (MIC of 8 µg/mL), as well as Gram-negative pathogens, except for *A. baumannii*. Interestingly, the replacement of 2-thienyl with a 5-nitro-2-thienyl fragment in hydrazone strongly increased the antifungal activity of the compounds against drug-resistant *Candida* and *Aspergillus* isolates. These results demonstrated that 1-(2-hydroxyphenyl)- and (3,5-dichloro-2-hydroxyphenyl-5-oxopyrrolidine-3-carboxylic acid derivatives could be further explored as a promising scaffold for the discovery of antimicrobial candidates targeting multidrug-resistant Gram-positive pathogens and drug-resistant fungi. Further studies are needed to better understand the cellular targets, pharmacological properties, and safety of these compounds.

Finally, the in vitro anticancer activity characterization showed that compounds demonstrated structure-depended anticancer activity against A549 cells. Among all tested compounds, 1-(3,5-dichloro-2-hydroxyphenyl)-4-(5-fluoro-1H-benzo[d]imidazol-2-yl)pyrrolidin-2-one showed the highest cytotoxic properties, making it as an attractive candidate for further anticancer compound development. 

## Figures and Tables

**Table 1:** The in vitro antimicrobial activity of novel 1,3-disubstituted 5-oxopyrrolidines **1a**–**28b** bearing hydrazone, azole, and azine moieties against representative multidrug-resistant bacterial pathogens with genetically defined resistance mechanisms. The antimicrobial activity is expressed as the average of the minimal inhibitory concentration (MIC) for each compound or control antibiotics determined from triplicate experiments.

Compound	Minimal Inhibitory Concentration (MIC, µg/mL)				
	*S. aureus* TCH 1516	*A. baumannii* AR-0033	*K. pneumoniae* AR-0049	*P. aeruginosa* AR-0064	*C. difficile* AR-1067
**1a**	>128	>128	>128	>128	>128
**1b**	>128	>128	>128	>128	>128
**2a**	>128	>128	>128	>128	>128
**2b**	64	128	>128	>128	>128
**3**	>128	>128	>128	>128	>128
**4**	>128	>128	>128	>128	>128
**5**	>128	>128	>128	>128	>128
**6**	>128	>128	>128	>128	>128
**7**	64	>128	>128	>128	16
**8**	64	>128	>128	>128	>128
**9**	128	>128	>128	>128	>128
**10**	>128	>128	>128	>128	>128
**11**	>128	>128	>128	>128	>128
**12**	>128	>128	>128	>128	>128
**13**	>128	>128	>128	>128	>128
**14**	16	>128	>128	>128	32
**15**	>128	>128	>128	>128	>128
**16**	>128	>128	>128	>128	>128
**17**	>128	>128	>128	>128	>128
**18**	>128	>128	>128	>128	>128
**19**	>128	>128	>128	>128	>128
**20**	32	>128	>128	128	128
**21a**	>128	>128	>128	>128	>128
**21b**	32	>128	>128	>128	64
**22a**	>128	>128	>128	>128	>128
**22b**	64	>128	>128	>128	>128
**23a**	128	>128	>128	>128	>128
**23b**	64	>128	>128	>128	>128
**24a**	>128	>128	>128	>128	>128
**24b**	8	>128	128	64	128
**25a**	>128	>128	>128	>128	>128
**25b**	128	>128	>128	>128	128
**26a**	>128	>128	>128	>128	>128
**26b**	>128	>128	>128	>128	>128
**27a**	>128	>128	>128	>128	>128
**27b**	>128	>128	>128	>128	>128
**28b**	>128	>128	>128	>128	>128
Clindamycin	32	N/A	N/A	N/A	32
Metronidazole	N/A	N/A	N/A	N/A	2
Vancomycin	2	N/A	N/A	N/A	2
Meropenem	N/A	32	16	16	N/A

**Table 2:** The in vitro antimicrobial activity of novel 1,3-disubstituted 5-oxopyrrolidines **1a**–**28b** bearing hydrazone, azole, and azine moieties against representative multidrug-resistant fungal pathogens. The antimicrobial activity is expressed as the average of the minimal inhibitory concentration (MIC) for each compound or control antibiotics determined from triplicate experiments.

Compounds	Minimal Inhibitory Concentration (MIC, µg/mL)					
	*C. auris* AR-381	*C. auris* AR-382	*C. auris* AR-383	*A. fumigatus* AR-731	*A. fumigatus* AR-732	*A. fumigatus* AR-733
**1a**	>128	>128	>128	>128	>128	>128
**1b**	>128	>128	>128	>128	>128	>128
**2a**	>128	>128	>128	>128	>128	>128
**2b**	>128	>128	>128	>128	>128	>128
**3**	>128	>128	>128	>128	>128	>128
**4**	>128	>128	>128	>128	>128	>128
**5**	>128	>128	>128	>128	>128	>128
**6**	>128	>128	>128	>128	>128	>128
**7**	>128	>128	>128	>128	>128	>128
**8**	>128	>128	>128	>128	>128	>128
**9**	>128	>128	>128	>128	>128	>128
**10**	>128	>128	>128	>128	>128	>128
**11**	>128	>128	>128	>128	>128	>128
**12**	>128	>128	>128	>128	>128	>128
**13**	>128	>128	>128	>128	>128	>128
**14**	>128	>128	>128	>128	>128	>128
**15**	16	16	16	32	64	32
**16**	>128	>128	>128	>128	>128	>128
**17**	>128	>128	>128	>128	>128	>128
**18**	>128	>128	>128	>128	>128	>128
**19**	>128	>128	>128	>128	>128	>128
**20**	>128	>128	>128	>128	>128	>128
**21a**	>128	>128	>128	>128	>128	>128
**21b**	>128	>128	>128	>128	>128	>128
**22a**	>128	128	128	>128	>128	>128
**22b**	>128	>128	>128	>128	>128	>128
**23a**	>128	>128	>128	>128	>128	>128
**23b**	>128	>128	>128	>128	>128	>128
**24a**	>128	>128	>128	>128	>128	>128
**24b**	>128	>128	>128	>128	>128	>128
**25a**	>128	>128	>128	>128	>128	>128
**25b**	>128	>128	>128	>128	>128	>128
**26a**	>128	>128	>128	>128	>128	>128
**26b**	128	>128	>128	>128	>128	>128
**27a**	>128	>128	>128	>128	>128	>128
**27b**	128	>128	>128	>128	>128	>128
**28b**	>128	>128	>128	>128	>128	>128
Fluconazole	8	16	128	N/A	N/A	N/A
Flucytosine	2	<0.5	<0.5	N/A	N/A	N/A
Voriconazole	<0.5	<0.5	<0.5	2	1	2

**Table 3:** The antimicrobial activity of compounds **14** and **24b** against multidrug resistant *S. aureus* strains with a vancomycin-intermediate-resistance phenotype. The antimicrobial activity is expressed as the average of minimal inhibitory concentration (MIC) for the selected compounds or control antibiotics determined from triplicate experiments.

Bacterial Strain	Resistance Mechanisms	Antimicrobial Activity (MIC, µg/mL)			
		14	24b	VAN	DAP
***S. aureus* AR-215**	*aph-STPH*, *DHA1*, *erm(A)*, *mecA*, *spc*, *tet(38)*	16	8	4	2
***S. aureus* AR-216**	*aph(3*′*)-III*, *aph-STPH*, *fosB*, *mecA*, *mph(C)*, *sat-4A*	8	8	8	4
***S. aureus* AR-217**	*aph-STPH*, *blaI*, *dfrG*, *fosB*, *mecA*, *Z*	16	4	4	2
***S. aureus* AR-218**	*aph(3*′*)-III*, *aph-STPH*, *DHA1*, *erm(A)*, *mecA*, *norA*, *spc*, *tet(38)*, *tet(K)*	8	4	4	2
***S. aureus* AR-219**	*aac(6*′*)-aph(2*″*)*, *aadD*, *aph-STPH*, *DHA1*, *erm(A)*, *mecA*, *norA*, *spc*, *tet(38)*, *tet(K)*	4	2	8	4
***S. aureus* AR-220**	*aph-STPH*, *DHA1*, *erm(A)*, *mecA*, *norA*, *spc*, *tet(38)*	16	8	4	2

Abbreviations: VAN—vancomycin, DAP—daptomycin.

## Data Availability

Not applicable.

## Supplementary Materials

The following supporting information can be downloaded at: https://www.mdpi.com/article/10.3390/ijms24097966/s1.

## References

[1] Vazquez-Guillamet C., Kollef M.H. (2014). Treatment of gram—Positive infections in critically ill patients. BMC Infect. Dis..

[2] Stogios P.J., Savchenko A. (2020). Molecular mechanisms of vancomycin resistance. Protein Sci..

[3] Leclercq R., Derlot E., Duval J., Courvalin P. (1988). Plasmid-mediated resistance to vancomycin and teicoplanin in *Enterococcus faecium*. N. Engl. J. Med..

[4] Ealy V.L., Lessard I.A., Roper D.I., Knox J.R., Walsh C.T. (2000). Vancomycin resistance in enterococci: Reprogramming of the D-ala-D-ala ligases in bacterial peptidoglycan biosynthesis. Chem. Biol..

[5] Périchon B., Courvalin P. (2011). Glycopeptide Resistance Antibiotic Discovery and Development.

[6] Arastehfar A., Gabaldón T., Garcia-Rubio R., Jenks J.D., Hoenigl M., Salzer H.J.F., Ilkit M., Lass-Flörl C., Perlin D.S. (2020). Drug-Resistant Fungi: An Emerging Challenge Threatening Our Limited Antifungal Armamentarium. Antibiotics.

[7] Brown G.D., Denning D.W., Gow N.A.R., Levitz S.M., Netea M.G., White T.C. (2013). Hidden killers: Human fungal infections. Sci. Transl. Med..

[8] Jeanvoine A., Rocchi S., Bellanger A.P., Reboux G., Millon L. (2020). Azole-resistant Aspergillus fumigatus: A global phenomenon originating in the environment?. Med. Mal. Infect..

[9] Mohammadi F., Hashemi S.J., Zoll J., Melchers W.J., Rafati H., Dehghan P., Rezaie S., Tolooe A., Tamadon Y., van der Lee H.A. (2015). Quantitative Analysis of Single-Nucleotide Polymorphism for Rapid Detection of TR34/L98H- and TR46/Y121F/T289A-Positive Aspergillus fumigatus Isolates Obtained from Patients in Iran from 2010 to 2014. Antimicrob. Agents Chemother..

[10] Benhamou R.I., Bibi M., Steinbuch K.B., Engel H., Levin M., Roichman Y., Berman J., Fridman M. (2017). Real-Time Imaging of the Azole-Class of Antifungal Drugs in Live Candida Cells ACS Chemical Biology. ACS Chem. Biol..

[11] Campoy S., Adrio J.L. (2017). Antifungals. Biochem. Pharm..

[12] Zhou C.-H., Wang Y. (2012). Recent researches in triazole compounds as medicinal drugs. Curr. Med. Chem..

[13] Zhang H.-Z., Gan L.-L., Wang H., Zhou C.-H. (2017). New Progress in Azole Compounds as Antimicrobial Agents. Mini Rev. Med. Chem..

[14] Haroon M., Akhtar T., Khalid M., Ali S., Zahra S., Haq I.U., Alhujaily M., de B Dias M.C.H., Leite A.C.L., Muhammad S. (2021). Synthesis, antioxidant, antimicrobial and antiviral docking studies of ethyl 2-(2-(arylidene)hydrazinyl)thiazole-4-carboxylates. Z. Naturforsch. C J. Biosci..

[15] Tiperciuc B., Pârvu A., Tamaian R., Nastasă C., Ionuţ I., Oniga O. (2013). New anti-inflammatory thiazolyl-carbonyl-thiosemicarbazides and thiazolyl-azoles with antioxidant properties as potential iNOS inhibitors. Arch. Pharm. Res..

[16] Aisha, Raza M.A., Farwa U., Rashid U., Maurin J.K., Budzianowski A. (2022). Synthesis, Single Crystal, *In-Silico* and *In-Vitro* Assessment of the Thiazolidinones. J. Mol. Struct..

[17] Eze C.C., Ezeokonkwo A.M., Ugwu I.D., Eze U.F., Onyeyilim E.L., Attah I.S., Okonkwo I.V. (2022). Azole-Pyrimidine Hybrid Anticancer Agents: A Review of Molecular Structure, Structure Activity Relationship, and Molecular Docking. Anticancer Agents Med. Chem..

[18] Ahmad K., Khan M.K.A., Baig M.H., Imran M., Gupta G.K. (2018). Role of azoles in cancer prevention and treatment: Present and future perspectives. Anti-Cancer Agents Med. Chem..

[19] Sari S., Sabuncuoğlu S., Koçak Aslan E., Avci A., Kart D., Özdemir Z., Acar M.F., Sayoğlu B., Alagöz M.A., Karakurt A. (2022). Azoles containing naphthalene with activity against Gram-positive bacteria: *In vitro* studies and *in silico* predictions for flavohemoglobin inhibition. J. Biomol. Struct. Dyn..

[20] Bello-Vieda N.J., Pastrana H.F., Garavito M.F., Ávila A.G., Celis A.M., Muñoz-Castro A., Restrepo S., Hurtado J.J. (2018). Antibacterial Activities of Azole Complexes Combined with Silver Nanoparticles. Molecules.

[21] Vasava M.S., Bhoi M.N., Rathwa S.K., Jethava D.J., Acharya P.T., Patel D.B., Patel H.D. (2020). Benzimidazole: A Milestone in the Field of Medicinal Chemistry, *Mini Rev*. Med. Chem..

[22] Khalifa M., Gobouri A., Kabli F., Altalhi T., Almalki A., Mohamed M. (2018). Synthesis, antibacterial, and anti HepG2 cell line human hepatocyte carcinoma activity of some new potentially benzimidazole-5-(aryldiazenyl)thiazole derivatives. Molecules.

[23] Cheong J.E., Zaffagni M., Chung I., Xu Y., Wang Y., Jernigan F.E., Zetter B.R., Sun L. (2018). Synthesis and anticancer activity of novel water soluble benzimidazole carbamates. Eur. J. Med. Chem..

[24] Baldisserotto A., Demurtas M., Lampronti I., Tacchini M., Moi D., Balboni G., Vertuani S., Manfredini S., Onnis V. (2020). In-Vitro Evaluation of Antioxidant, Antiproliferative and Photo-Protective Activities of Benzimidazolehydrazone Derivatives. Pharmaceuticals.

[25] Law C.S.W., Yeong K.Y. (2021). Benzimidazoles in Drug Discovery: A Patent Review. ChemMedChem.

[26] Vitaku E., Smith D.T., Njardarson J.T. (2014). Analysis of the structural diversity, substitution patterns, and frequency of nitrogen heterocycles among U.S. FDA approved pharmaceuticals. J. Med. Chem..

[27] Sapijanskaitė-Banevič B., Palskys V., Vaickelionienė R., Šiugždaitė J., Kavaliauskas P., Grybaitė B., Mickevičius V. (2021). Synthesis and Antibacterial Activity of New Azole, Diazole and Triazole Derivatives Based on p-Aminobenzoic Acid. Molecules.

[28] Strelčiūnaitė V., Jonuškienė I., Anusevičius K., Tumosienė I., Šiugždaitė J., Ramanauskaitė I., Mickevičius V. (2016). N-Synthesis of Novel Benzimidazoles 2-Functionalized with Pyrrolidinone and γ-Amino Acid with a High Antibacterial Activity. Heterocycles.

[29] Malūkaitė D., Grybaitė B., Vaickelionienė R., Vaickelionis G., Sapijanskaitė-Banevič B., Kavaliauskas P., Mickevičius V. (2022). Synthesis of Novel Thiazole Derivatives Bearing β-Amino Acid and Aromatic Moieties as Promising Scaffolds for the Development of New Antibacterial and Antifungal Candidates Targeting Multidrug-Resistant Pathogens. Molecules.

[30] Sapijanskaitė-Banevič B., Šovkovaja B., Vaickelionienė R., Šiugždaitė J., Mickevičiūtė E. (2020). Synthesis, Characterization and Bioassay of Novel Substituted 1-(3-(1,3-Thiazol-2-yl)phenyl)-5-oxopyrrolidines. Molecules.

[31] Voskienė A., Sapijanskaitė B., Mickevičius V., Jonuškienė I., Stasevych M., Komarovska-Porokhnyavets O., Musyanovych R., Novikov V. (2012). Synthesis and Microbiological Evaluation of New 2- and 2,3-Diphenoxysubstituted Naphthalene-1,4-diones with 5-Oxopyrrolidine Moieties. Molecules.

[32] Kairytė K., Grybaitė B., Vaickelionienė R., Sapijanskaitė-Banevič B., Kavaliauskas P., Mickevičius V. (2022). Synthesis and Biological Activity Characterization of Novel 5-Oxopyrrolidine Derivatives with Promising Anticancer and Antimicrobial Activity. Pharmaceuticals.

[33] Tumosienė I., Kantminienė K., Jonuškienė I., Peleckis A., Belyakov S., Mickevičius V. (2019). Synthesis of 1-(5-Chloro-2-hydroxyphenyl)-5-oxopyrrolidine-3-carboxylic Acid Derivatives and Their Antioxidant Activity. Molecules.

[34] Brokaitė K., Mickevičius V., Mikulskienė G. (2006). Synthesis and structural investigation of some 1,4-disubstituted-2-pyrrolidinones. Arkivoc.

[35] Nath R., Pathania S., Grover G., Akhtar M.J. (2020). Isatin containing heterocycles for different biological activities: Analysis of structure activity relationship. J. Mol. Struct..

[36] Karthikeyan C., Amawi H., Ashby C.R., Khare V.M., Jones V., Moorthy N.S.H.N., Trivedi P., Tiwari A.K. (2019). Novel 3-((2-chloroquinolin-3-yl)methylene)indolin-2-one derivatives produce anticancer efficacy in ovarian cancer *in vitro*. Heliyon.

[37] Mickevicius M., Beresnevicius Z.J., Mickevicius V., Mikulskiene G. (2006). Condensation products of 1-aryl-4-carboxy-2-pyrrolidinones with *o*-diaminoarenes, *o*-aminophenol, and their structural studies. Heteroat. Chem..

[38] Marturano J.E., Lowery T.J. (2019). ESKAPE Pathogens in Bloodstream Infections Are Associated with Higher Cost and Mortality but Can Be Predicted Using Diagnoses Upon Admission. Open Forum Infect. Dis..

[39] Petraitis V., Petraitiene R., Kavaliauskas P., Naing E., Garcia A., Sutherland C., Kau A.Y., Goldner N., Bulow C., Nicolau D.P. (2021). Pharmacokinetics, Tissue Distribution, and Efficacy of VIO-001 (Meropenem/Piperacillin/Tazobactam) for Treatment of Methicillin-Resistant Staphylococcus aureus Bacteremia in Immunocompetent Rabbits with Chronic Indwelling Vascular Catheters. Antimicrob. Agents Chemother..

[40] Kavaliauskas P., Grybaite B., Mickevicius V., Petraitiene R., Grigaleviciute R., Planciuniene R., Gialanella P., Pockevicius A., Petraitis V. (2020). Synthesis, ADMET Properties, and In Vitro Antimicrobial and Antibiofilm Activity of 5-Nitro-2-thiophenecarbaldehyde N-((E)-(5-Nitrothienyl)methylidene)hydrazone (KTU-286) against *Staphylococcus aureus* with Defined Resistance Mechanisms. Antibiotics.

[41] Clinical and Laboratory Standards Institute (2009). Methods for Dilution Antimicrobial Susceptibility Tests for Bacteria That Grow Aerobically.

[42] Hastey C.J., Dale S.E., Nary J., Citron D., Law J.H., Roe-Carpenter D.E., Chesnel L. (2017). Comparison of Clostridium difficile minimum inhibitory concentrations obtained using agar dilution vs broth microdilution methods. Anaerobe.

