# Supplementary material for: Synthesis of 1-(2-Hydroxyphenyl)- and (3,5-Dichloro-2-hydroxyphenyl)-5-oxopyrrolidine-3-carboxylic Acid Derivatives as Promising Scaffolds for the Development of Novel Antimicrobial and Anticancer Agents
Source: Int J Mol Sci. 2023 Apr 27;24(9):7966. doi: 10.3390/ijms24097966 (PMC10178429; doi:10.3390/ijms24097966)
Supplement: Supplementary file 1 [file ijms-24-07966-s001.zip › ijms-2342823-Supplementary Materials.pdf]

Synthesis of 1-(2-hydroxyphenyl)- and (3,5-dichloro-2-hydroxyphenyl)-5-oxopyrrolidine-3-carboxylic acids derivatives as promising scaffolds for the development of novel antimicrobial and anticancer agents

Monika Bertašiūtė, Povilas Kavaliauskas, Rita Vaickelionienė, Birutė Grybaitė, Vidmantas Petraitis, Rūta Petraitienė, Ethan Naing, Andrew Garcia, Jūratė Šiugždaitė, Raimundas Lelešius and Vytautas Mickevičius

Supplementary Materials

^{1}H and ^{13}C NMR spectra of compounds 1a, b, 2a and 3–28b.

HRMS spectra of compounds 14, 15, 24b and 28b.

Figure S67

1-(2-Hydroxyphenyl)-5-oxopyrrolidine-3-carboxylic acid (1a).

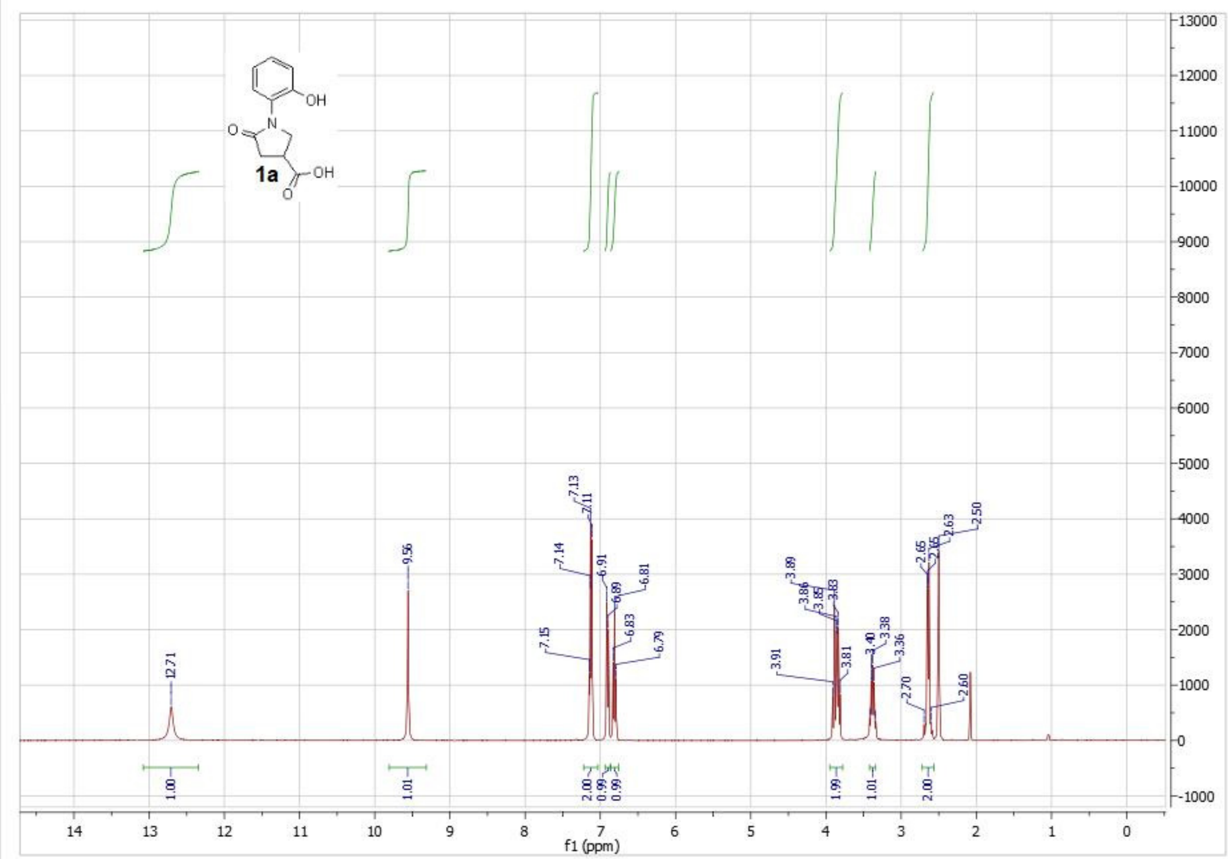

Figure S1. ^{1}H NMR spectrum of compound **1a.**

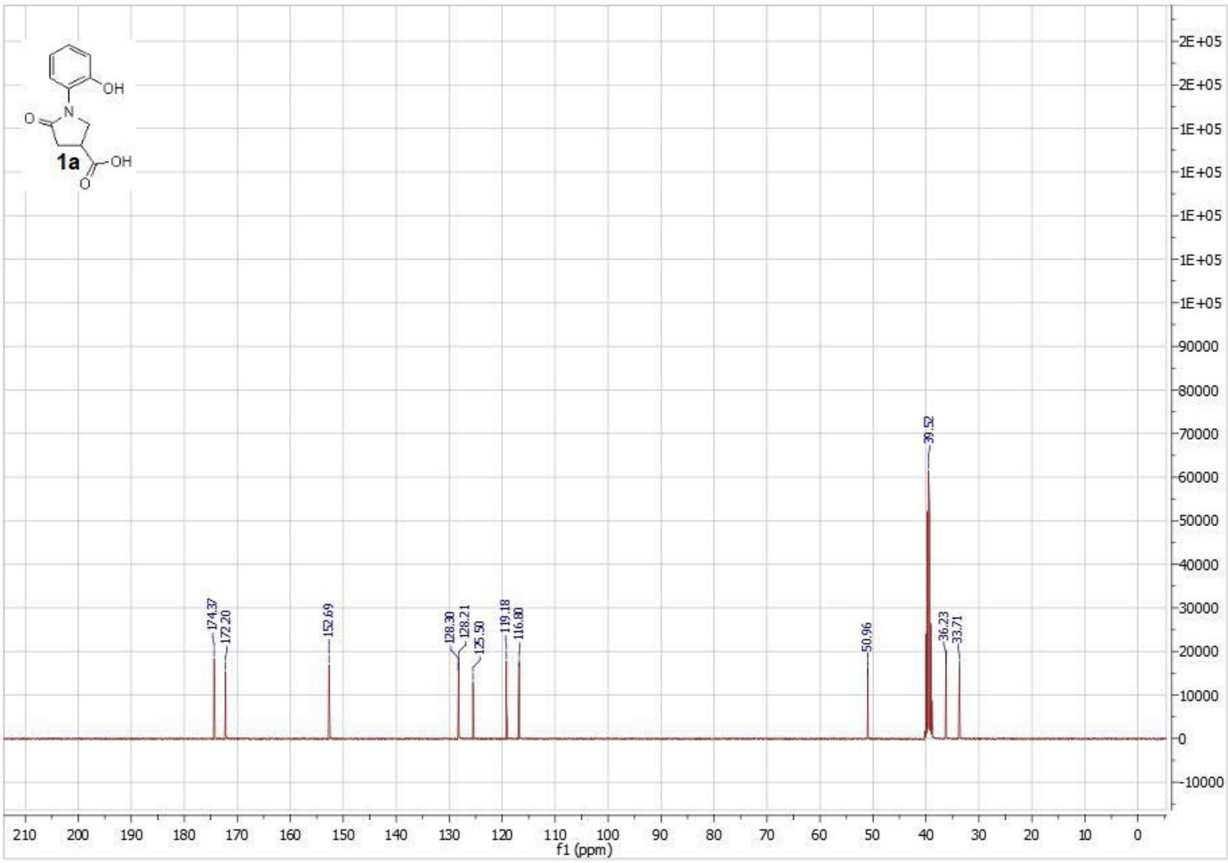

Figure S2. ^{13}C NMR spectrum of compound **1a.**

Methyl 1-(2-hydroxyphenyl)-5-oxopyrrolidine-3-carboxylate (2a)

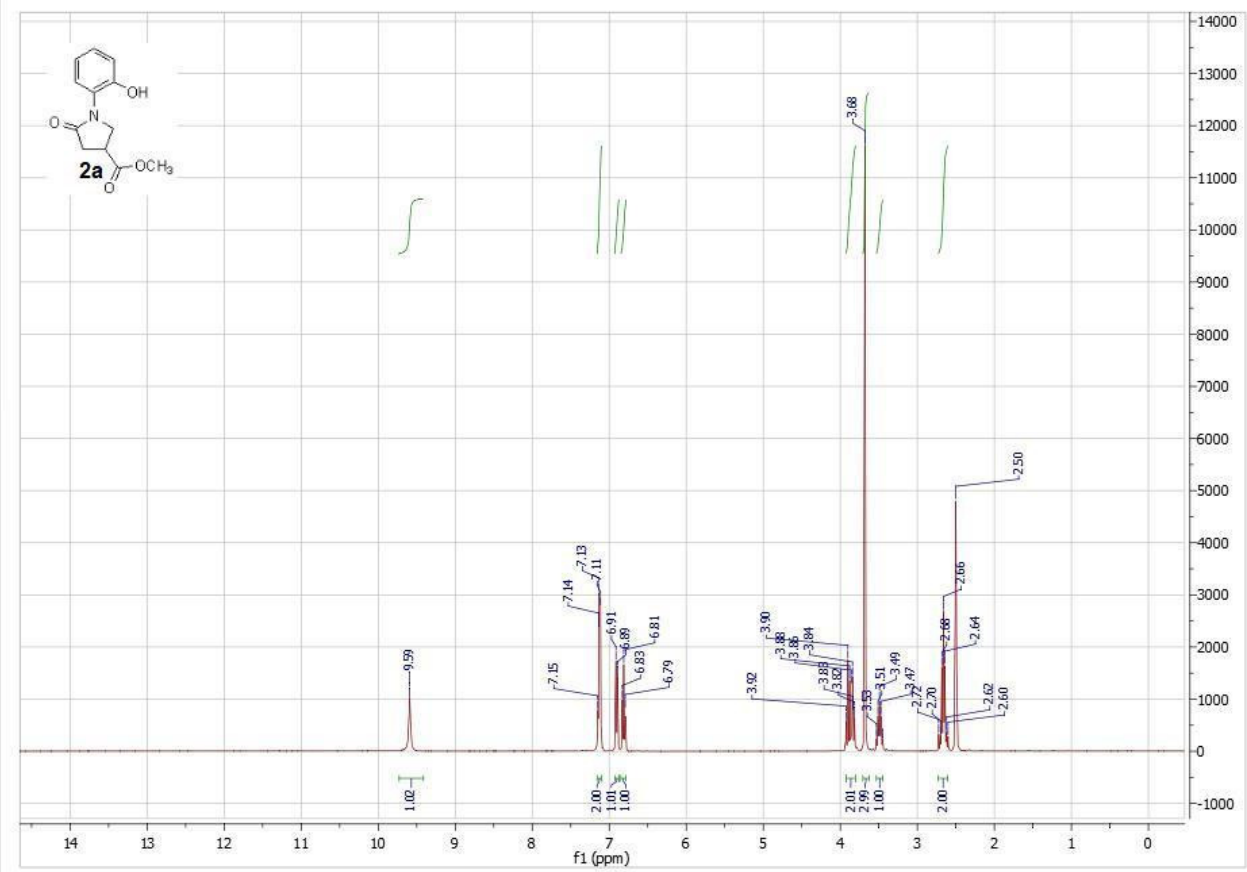

Figure S3. ^{1}H NMR spectrum of compound **2a.**

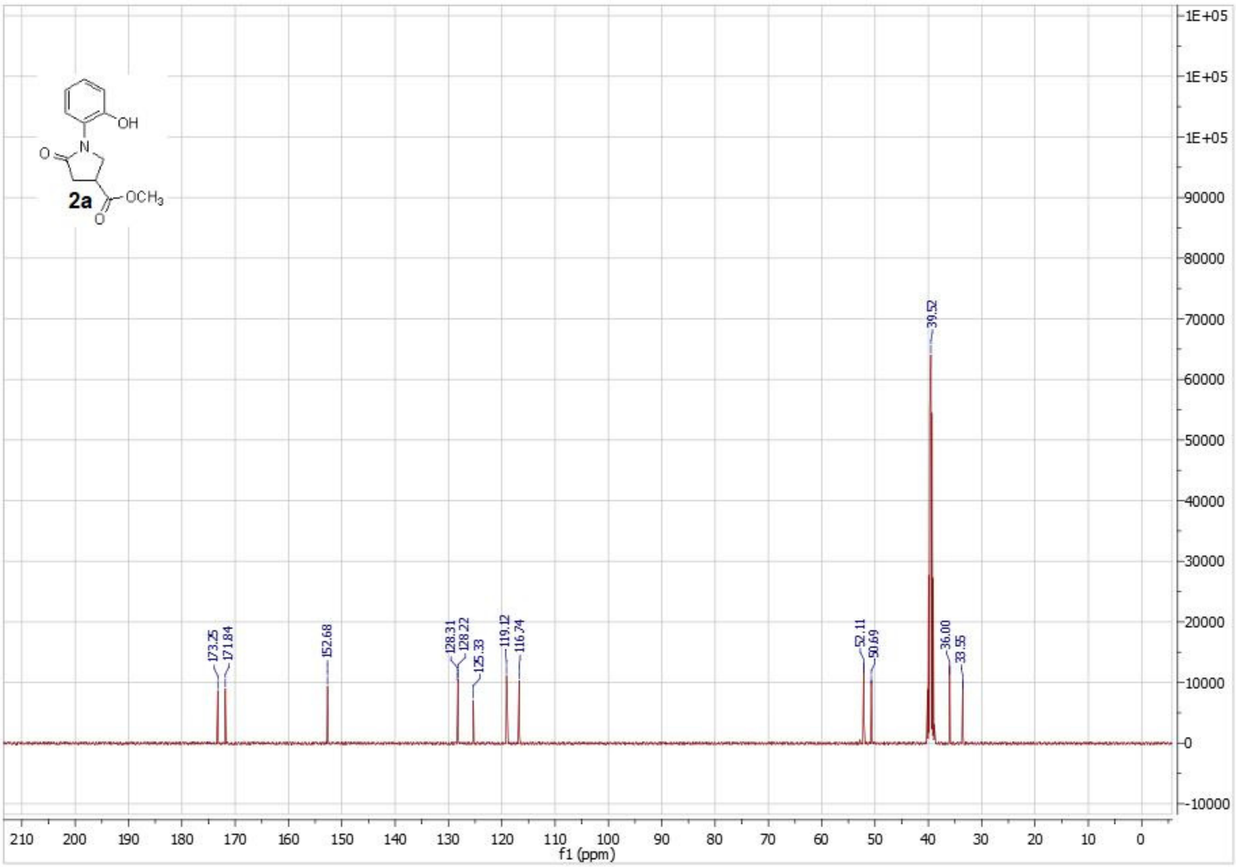

Figure S4. ^{13}C NMR spectrum of compound **2a.**

1-(2-Hydroxyphenyl)-5-oxopyrrolidine-3-carbohydrazide (3).

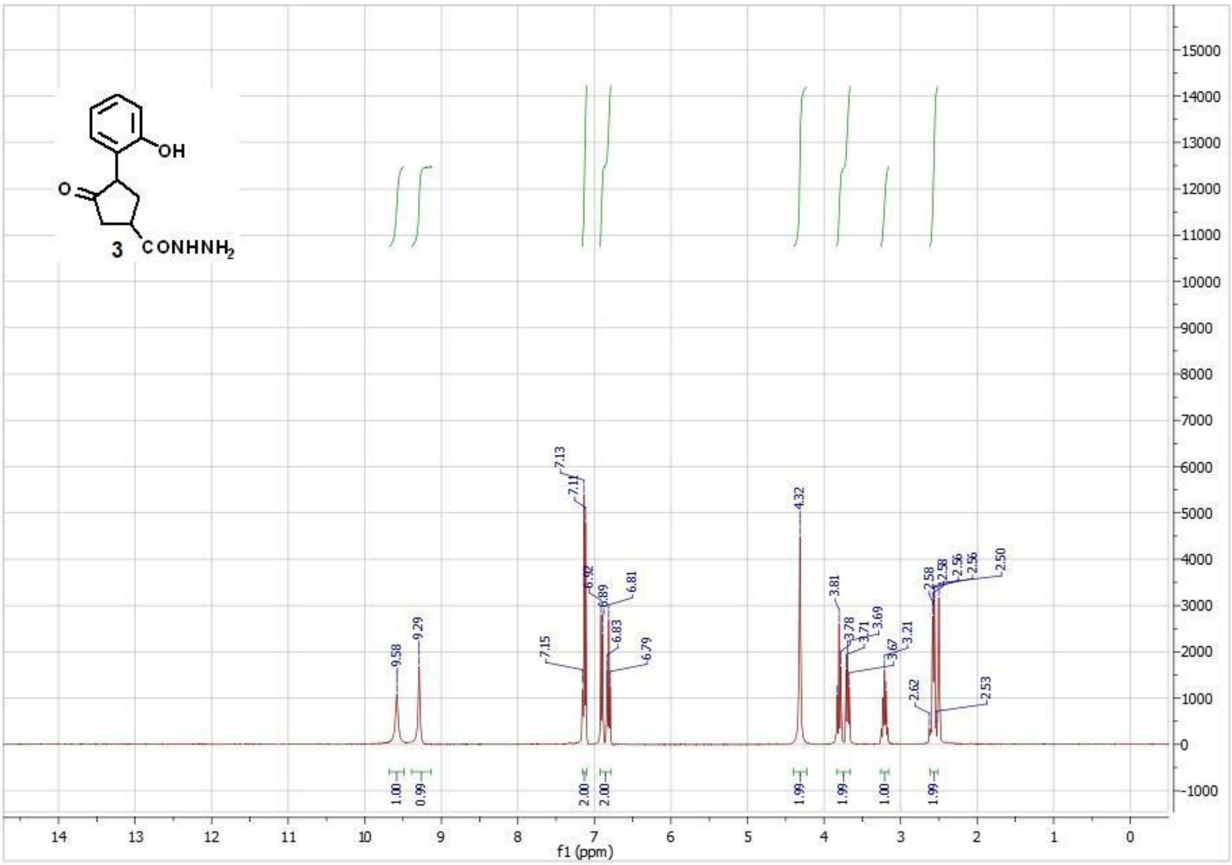

Figure S5. ^{1}H NMR spectrum of compound **3.**

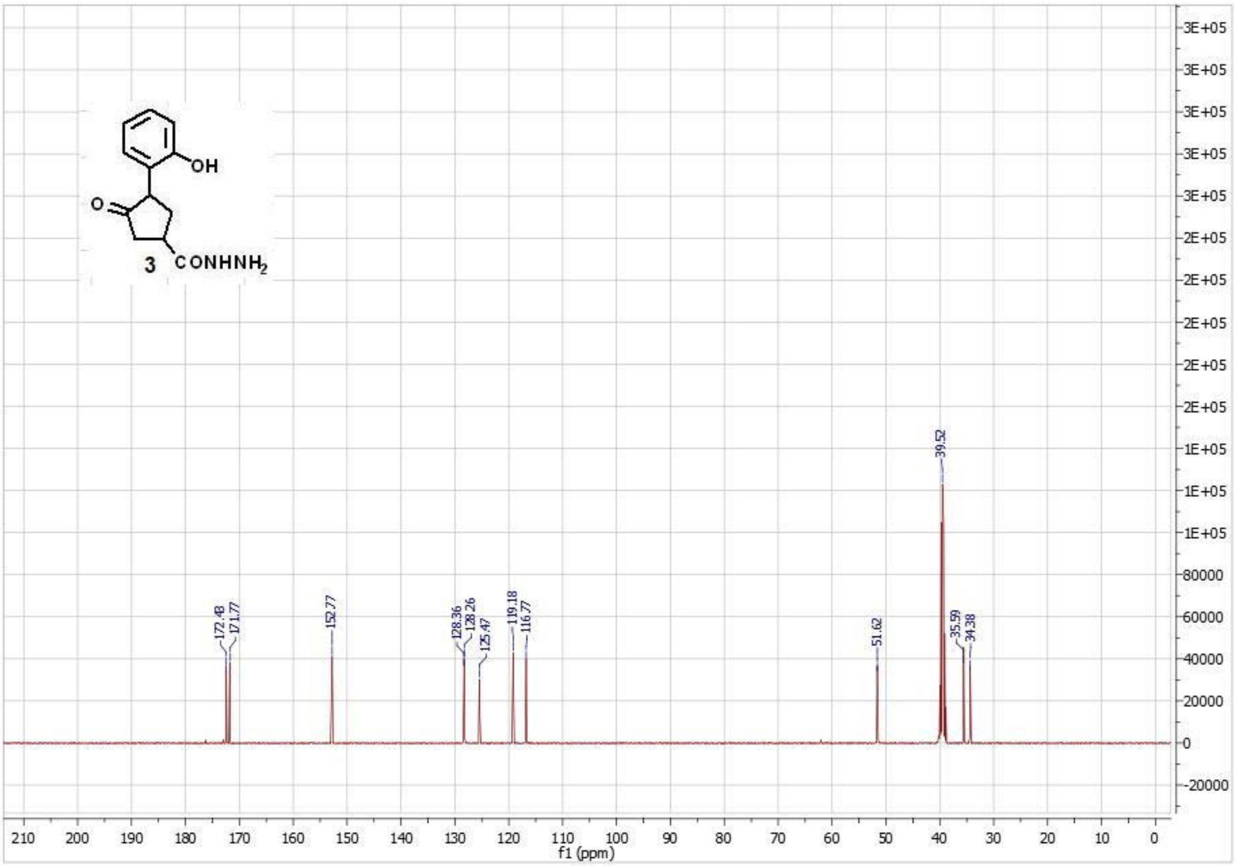

Figure S6. ^{13}C NMR spectrum of compound **3.**

***N′*-benzylidene-1-(2-hydroxyphenyl)-5-oxopyrrolidine-3-carbohydrazide (4).**

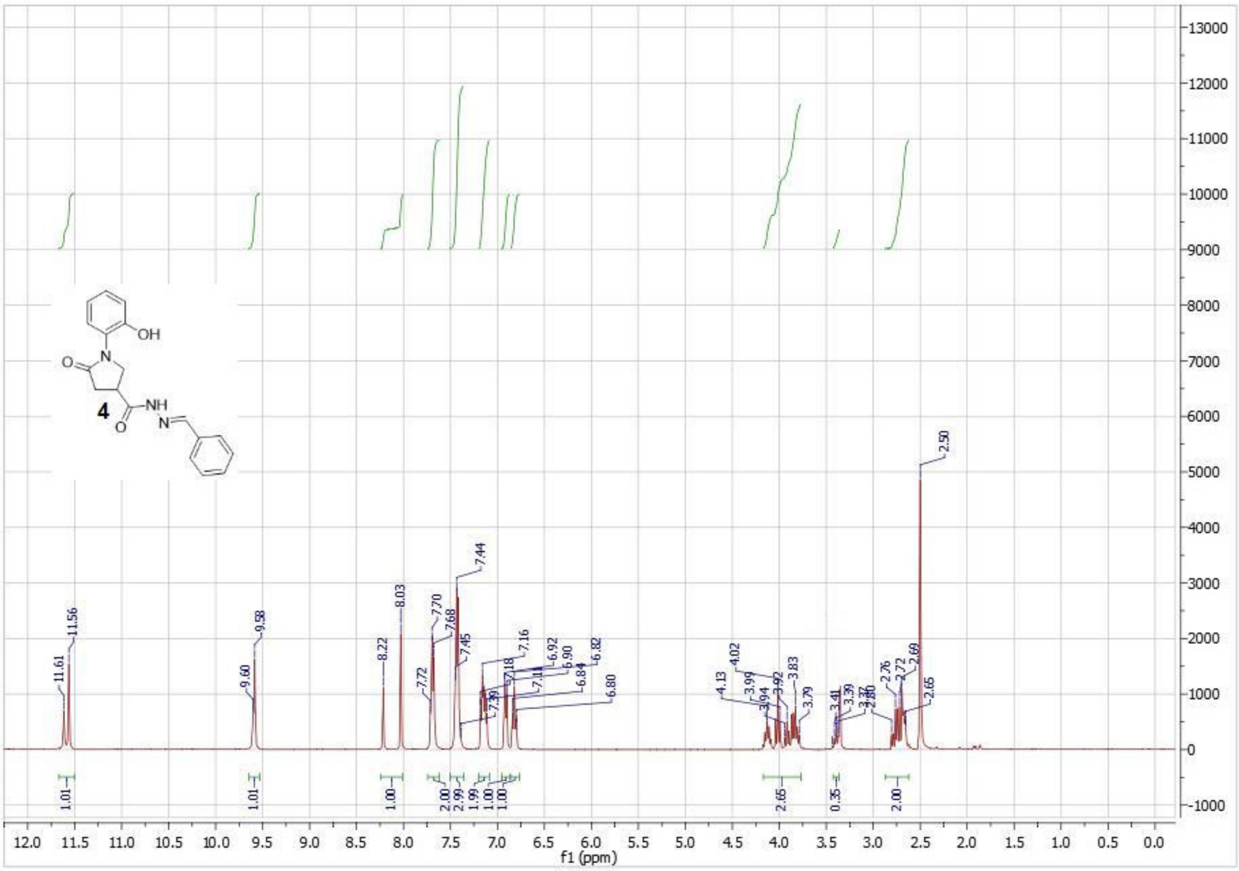

Figure S7. ^{1}H NMR spectrum of compound **4.**

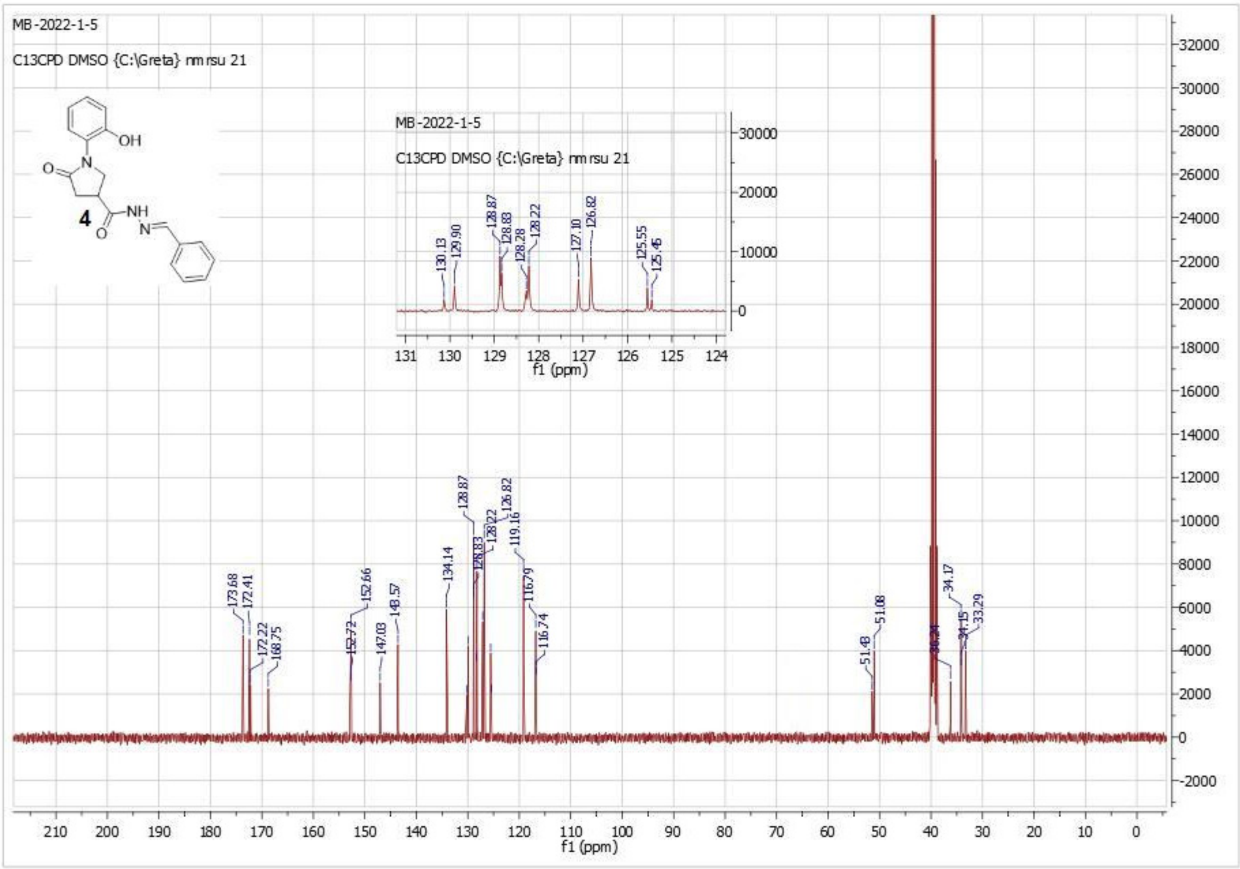

Figure S8. ^{13}C NMR spectrum of compound **4.**

***N′*-(4-chlorobenzylidene)-1-(2-hydroxyphenyl)-5-oxopyrrolidine-3-carbohydrazide (5).**

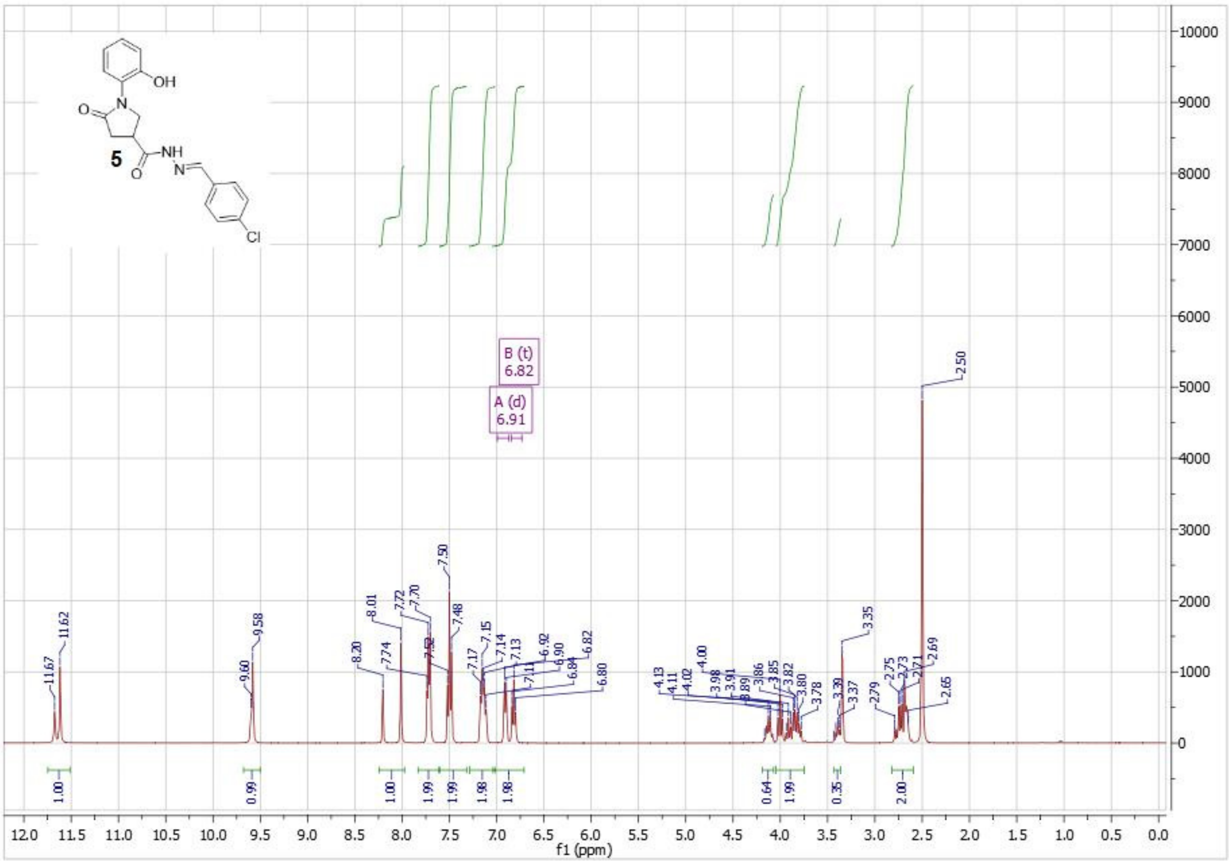

Figure S9. ^{1}H NMR spectrum of compound **5.**

***N′*-(4-bromobenzylidene)-1-(2-hydroxyphenyl)-5-oxopyrrolidine-3-carbohydrazide (6).**

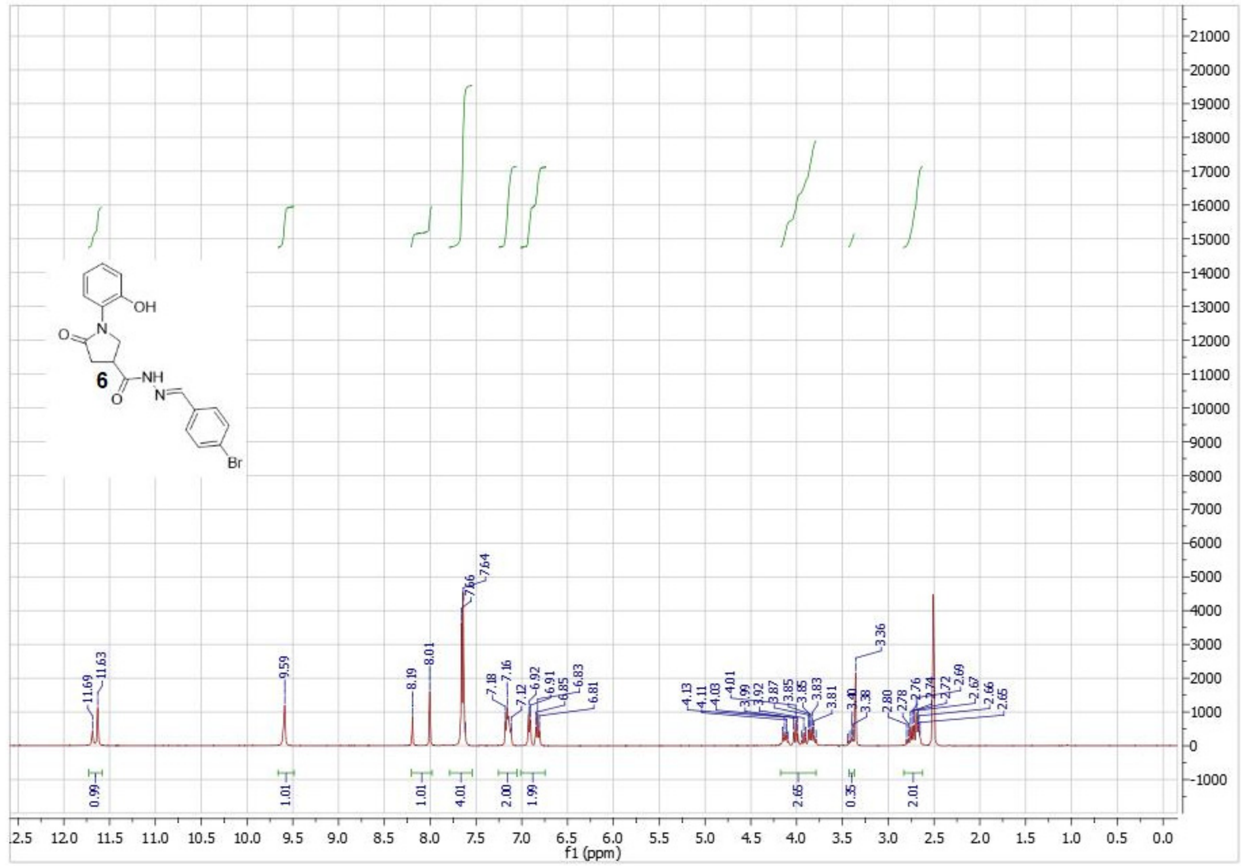

Figure S10. ^{1}H NMR spectrum of compound **6.**

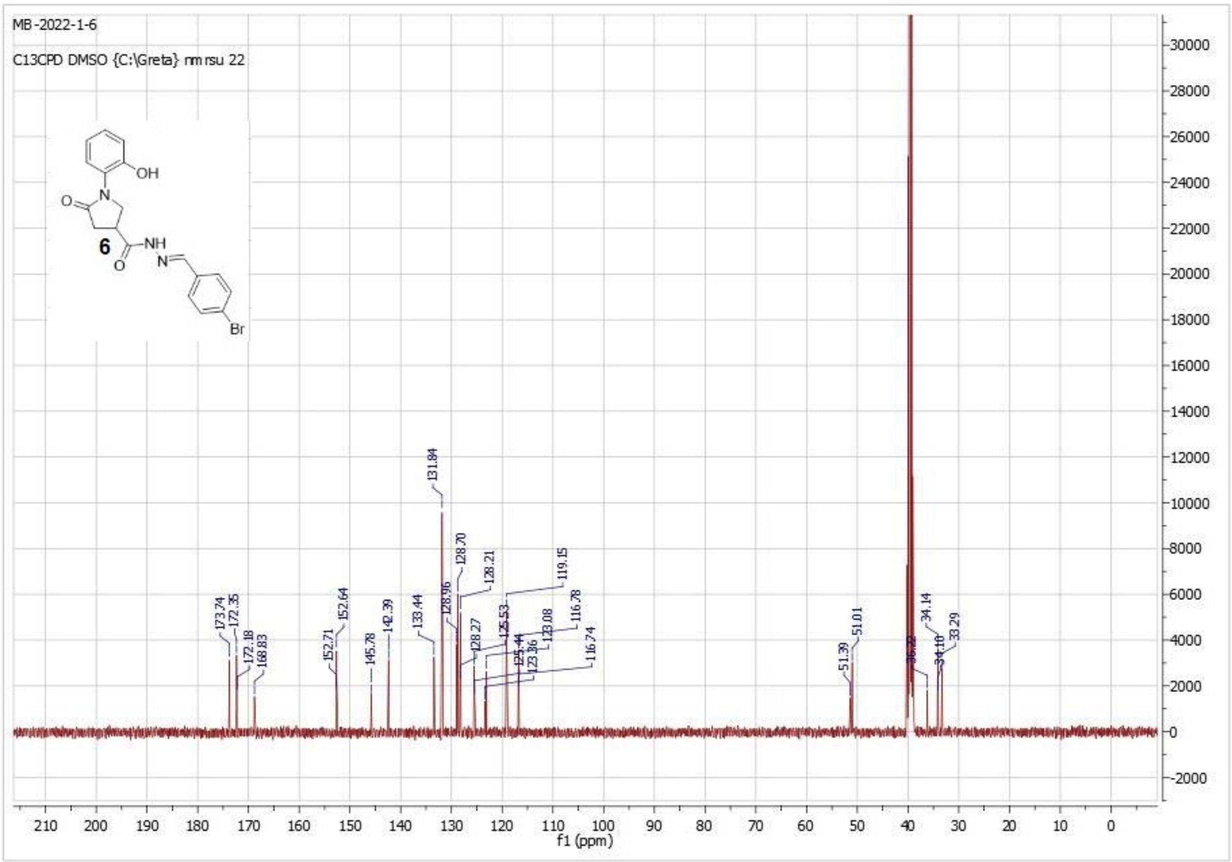

Figure S11. ^{13}C NMR spectrum of compound **6**.

1-(2-Hydroxyphenyl)-*N′*-(4-nitrobenzylidene)-5-oxopyrrolidine-3-carbohydrazide (7).

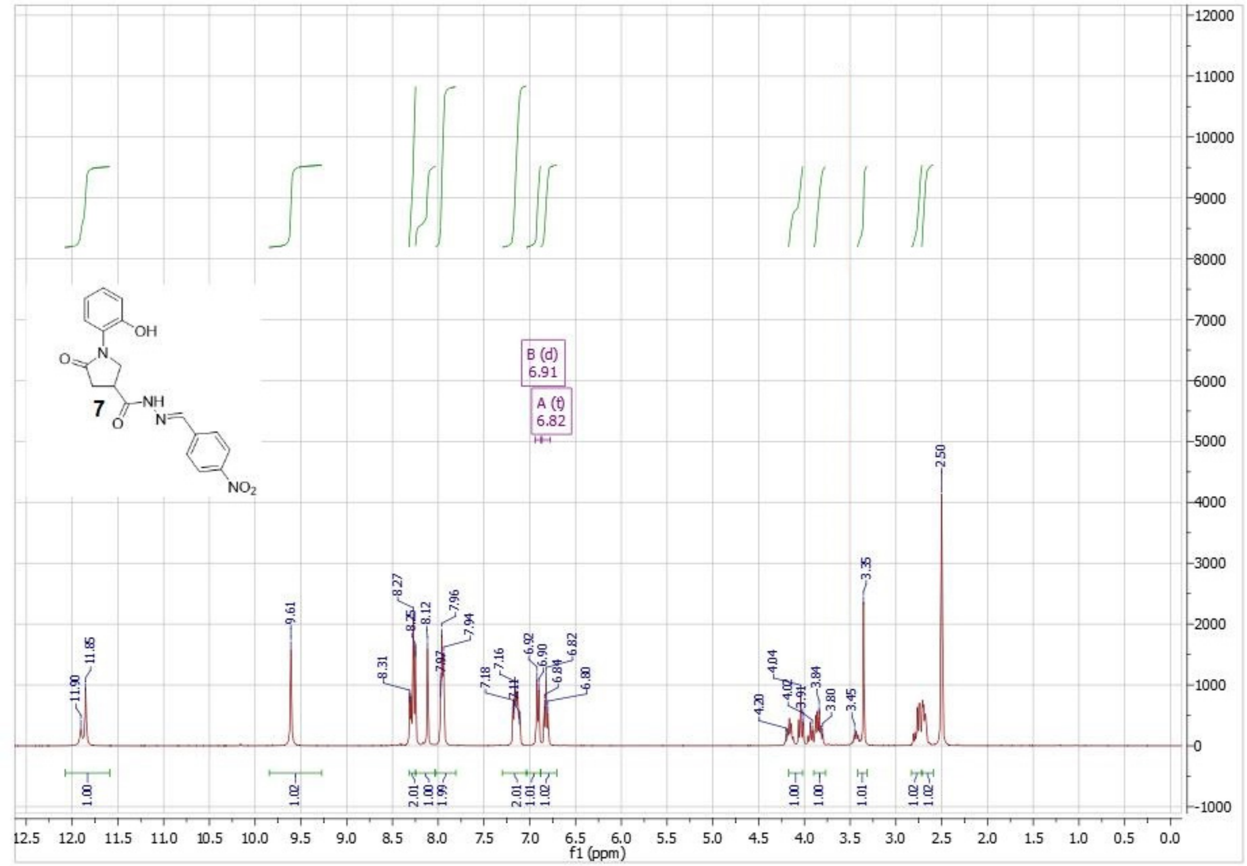

Figure S12. ^{1}H NMR spectrum of compound **7.**

***N′*-(4-(dimethylamino)benzylidene)-1-(2-hydroxyphenyl)-5-oxopyrrolidine-3-carbohydrazide (8).**

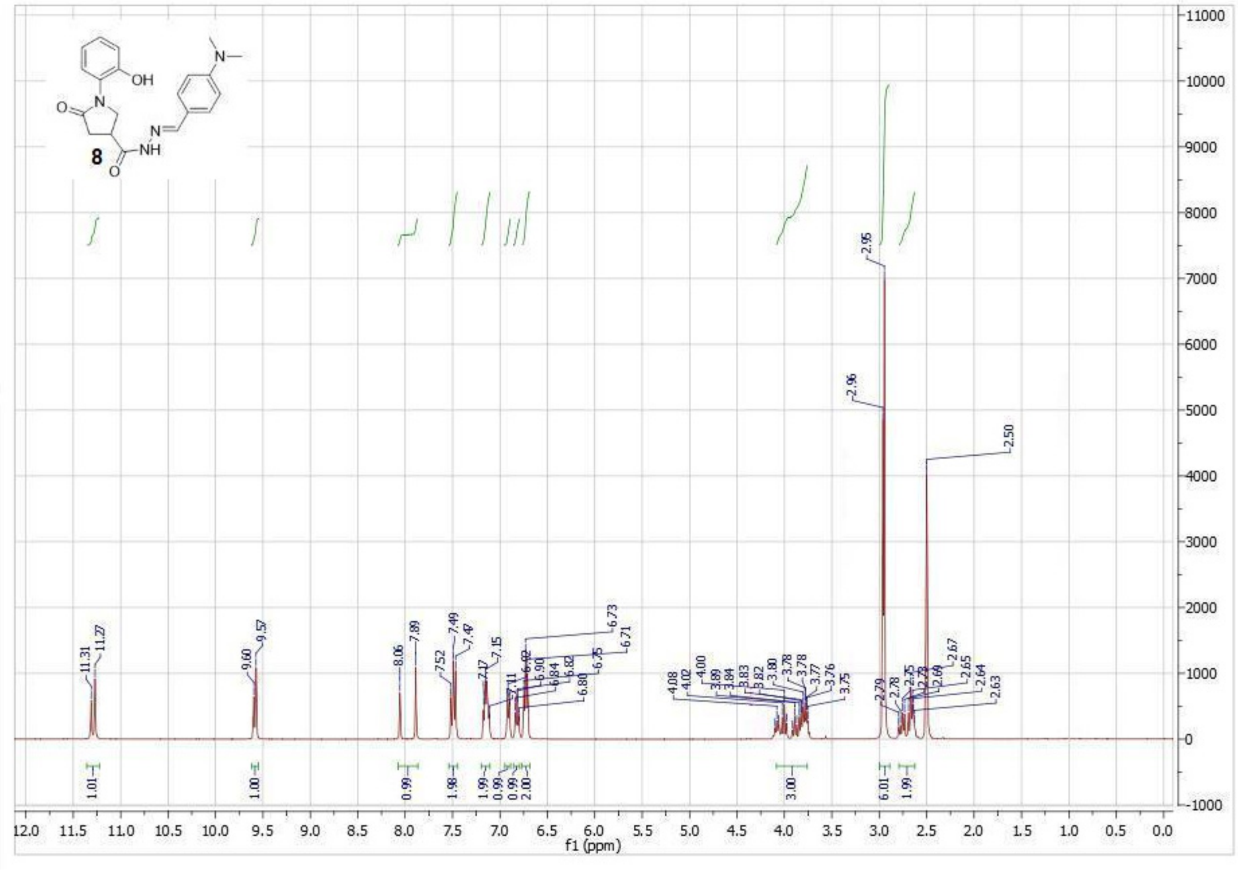

Figure S13. ¹H NMR spectrum of compound **8.**

1-(2-Hydroxyphenyl)-*N′*-(4-methoxybenzylidene)-5-oxopyrrolidine-3-carbohydrazide (9).

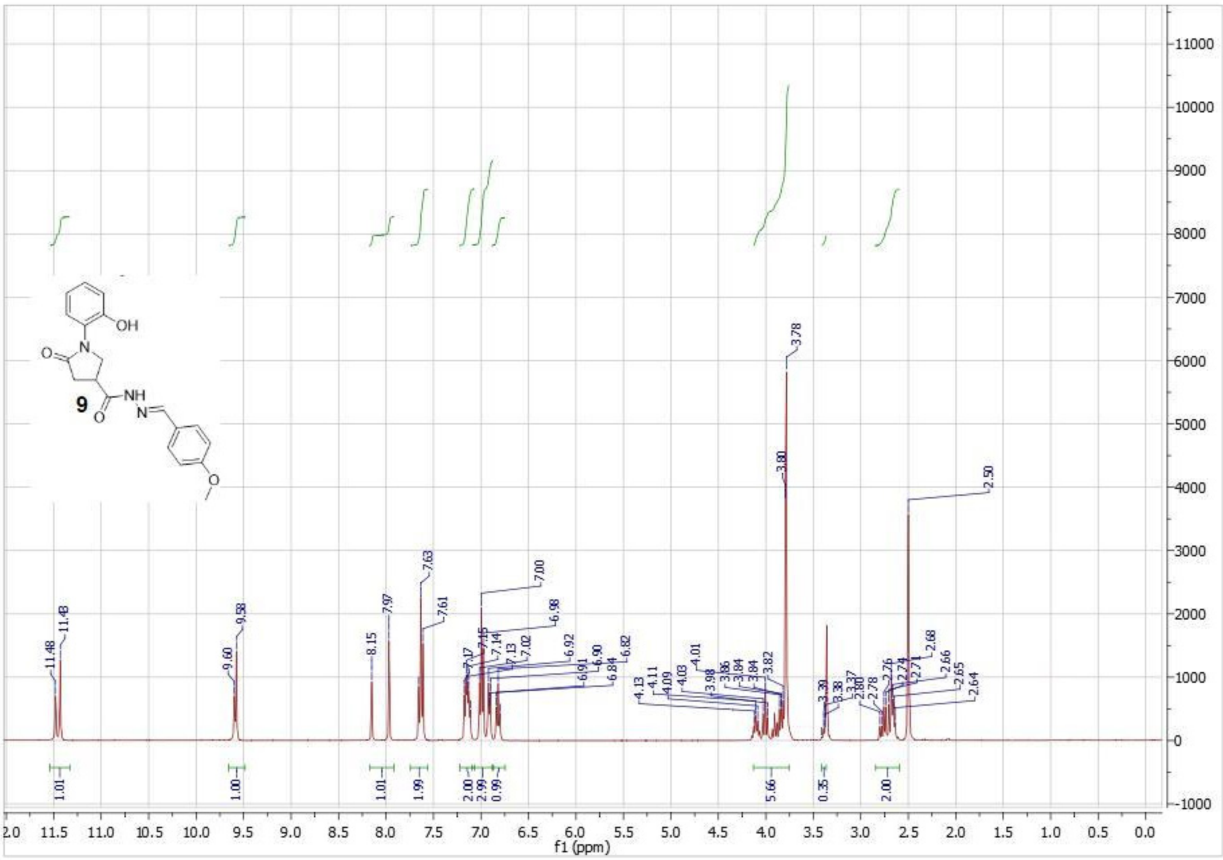

Figure S14. ¹H NMR spectrum of compound **9.**

1-(2-Hydroxyphenyl)-*N′*-(2,4-dimethoxybenzylidene)-5-oxopyrrolidine-3-carbohydrazide (10).

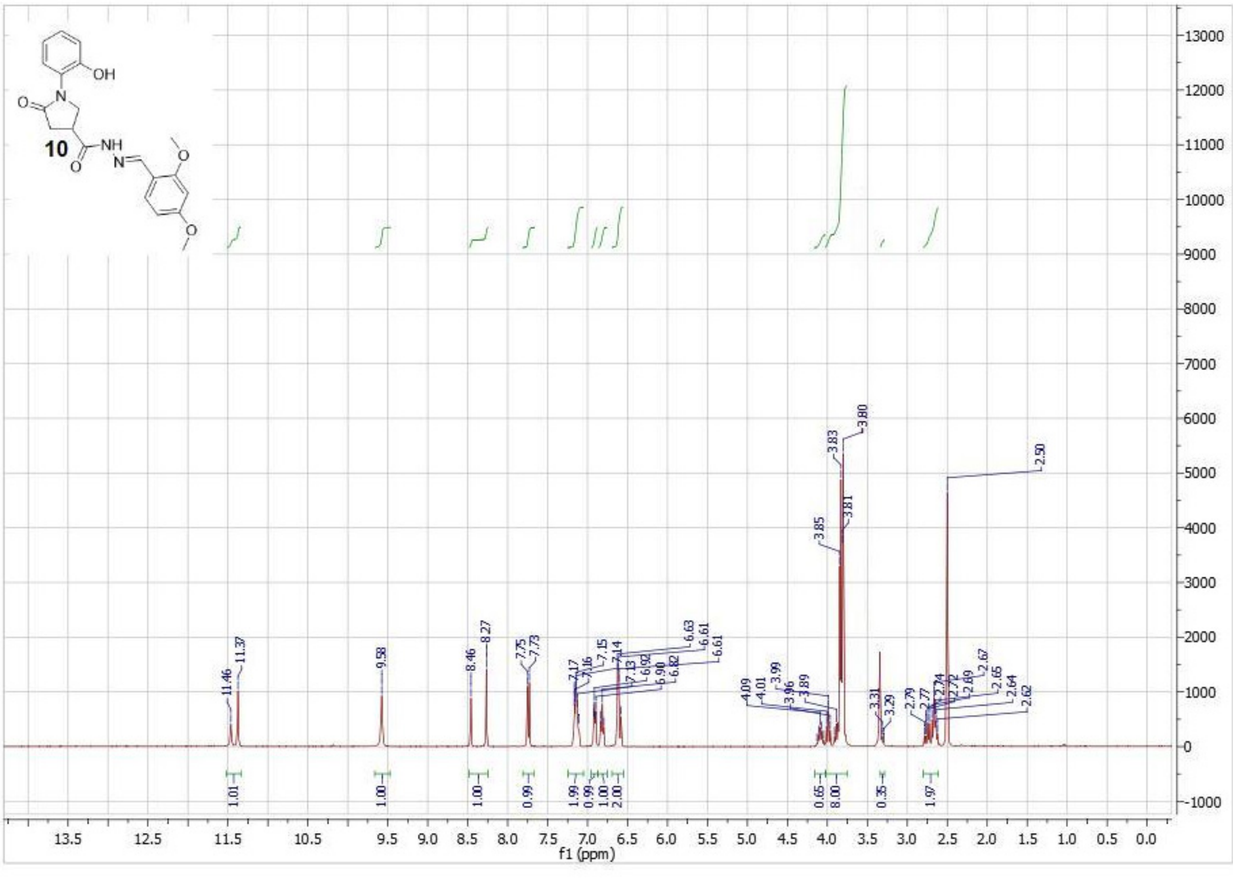

Figure S15. ^{13}C NMR spectrum of compound **10.**

1-(2-Hydroxyphenyl)-*N′*-(2,3,4-trimethoxybenzylidene)-5-oxopyrrolidine-3-carbohydrazide (11).

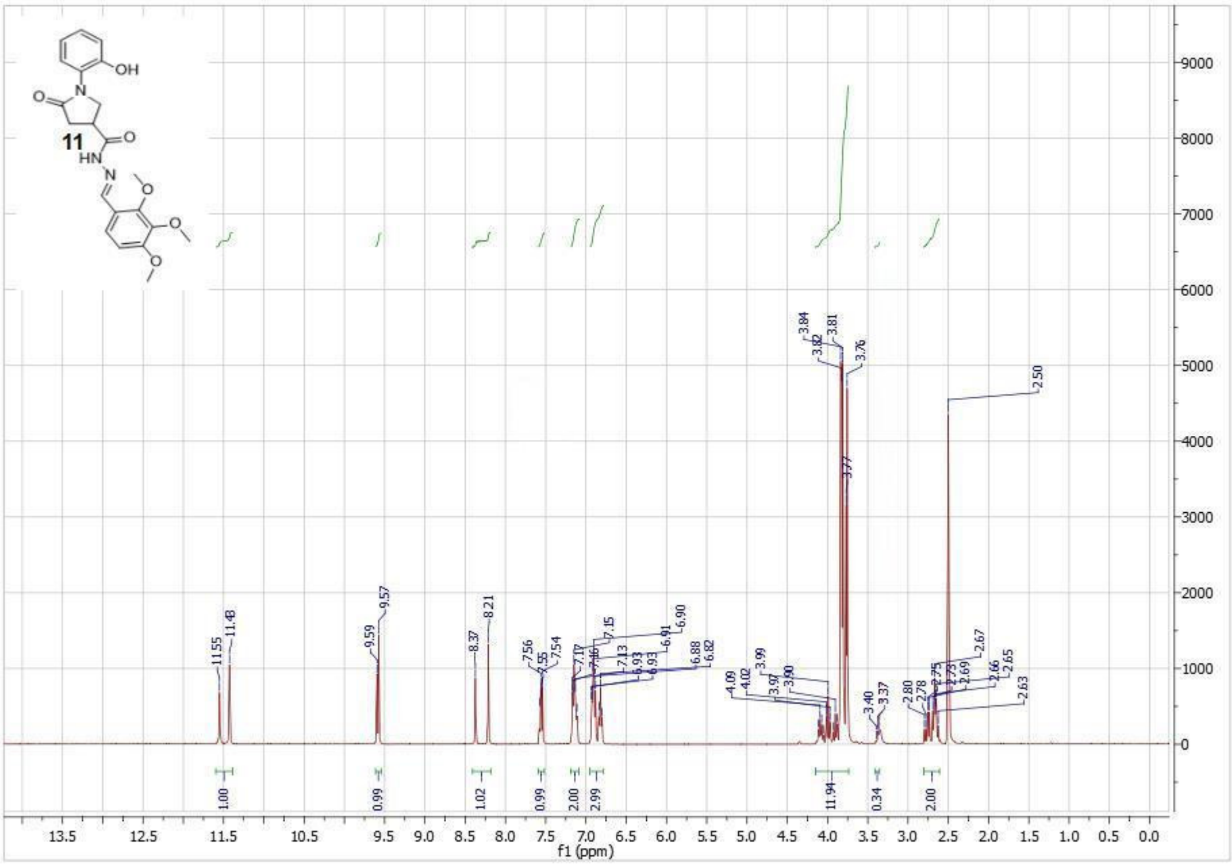

Figure S16. ^{1}H NMR spectrum of compound **11.**

1-(2-Hydroxyphenyl)-*N′*-(3,4,5-trimethoxybenzylidene)-5-oxopyrrolidine-3-carbohydrazide (12).

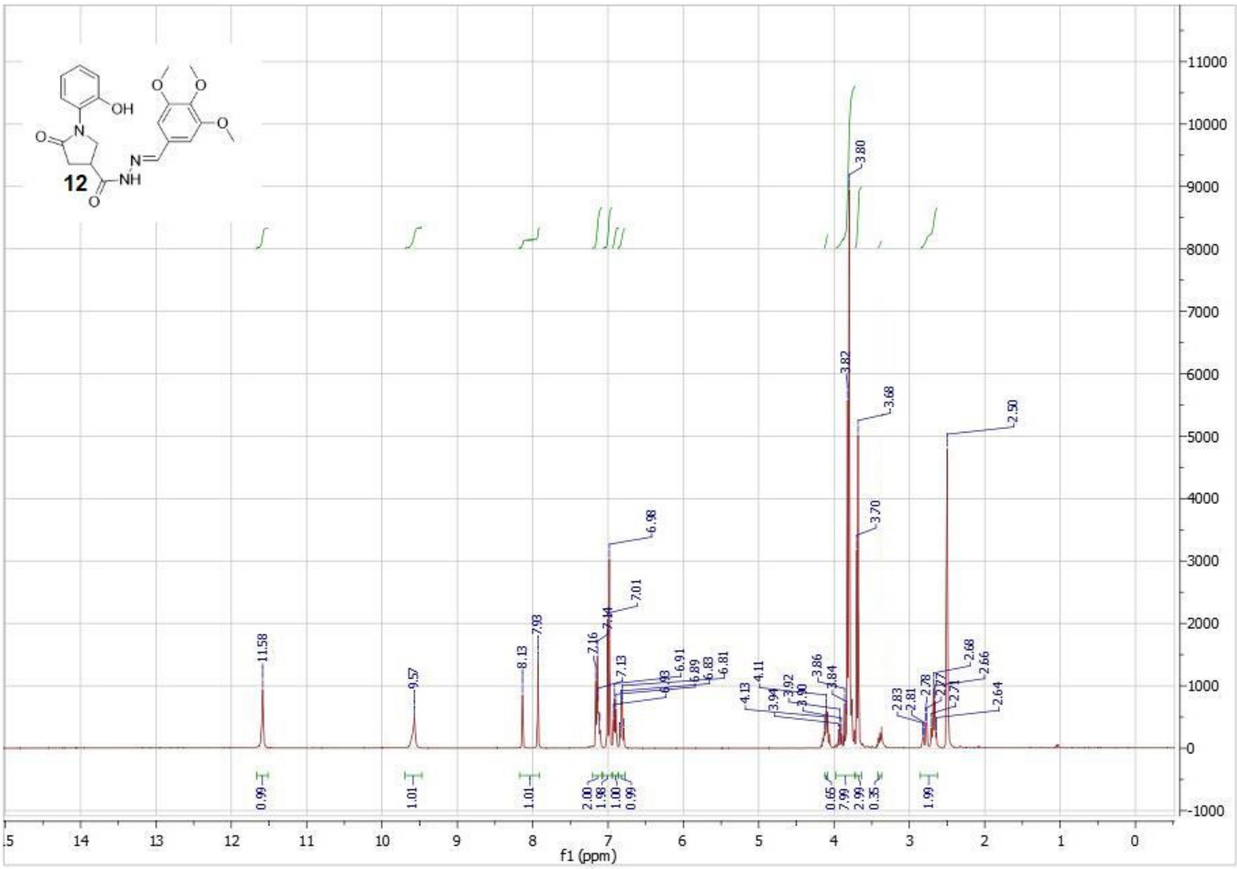

Figure S17. ^{1}H NMR spectrum of compound **12.**

1-(2-Hydroxyphenyl)-*N*'-(naphth-1-ylmethylene)-5-oxopyrrolidine-3-carbohydrazide (13)

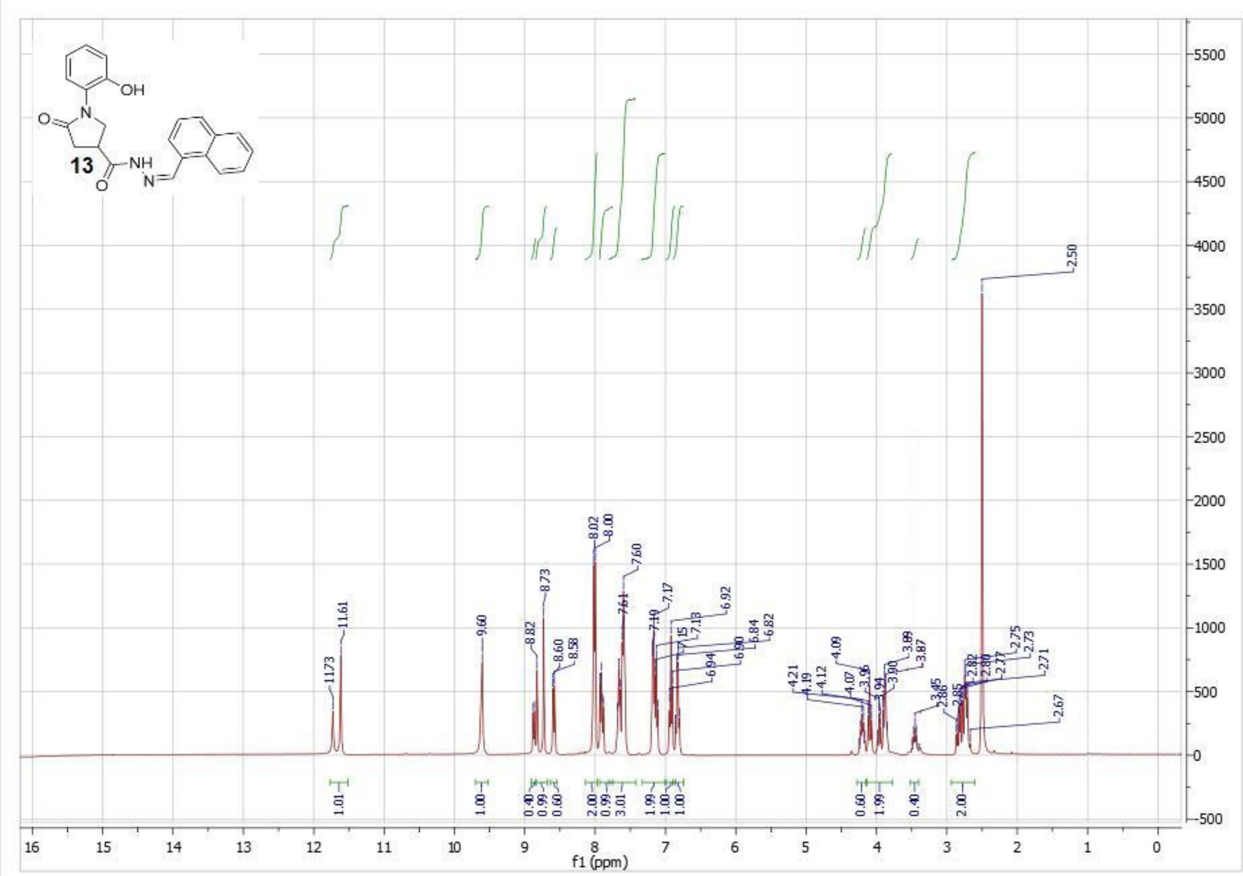

Figure S18. ^{1}H NMR spectrum of compound **13.**

1-(2-Hydroxyphenyl)-5-oxo-*N'*-(thien-2-ylmethylene)pyrrolidine-3-carbohydrazide (14)

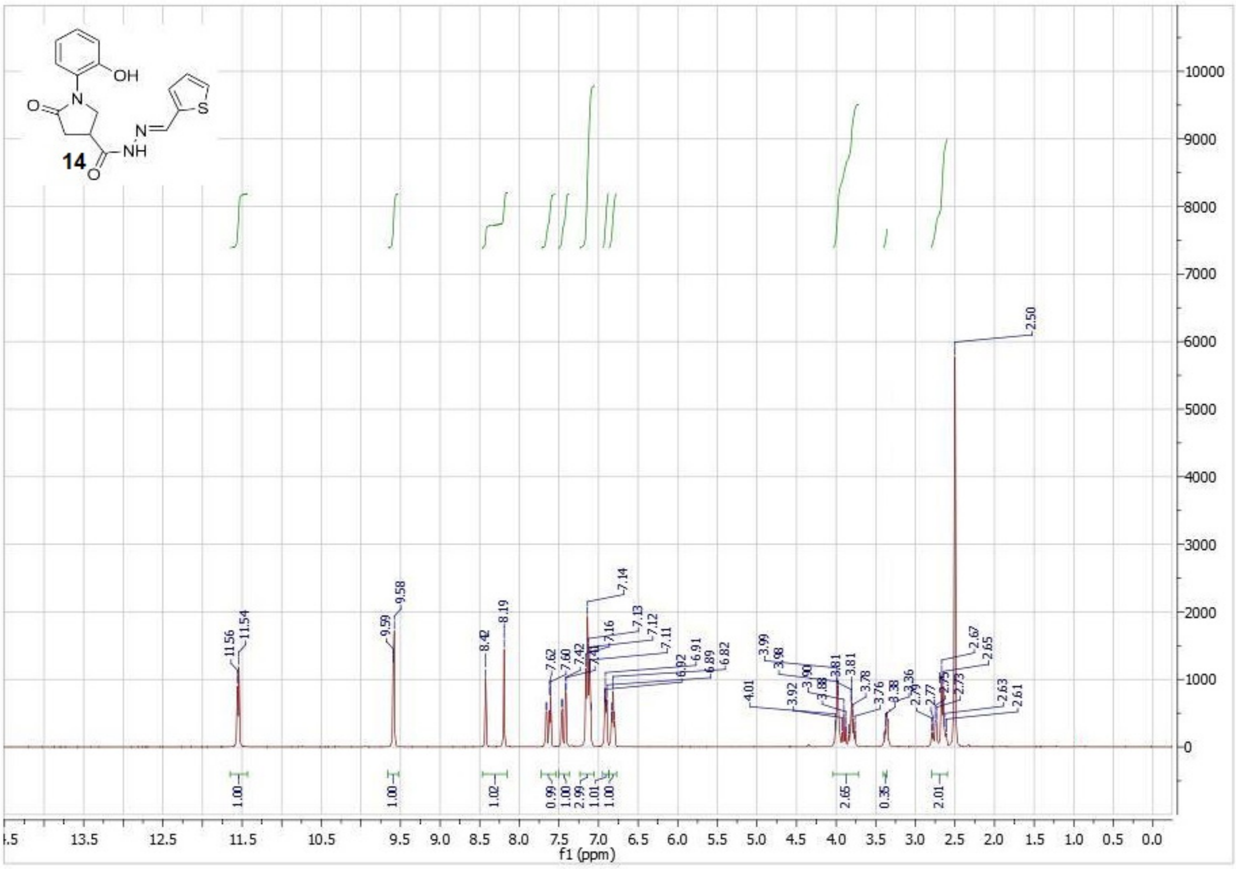

Figure S19. ^{1}H NMR spectrum of compound **14.**

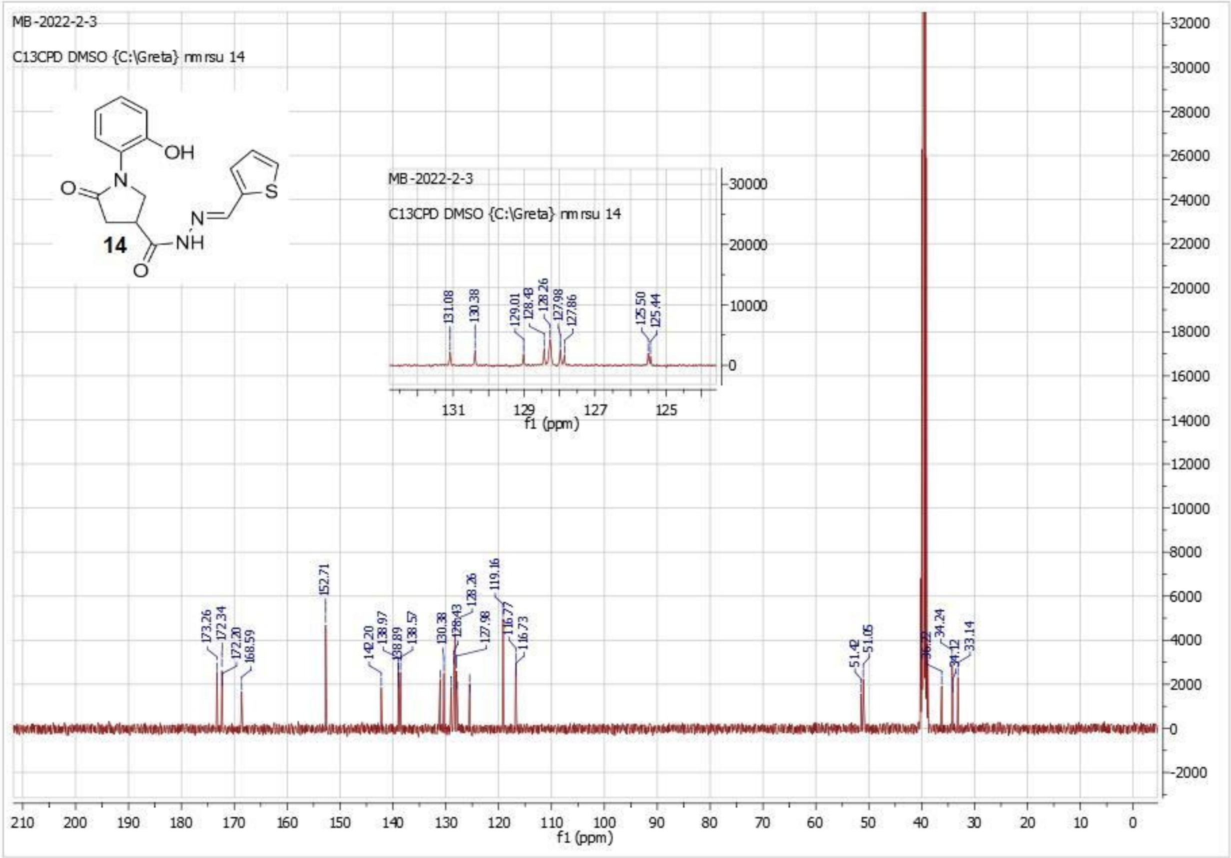

Figure S20. ^{13}C NMR spectrum of compound **14.**

1-(2-Hydroxyphenyl)-5-oxo-*N'*-(5-nitrothien-2-ylmethylene)pyrrolidine-3-carbohydrazide (15)

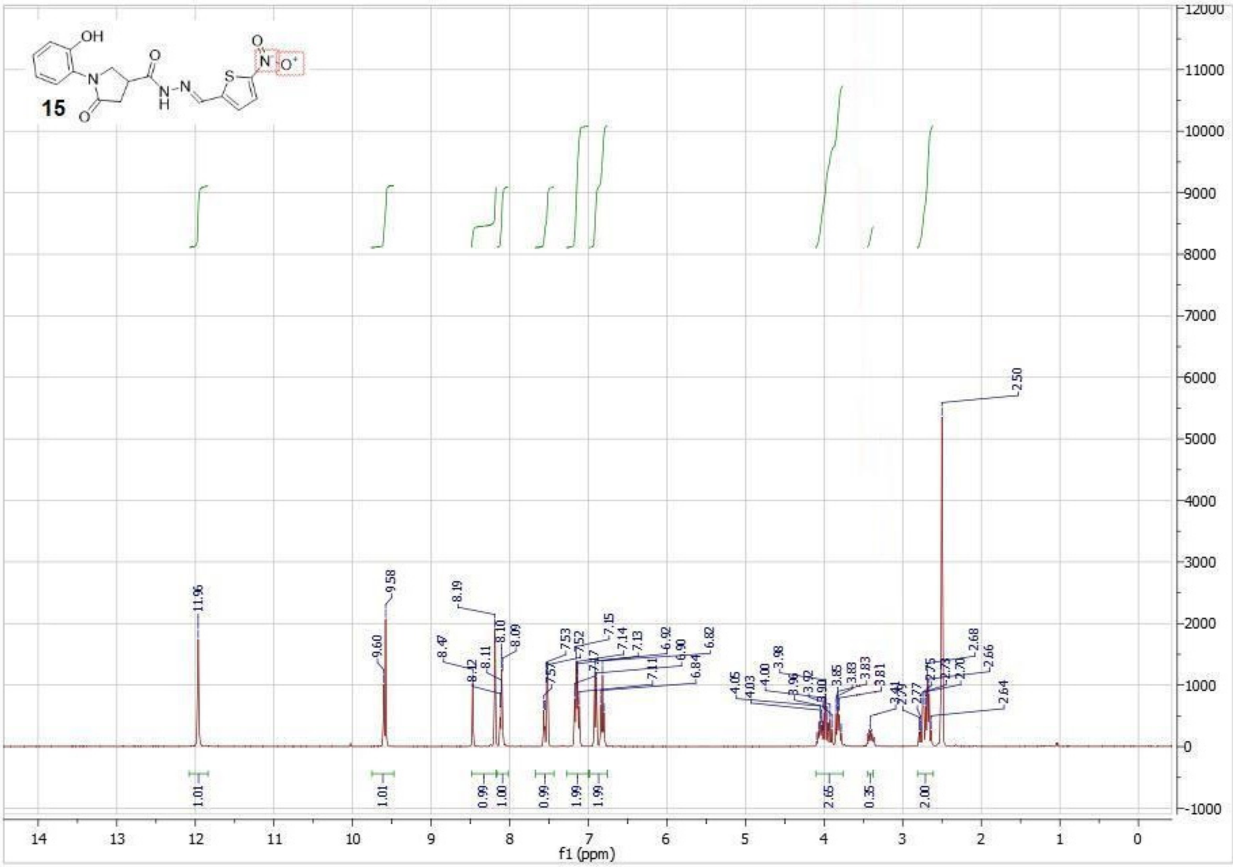

Figure S21 ^{1}H NMR spectrum of compound **15.**

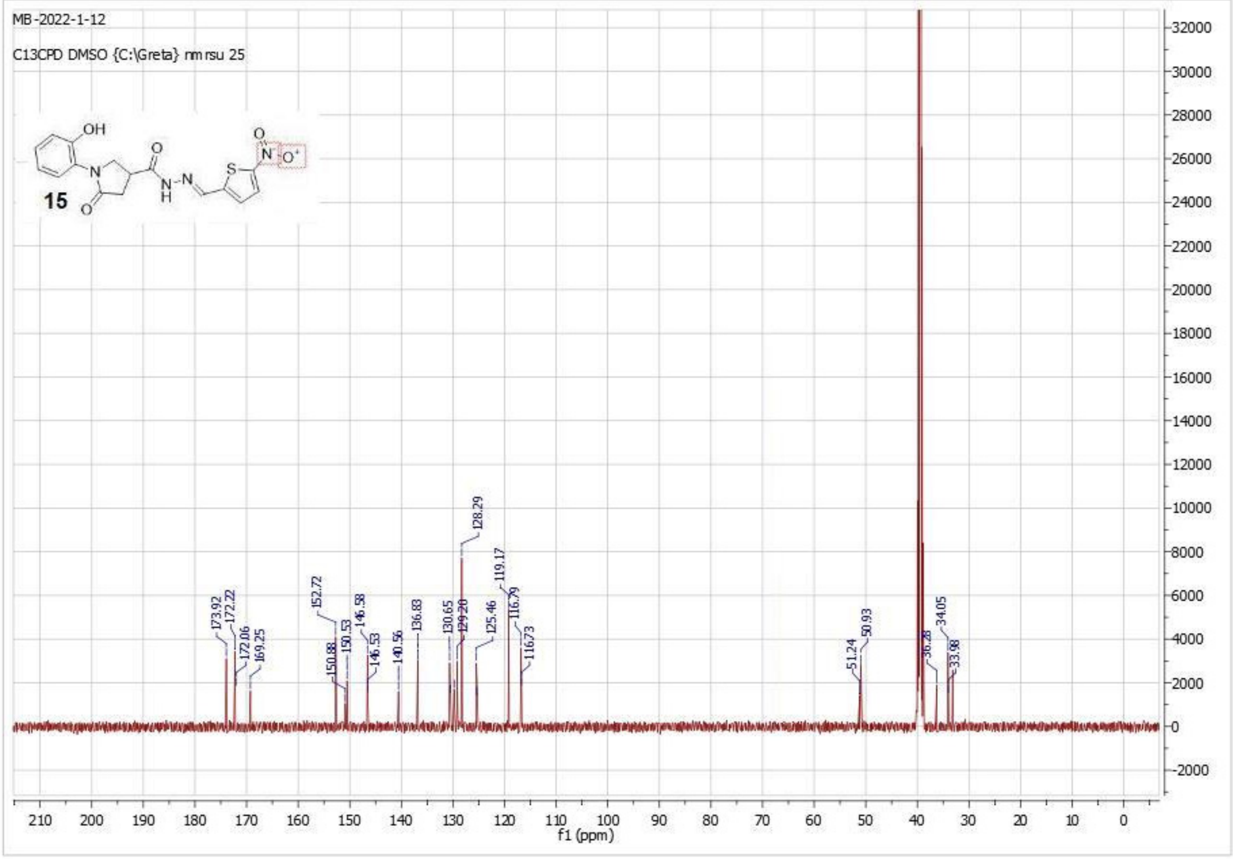

Figure S22. ^{13}C NMR spectrum of compound **15.**

***N*'-(1-(4-aminophenyl)ethylidene)-1-(2-hydroxyphenyl)-5-oxopyrrolidine-3-carbohydrazide (16).**

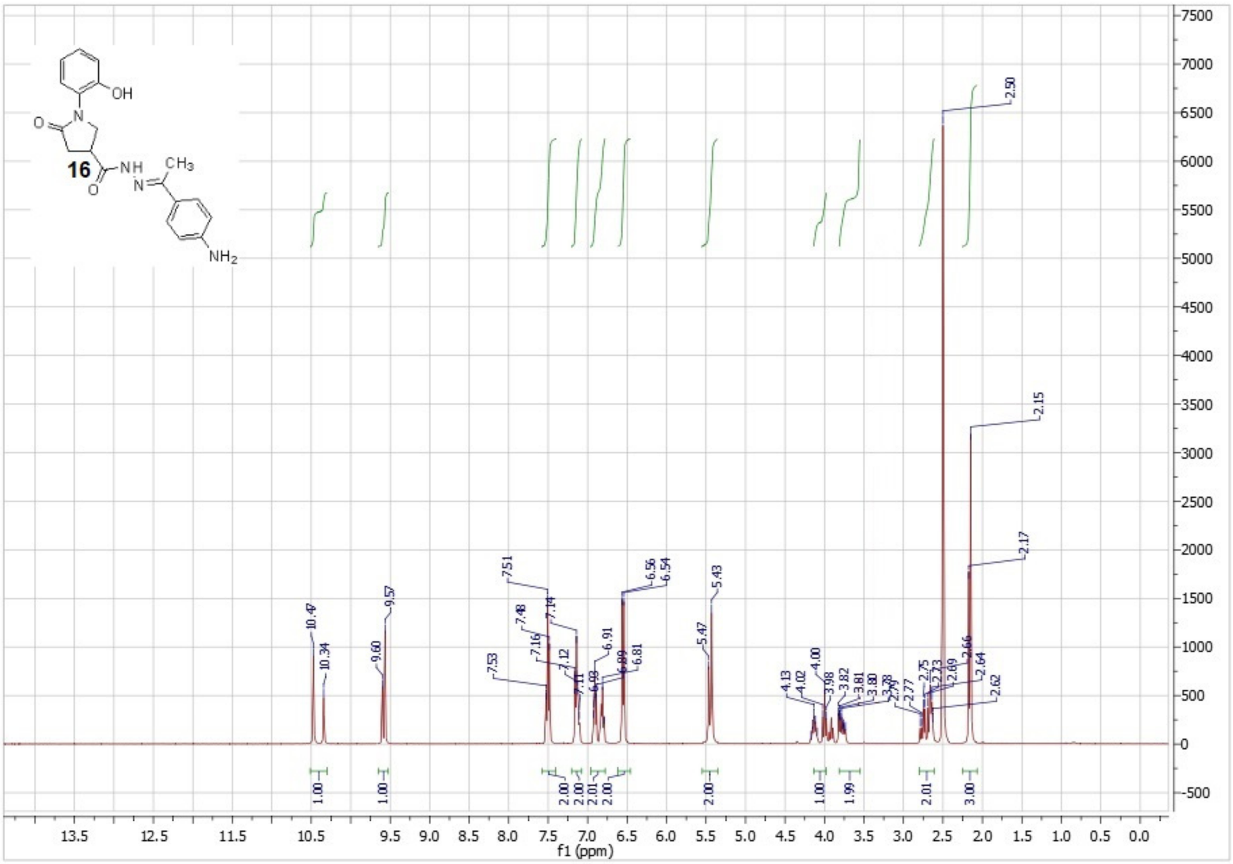

Figure S23. ^{1}H NMR spectrum of compound **16.**

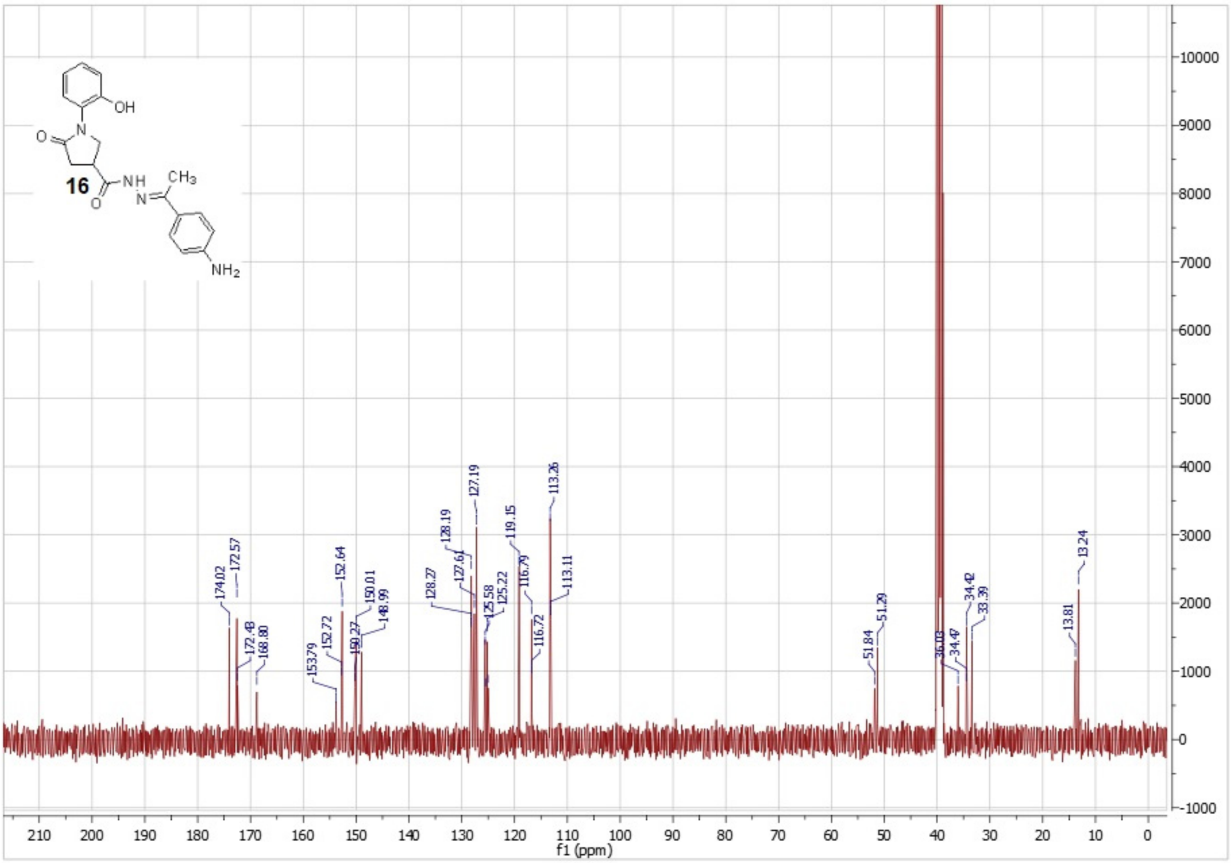

Figure S24. ^{13}C NMR spectrum of compound **16.**

1-(2-Hydroxyphenyl)-5-oxo-*N*'-(2-oxoindolin-3-ylidene)pyrrolidine-3-carbohydrazide (17)

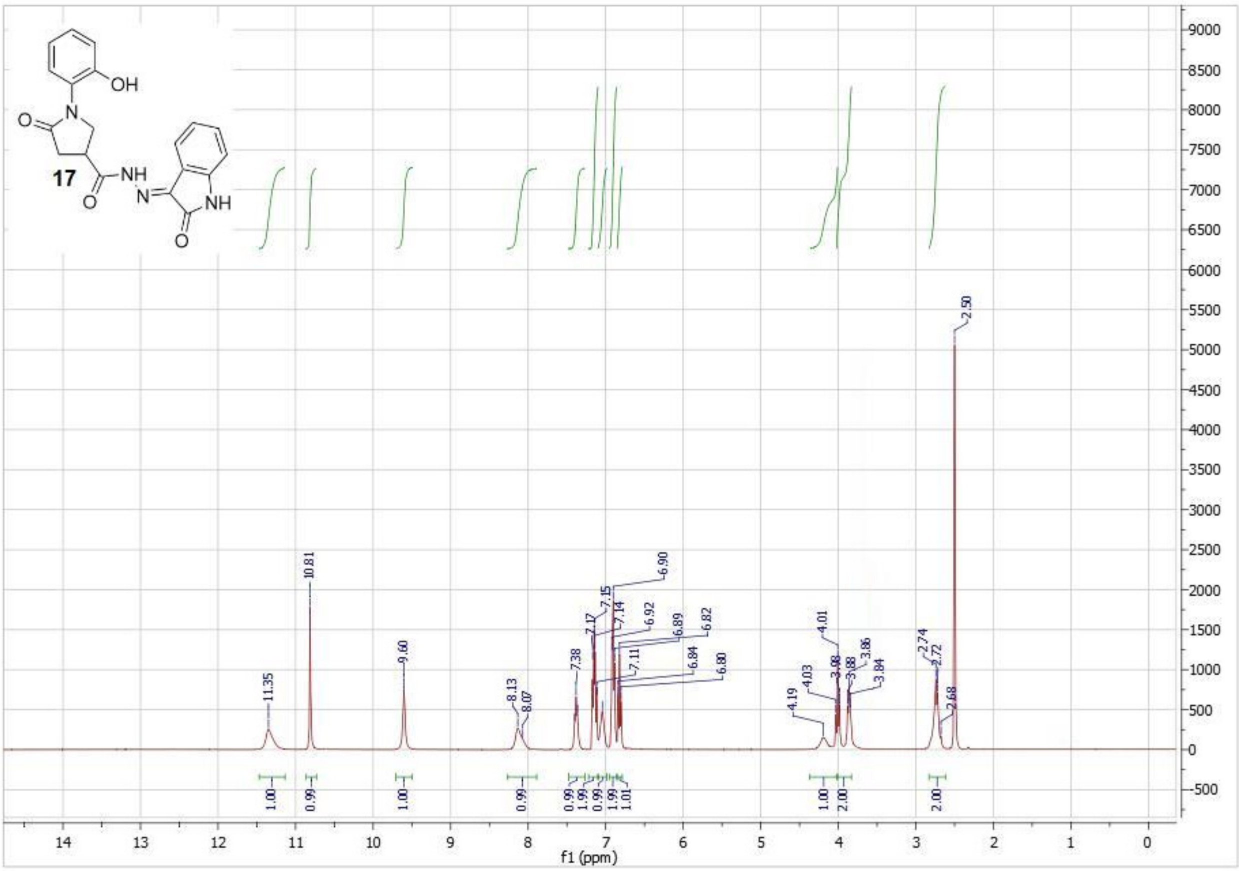

Figure S25. ^{1}H NMR spectrum of compound **17.**

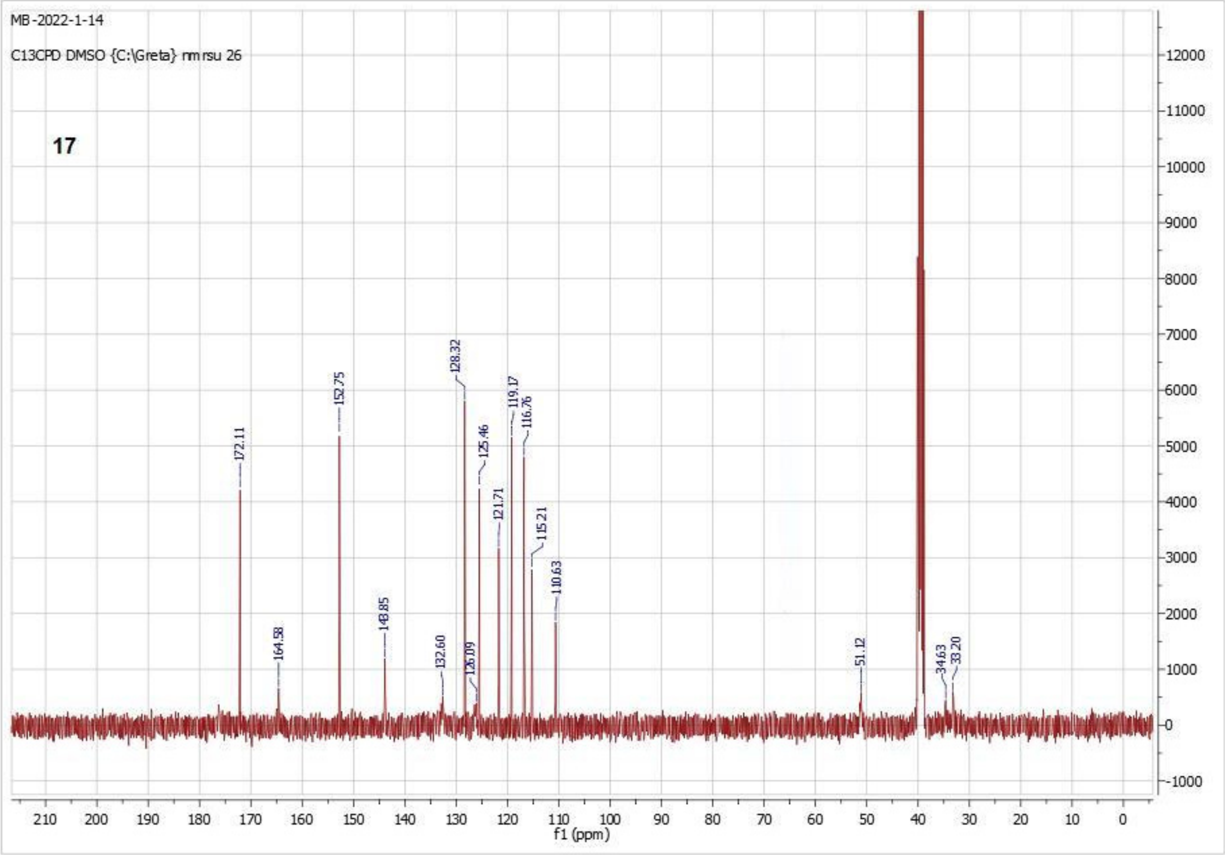

Figure S26. ^{13}C NMR spectrum of compound **17.**

1-(2-Hydroxyphenyl)-*N*-(2,5-dimethyl-1*H*-pyrrol-1-yl)-5-oxopyrrolidine-3-carboxamide (18)

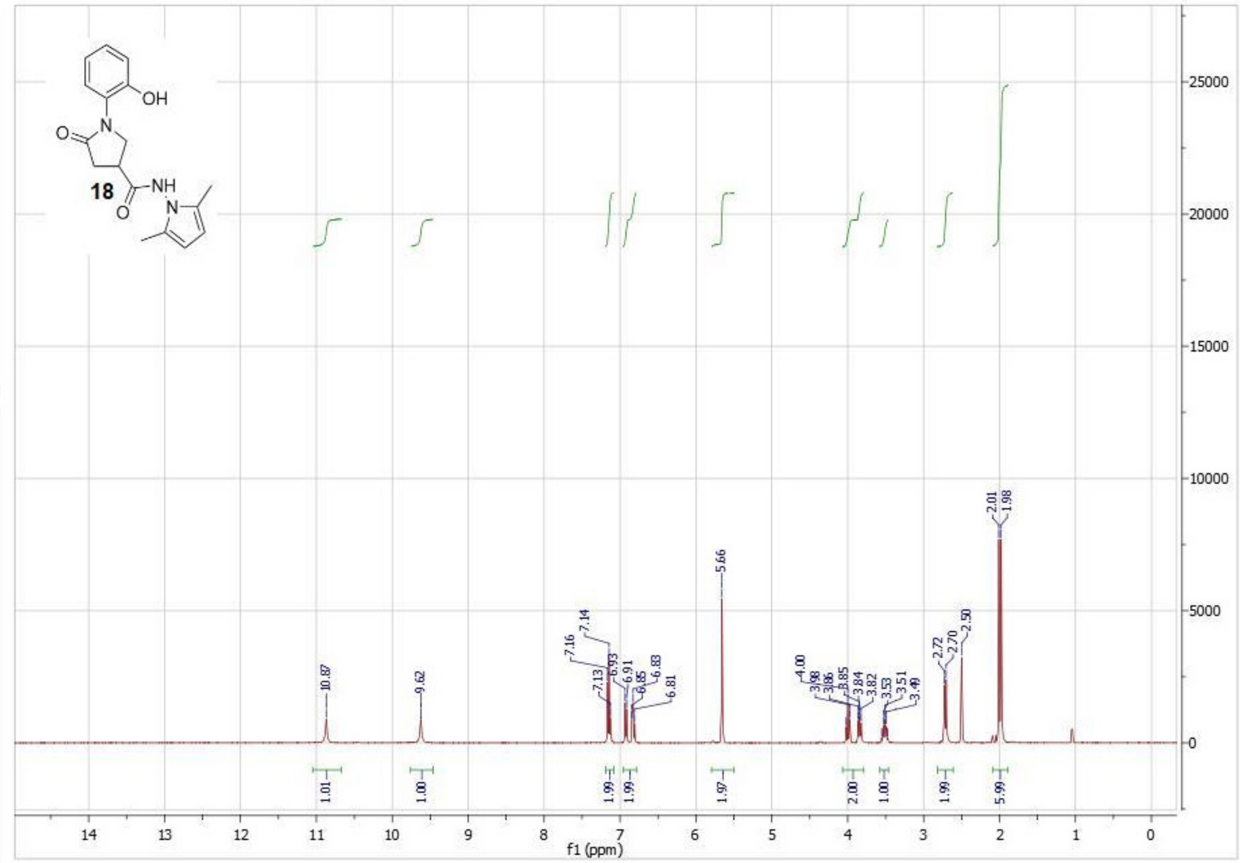

Figure S27. ^{1}H NMR spectrum of compound **18.**

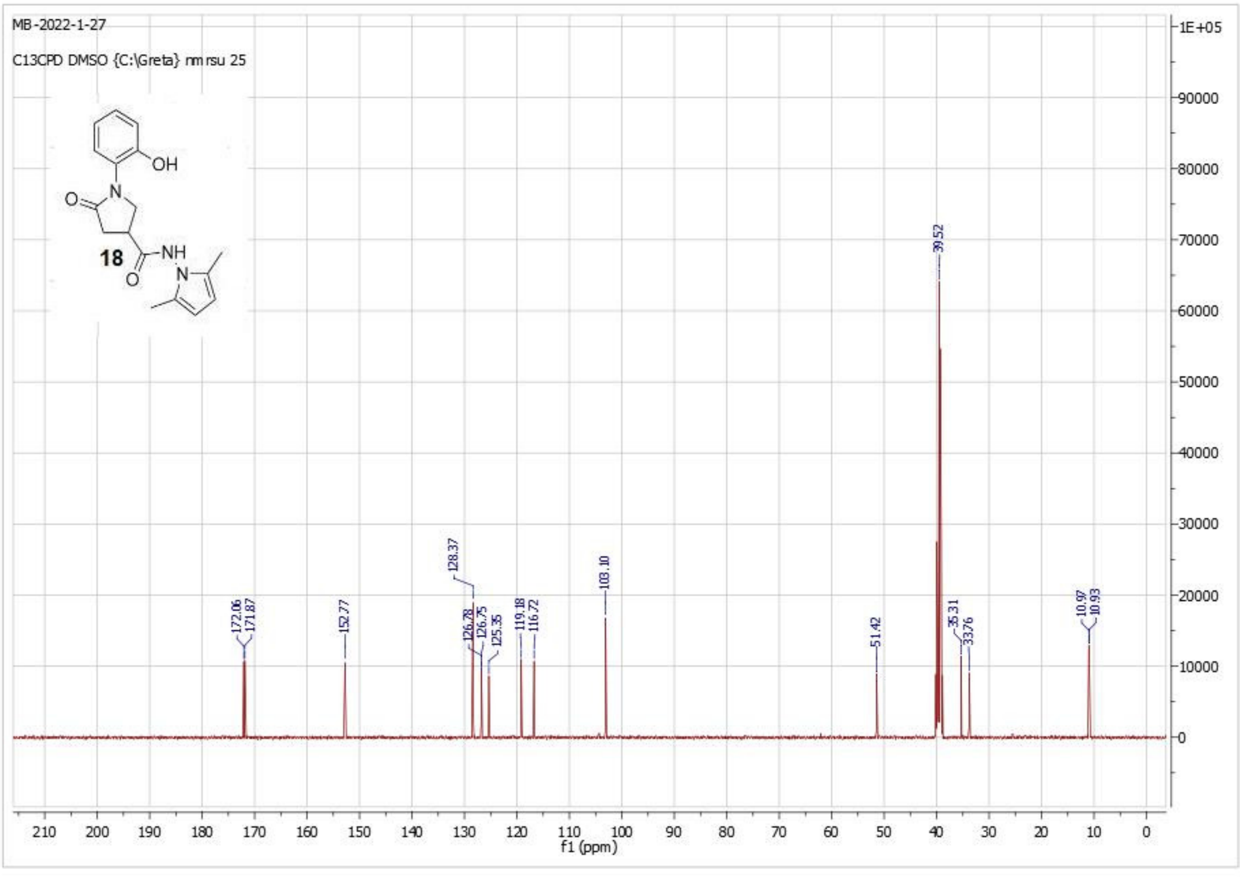

Figure S28. ^{13}C NMR spectrum of compound **18.**

1-(2-Hydroxyphenyl)-4-(3,5-dimethyl-1*H*-pyrazol-1-carbonyl)pyrrolidin-2-one (19).

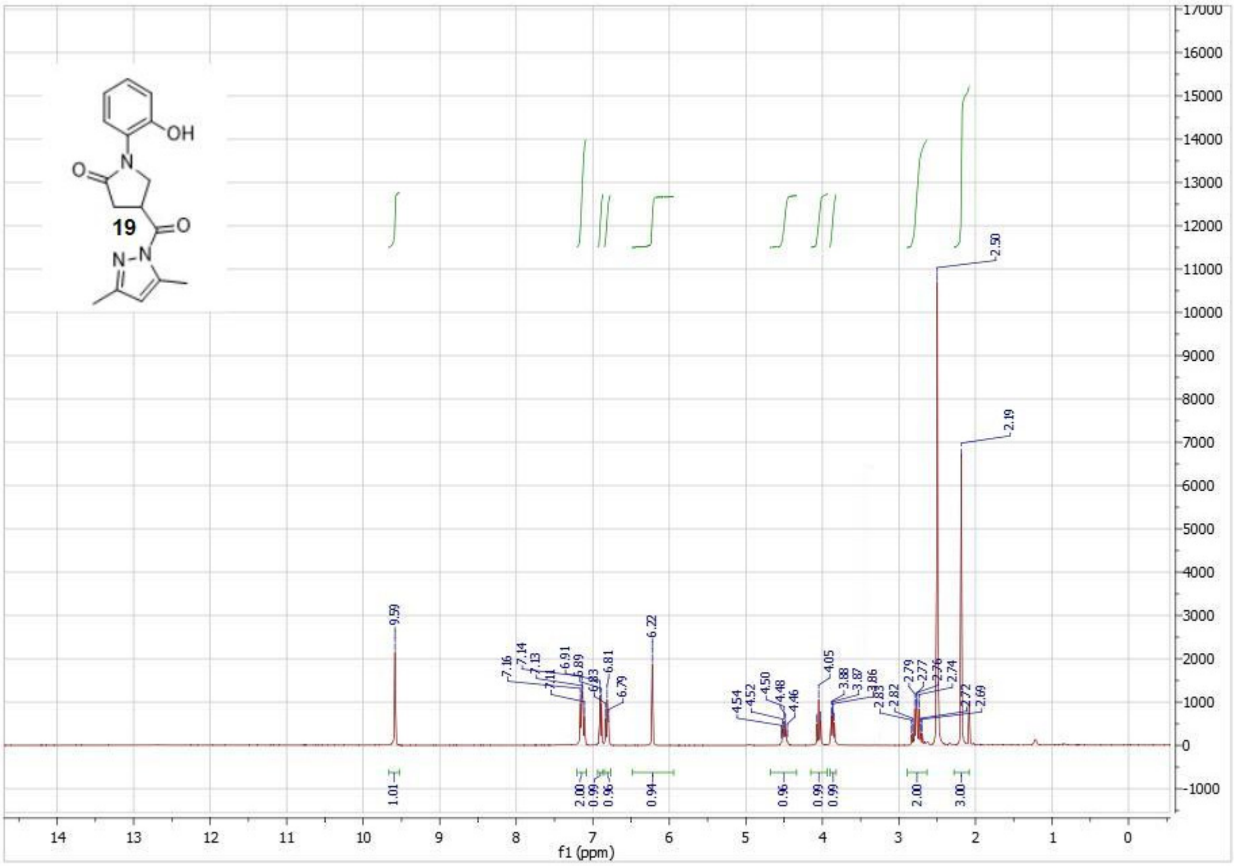

Figure S29. ^{1}H NMR spectrum of compound **19.**

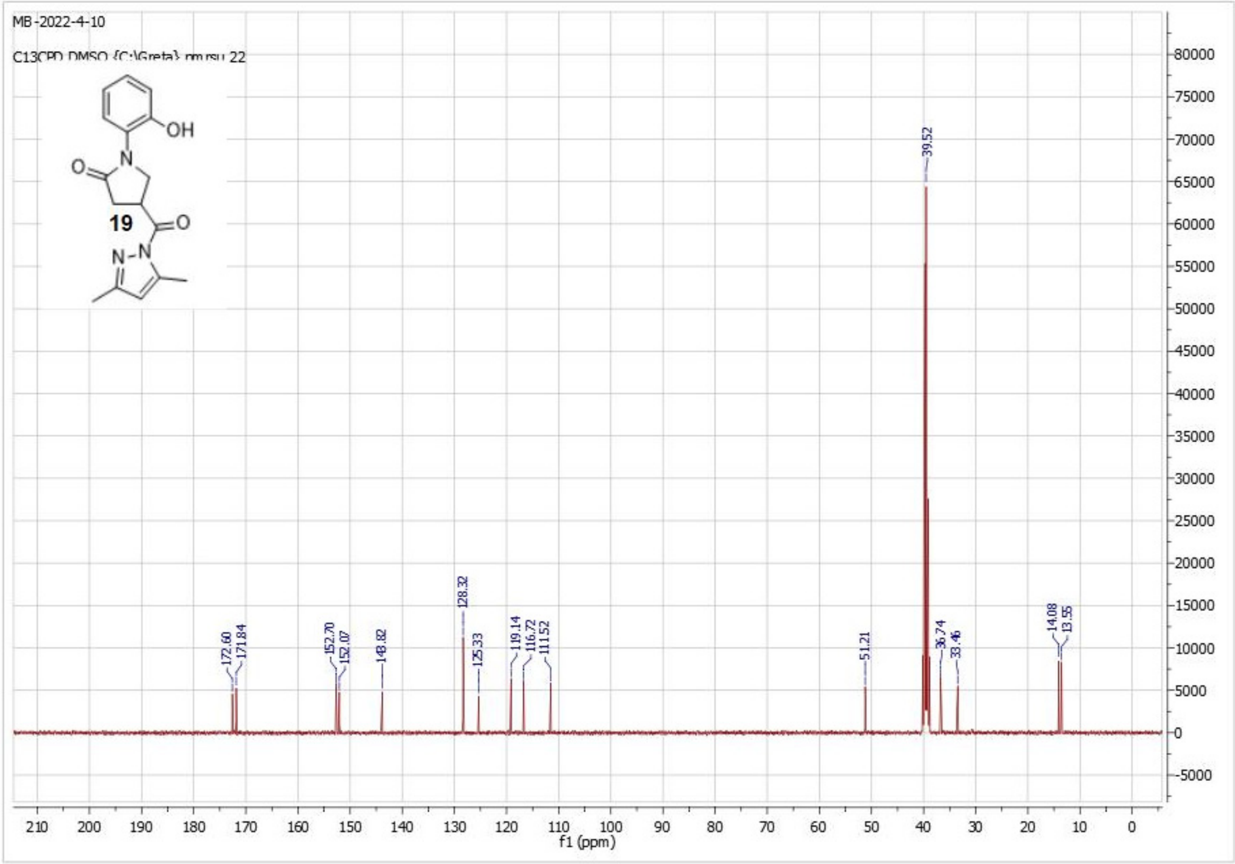

Figure S30. ^{13}C NMR spectrum of compound **19.**

1-(2-Hydroxyphenyl)-4-(5,6-diphenyl-1,2,4-triazin-3-yl)pyrrolidine-2-one (20).

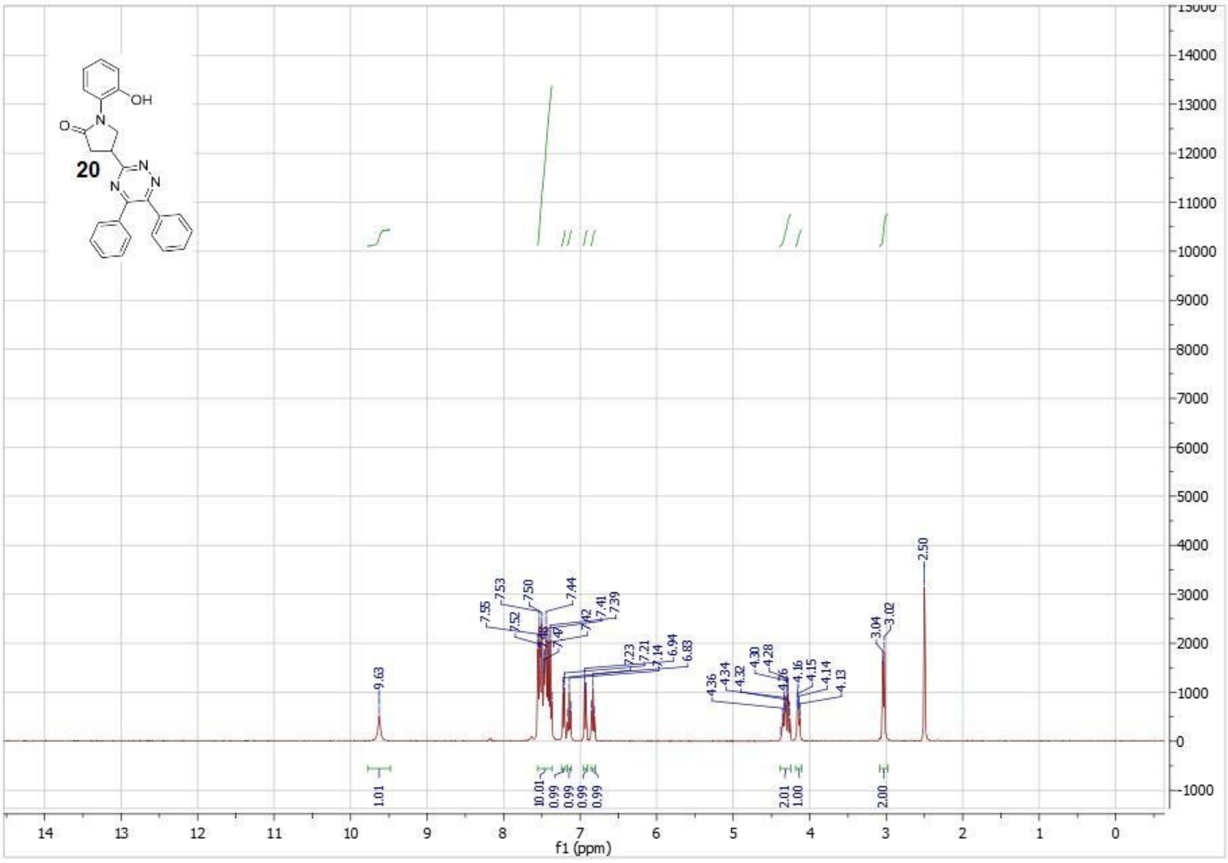

Figure S31. ^{1}H NMR spectrum of compound **20.**

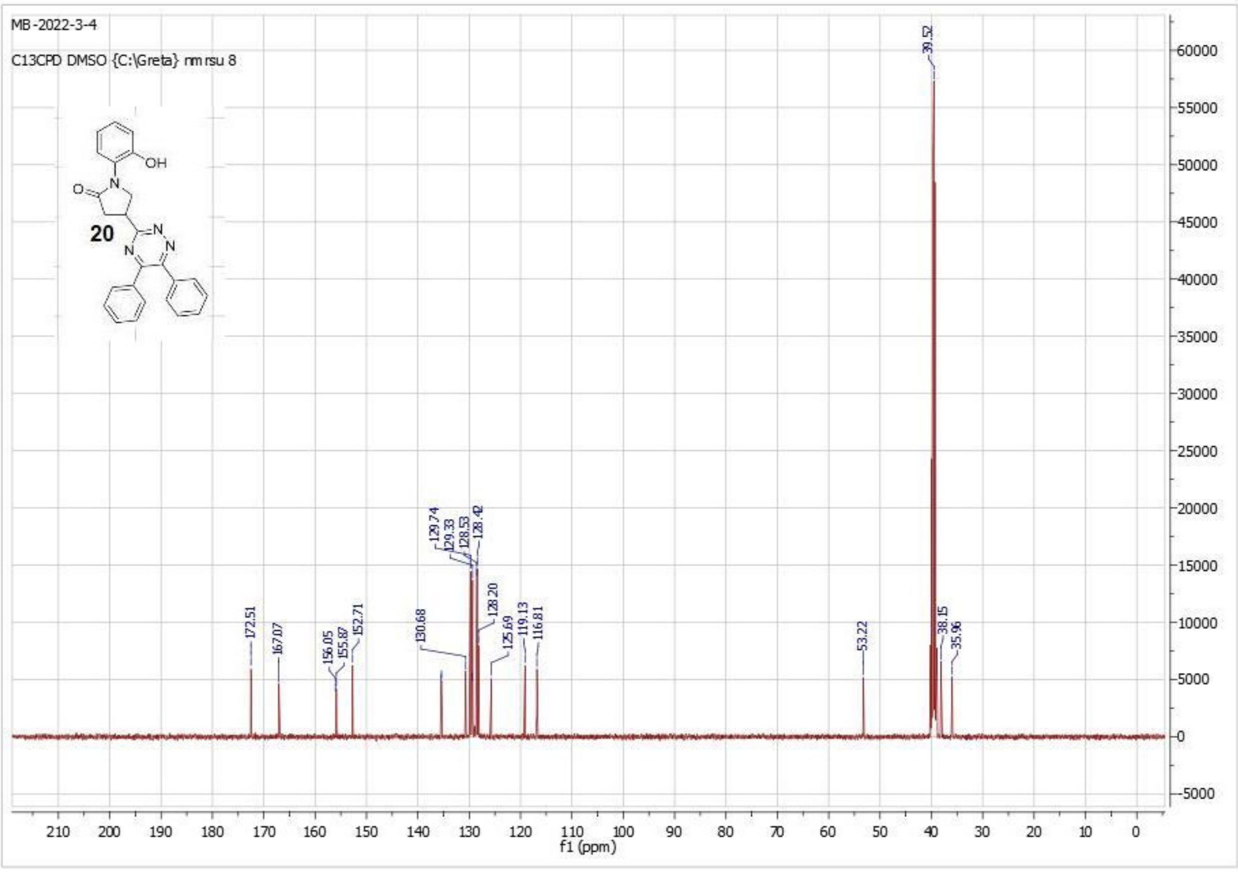

Figure S32. ^{13}C NMR spectrum of compound **20.**

4-(1*H*-benzo[d]imidazol-2-yl)-1-(2-hydroxyphenyl)pyrrolidine-2-one (21a)

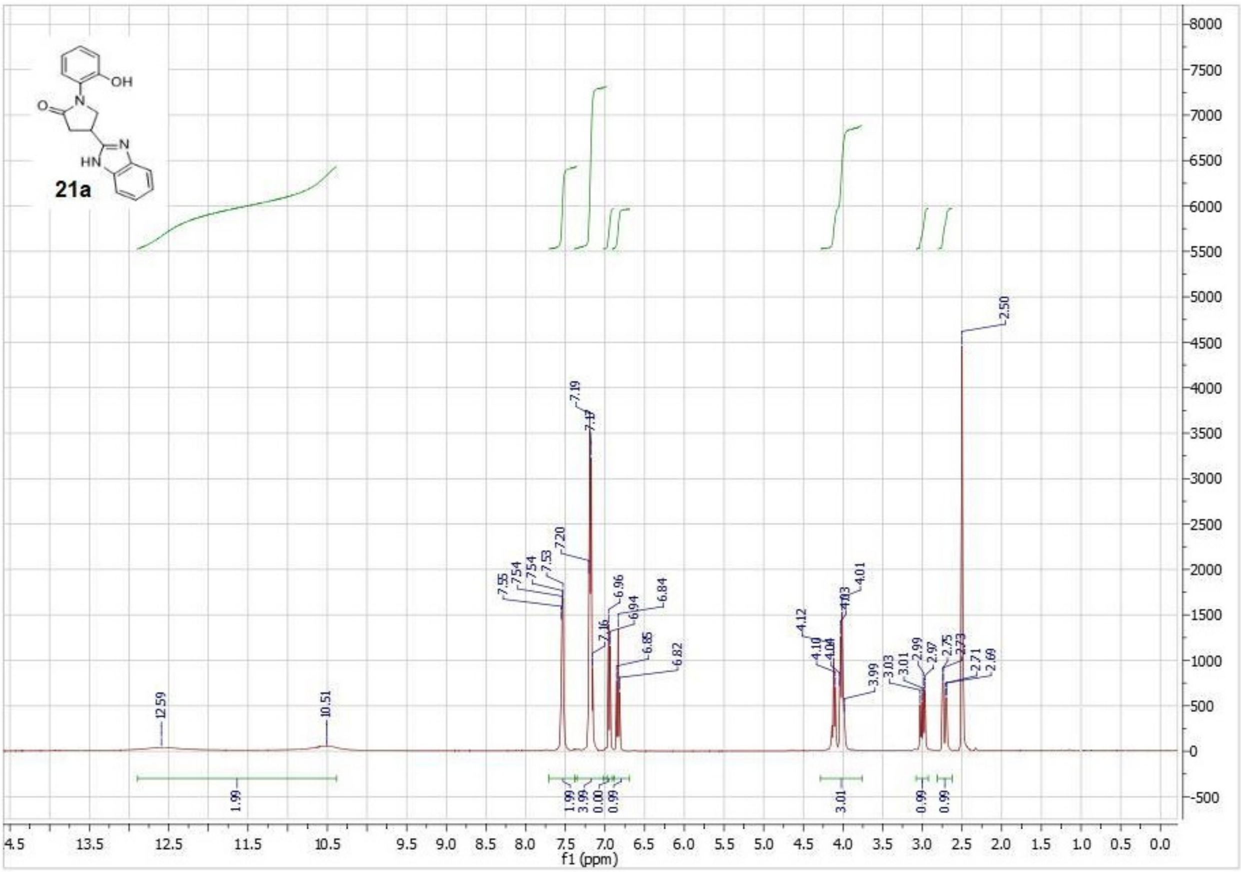

Figure S33. ^{1}H NMR spectrum of compound **21a.**

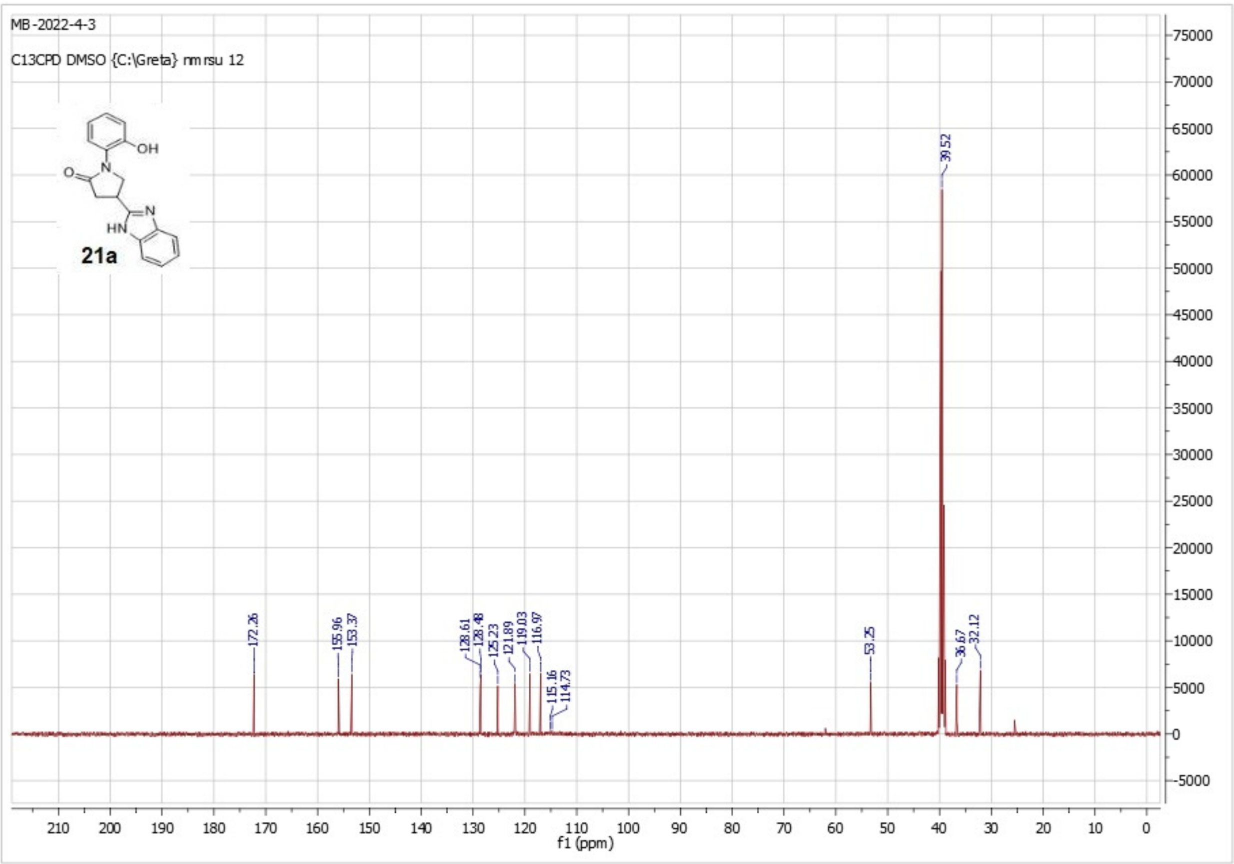

Figure S34. ^{13}C NMR spectrum of compound **21a.**

4-(1*H*-benzo[d]imidazol-2-yl)-1-(3,5-dichloro-2-hydroxyphenyl)pyrrolidin-2-one (21b)

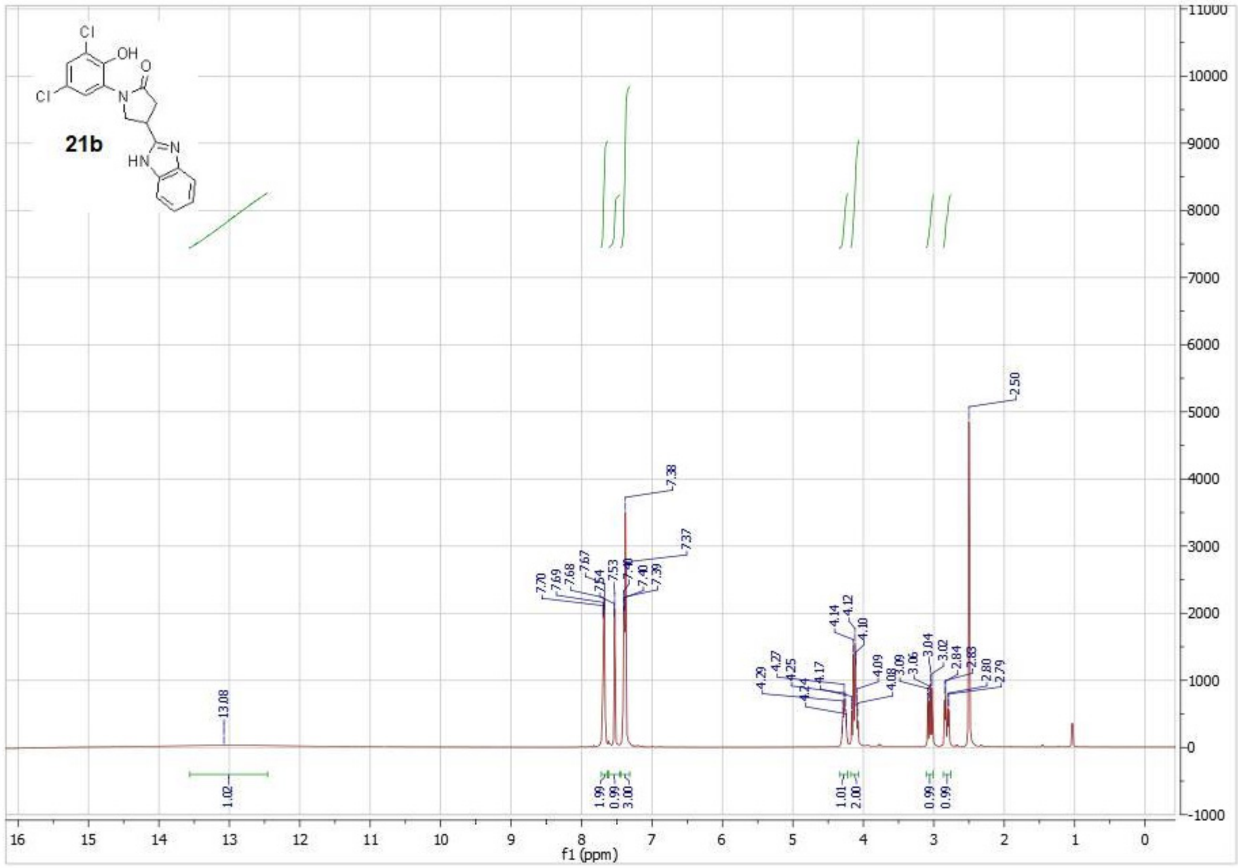

Figure S35. ^{1}H NMR spectrum of compound **21b.**

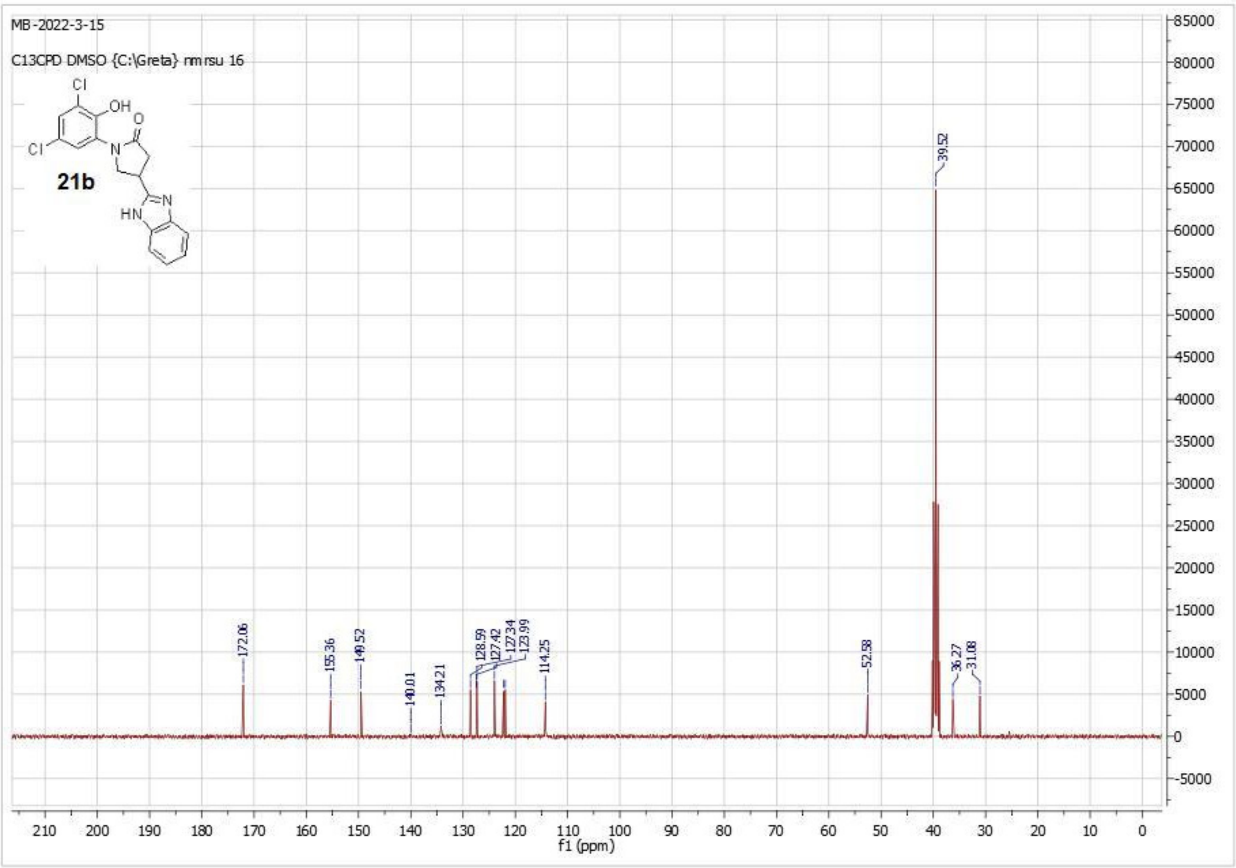

Figure S36. ^{13}C NMR spectrum of compound **21b.**

4-(5-Methyl-1*H*-benzo[d]imidazol-2-yl)-1-(2-hydroxyphenyl)pyrrolidine-2-one (22a)

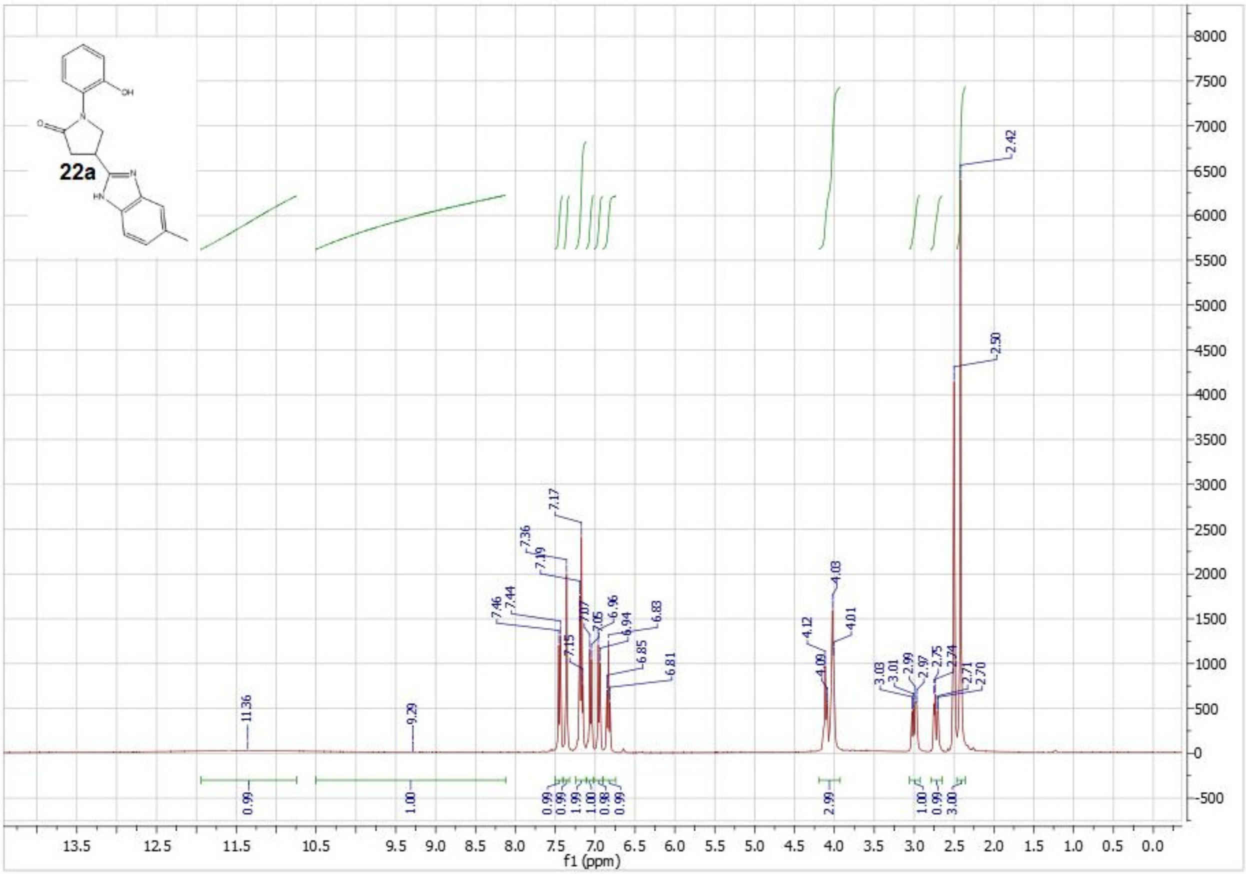

Figure S37. ^{1}H NMR spectrum of compound **22a.**

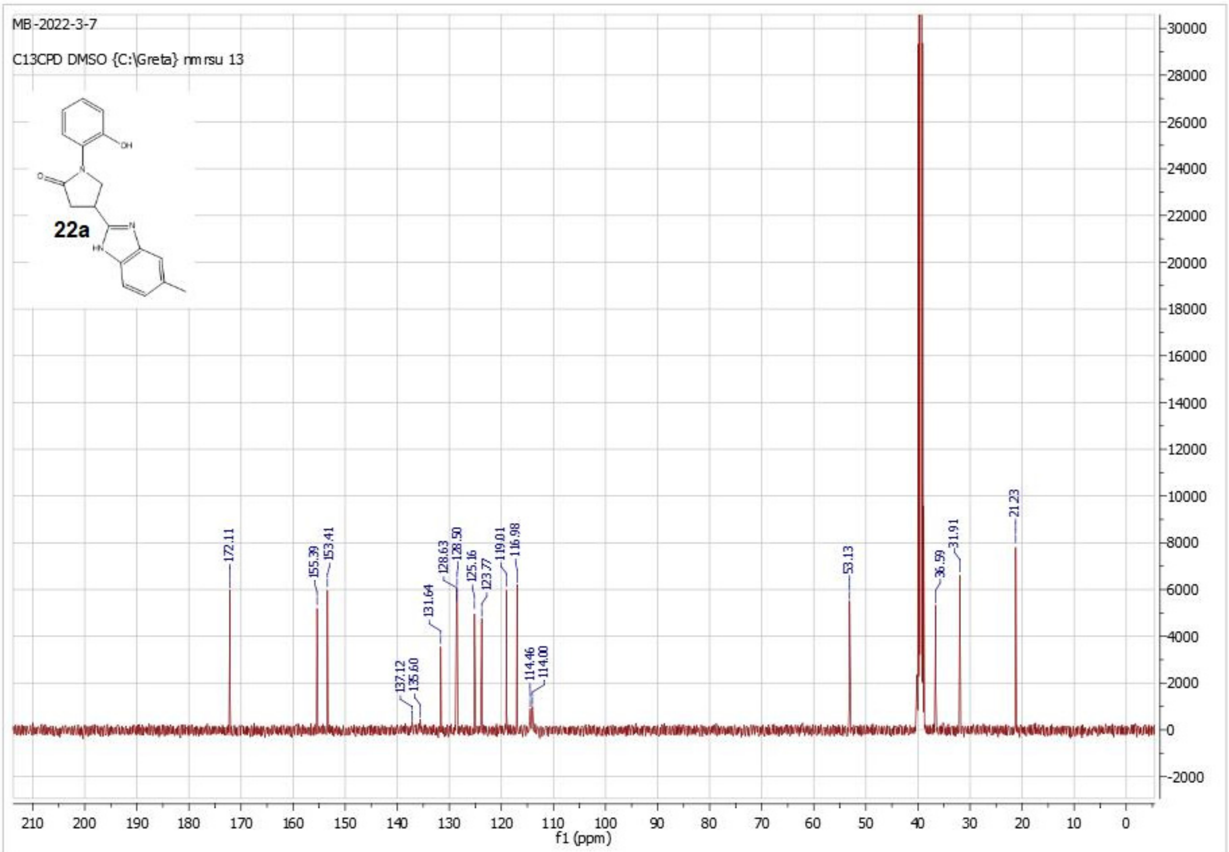

Figure S38. ^{13}C NMR spectrum of compound **22a.**

1-(3,5-Dichloro-2-hydroxyphenyl)-4-(6-methyl-1*H*-benzo[d]imidazol-2-yl)pyrrolidin-2-one (22b)

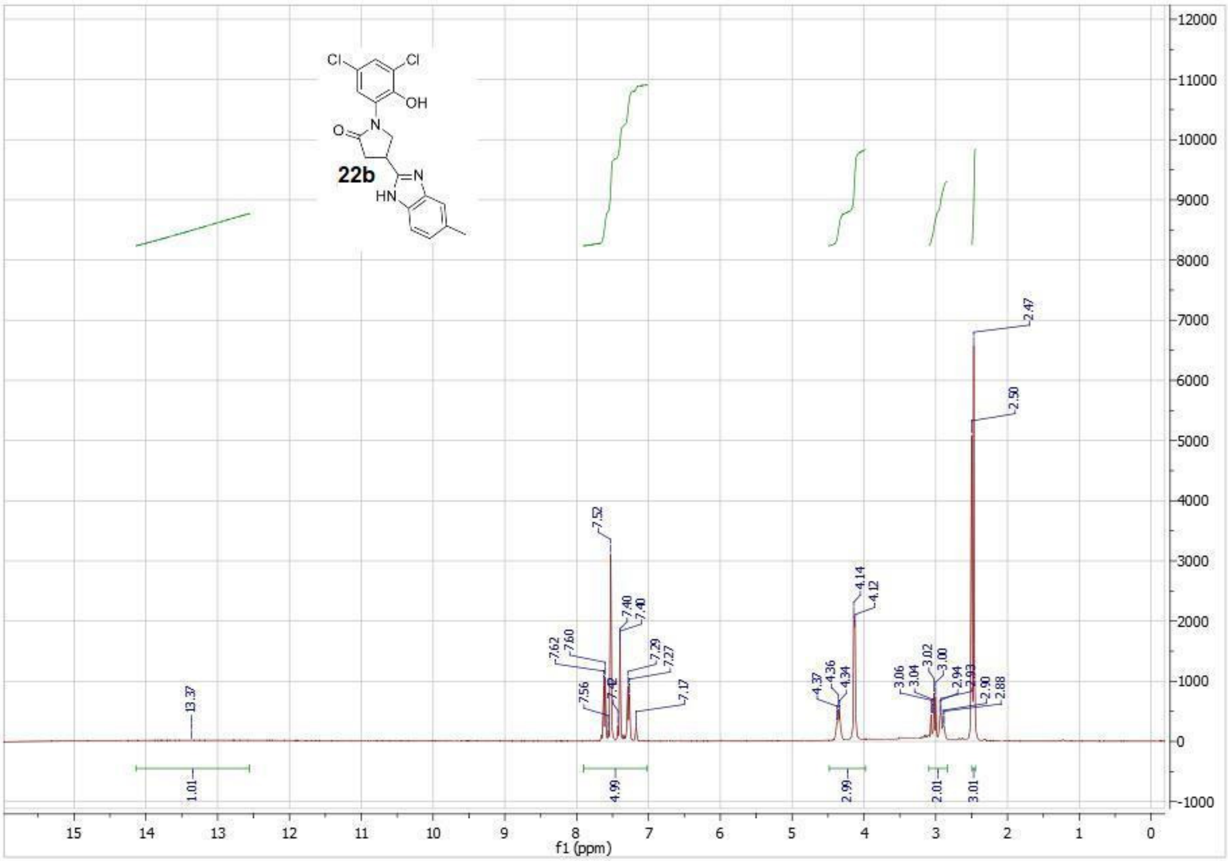

Figure S39. ^{1}H NMR spectrum of compound **22b.**

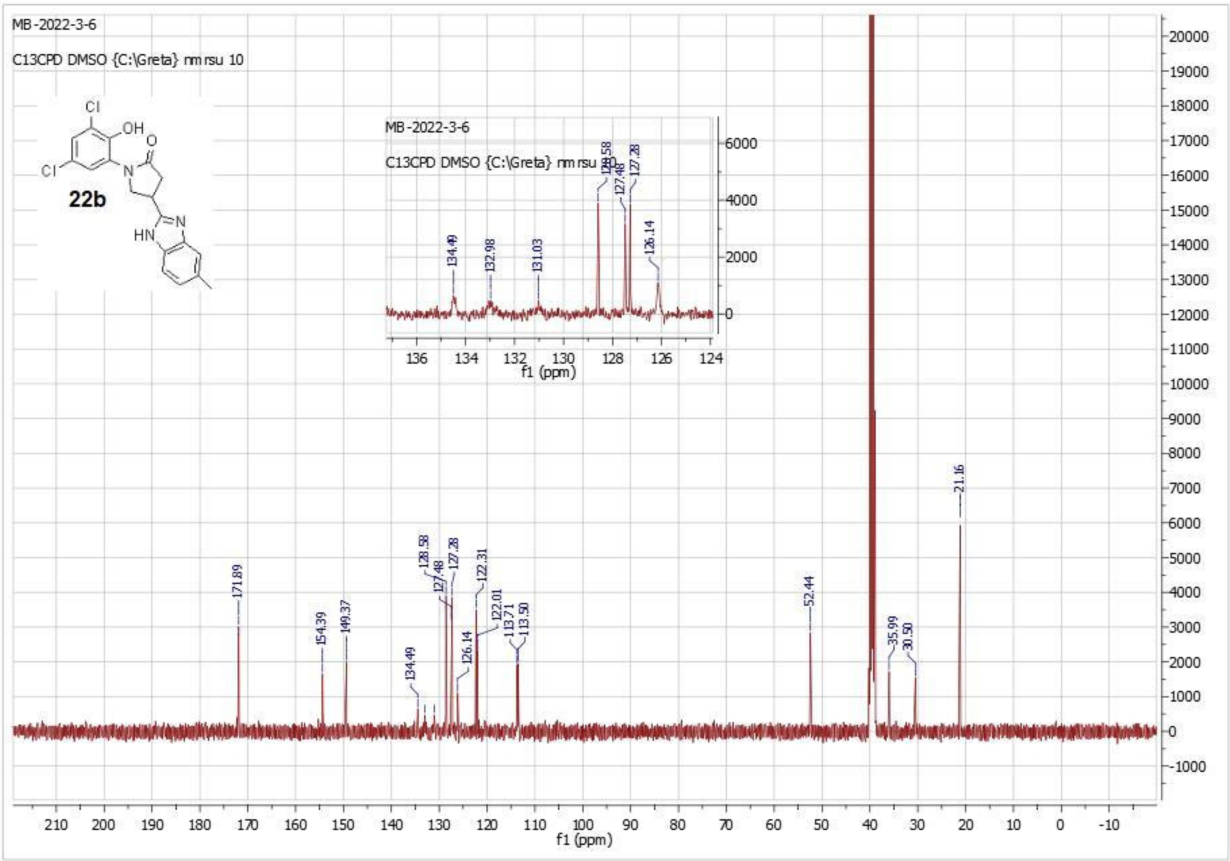

Figure S40. ^{13}C NMR spectrum of compound **22b.**

4-(5-Chloro-1*H*-benzo[d]imidazol-2-yl)-1-(2-hydroxyphenyl)pyrrolidine-2-one (23a)

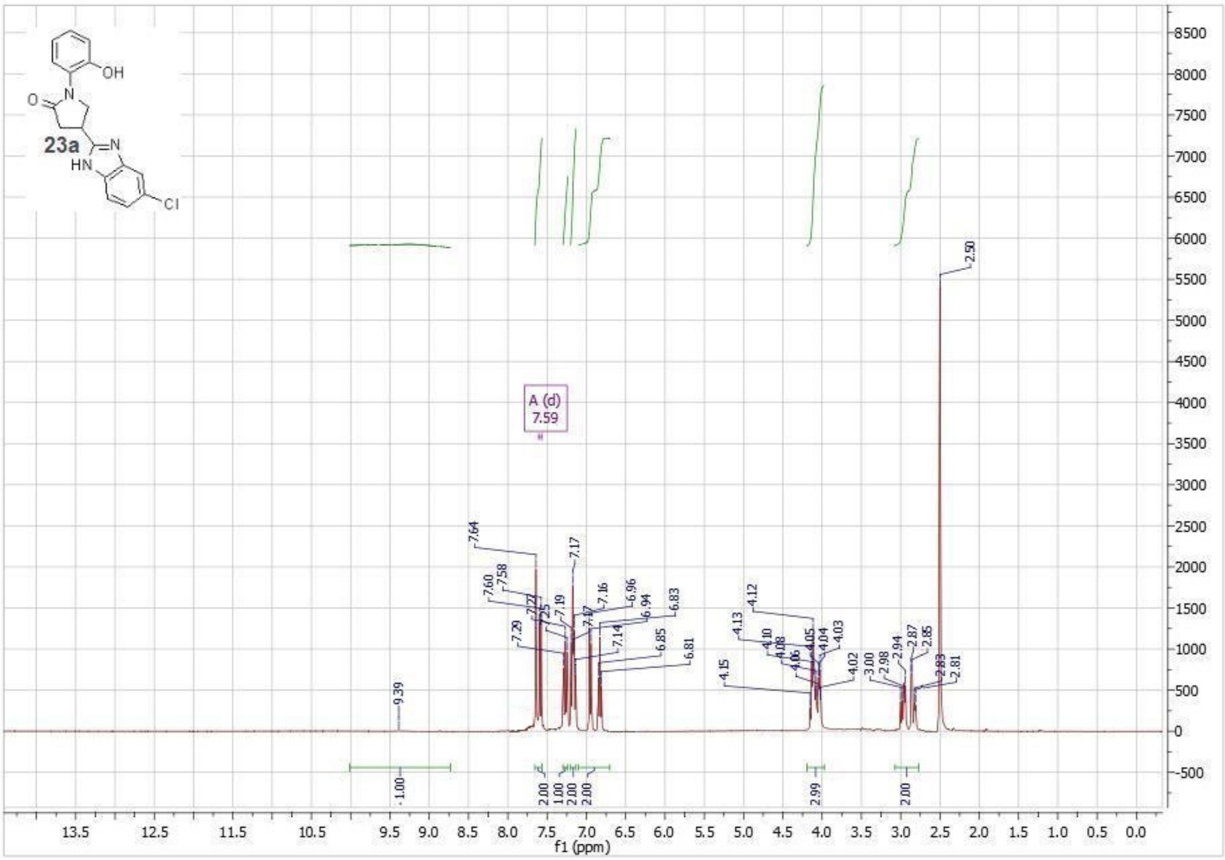

Figure S41. ^{1}H NMR spectrum of compound **23a.**

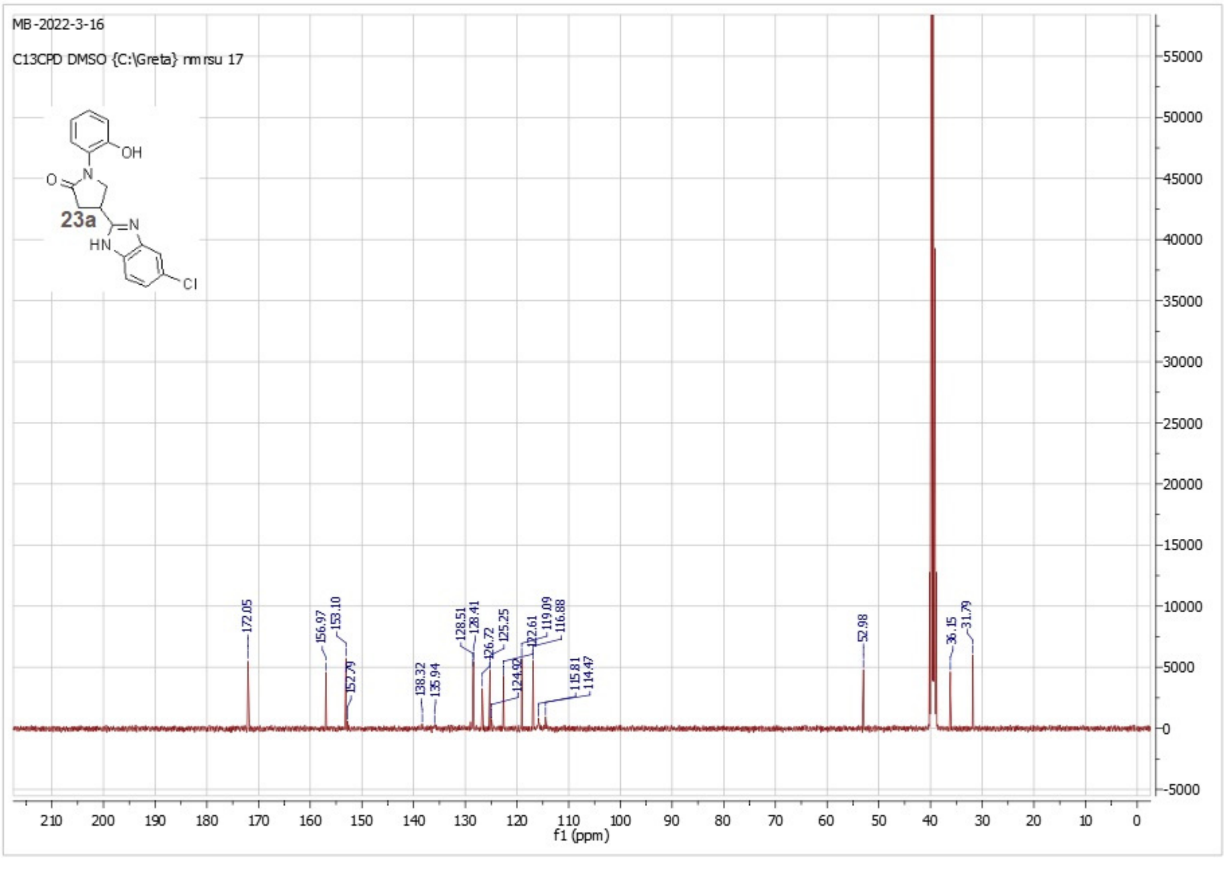

Figure S42. ^{13}C NMR spectrum of compound **23a.**

4-(6-Chloro-1*H*-benzo[d]imidazol-2-yl)-1-(3,5-dichloro-2-hydroxyphenyl)pyrrolidin-2-one (23b)

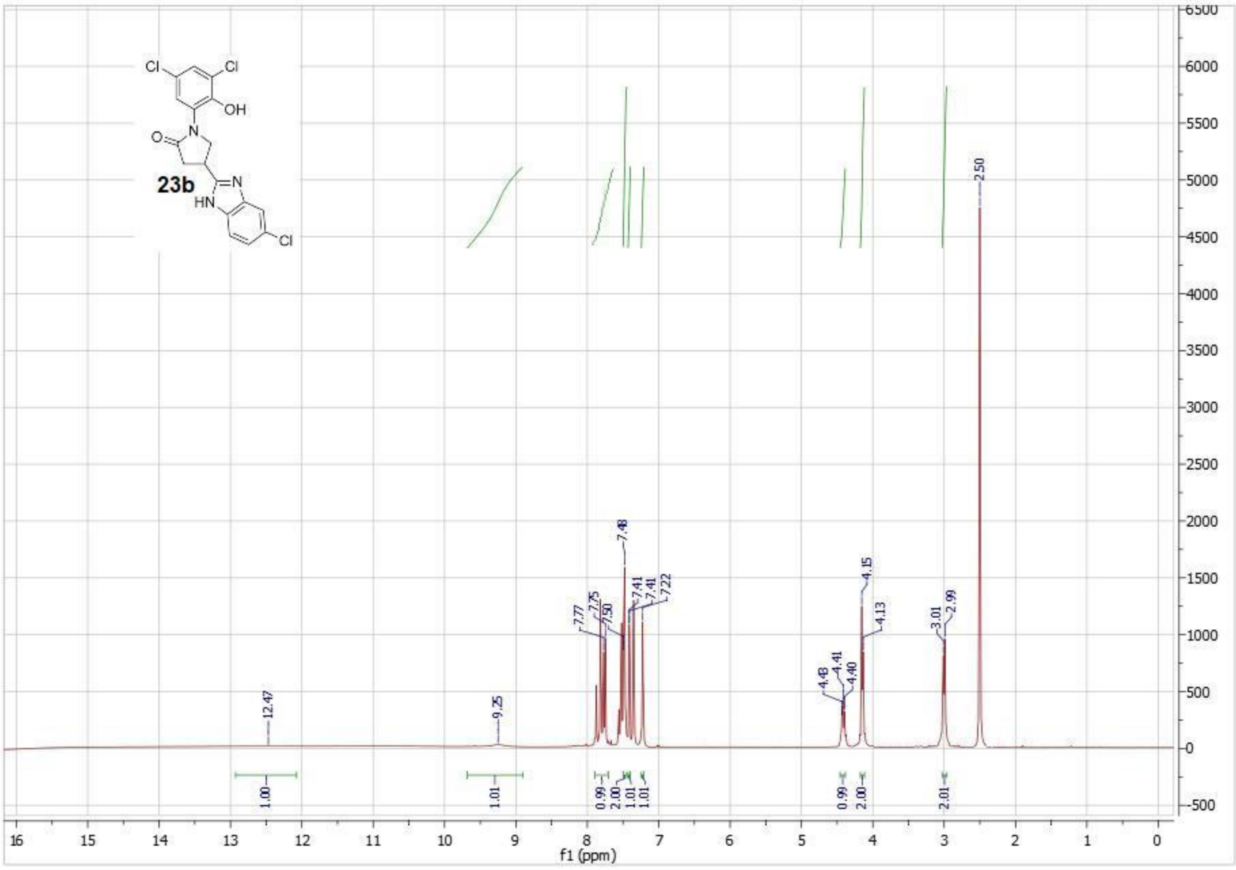

Figure S43. ^{1}H NMR spectrum of compound **23b.**

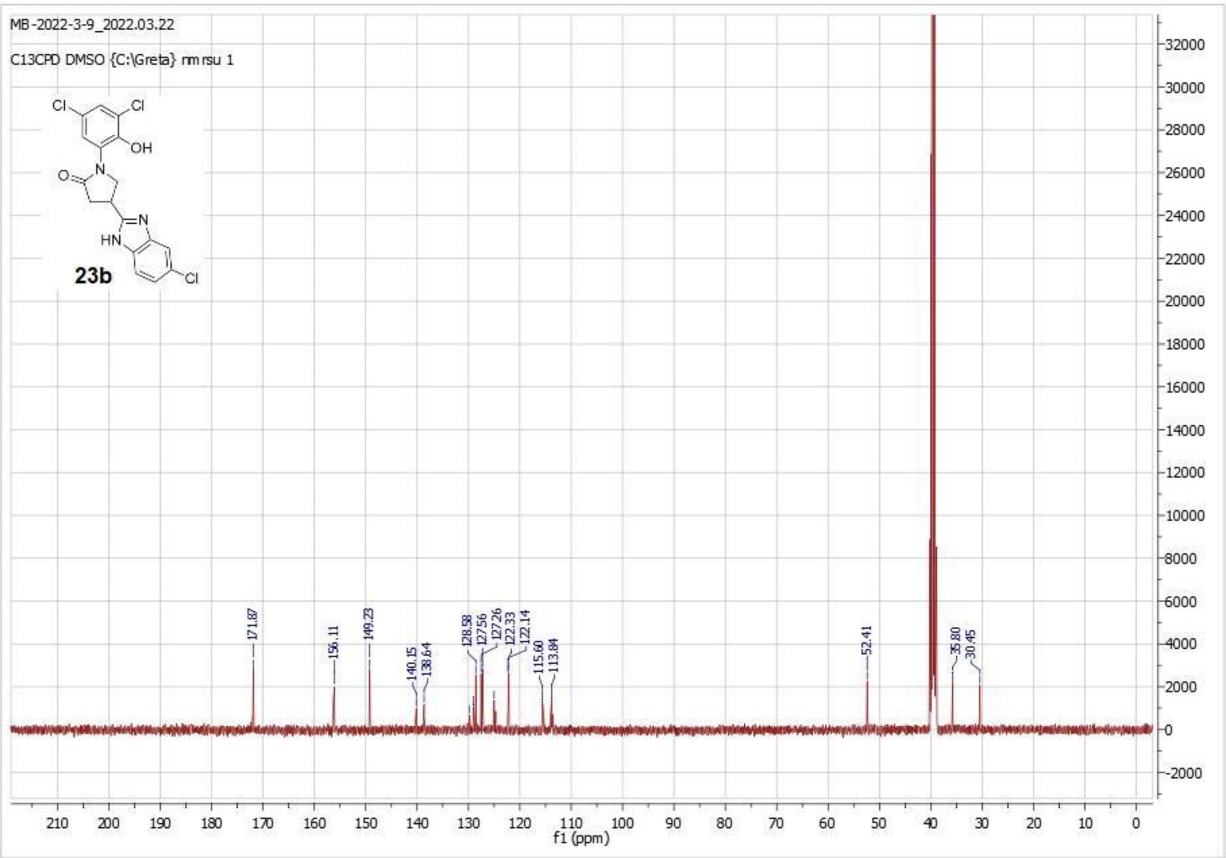

Figure S44. ^{13}C NMR spectrum of compound **23b.**

4-(5-Fluoro-1*H*-benzo[d]imidazol-2-yl)-1-(2-hydroxyphenyl)pyrrolidine-2-one (24a)

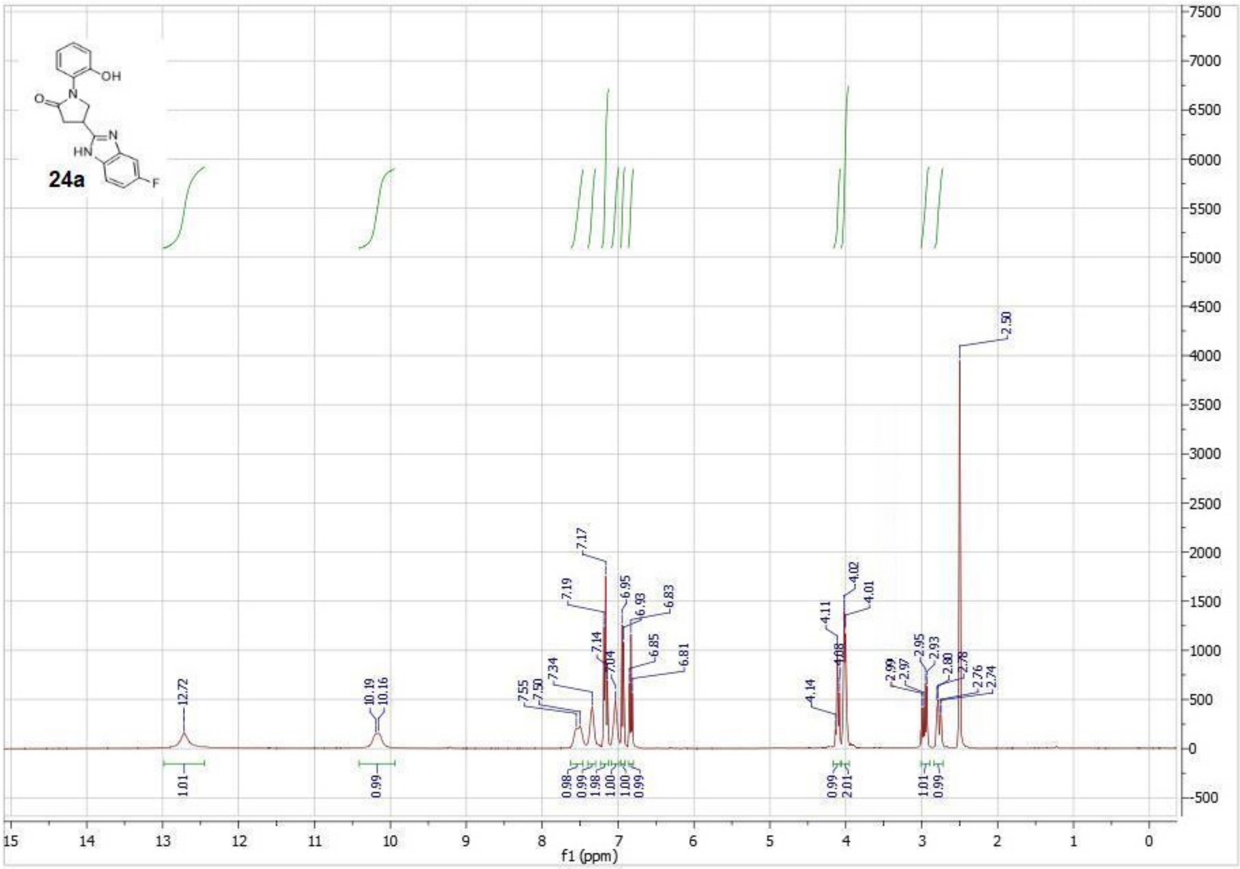

Figure S45. ^{1}H NMR spectrum of compound **24a.**

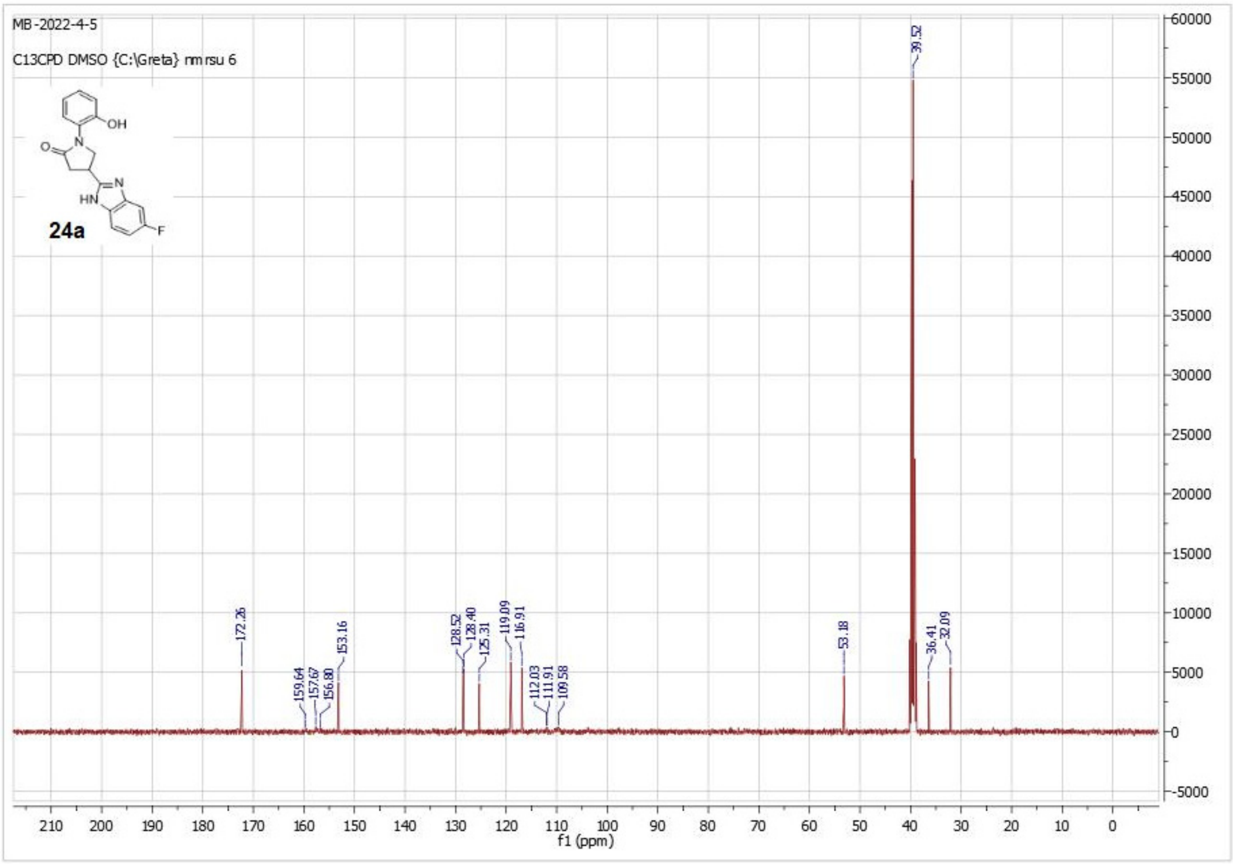

Figure S46. ^{13}C NMR spectrum of compound **24a.**

1-(3,5-Dichloro-2-hydroxyphenyl)-4-(6-fluoro-1*H*-benzo[d]imidazol-2-yl)pyrrolidin-2-one (24b)

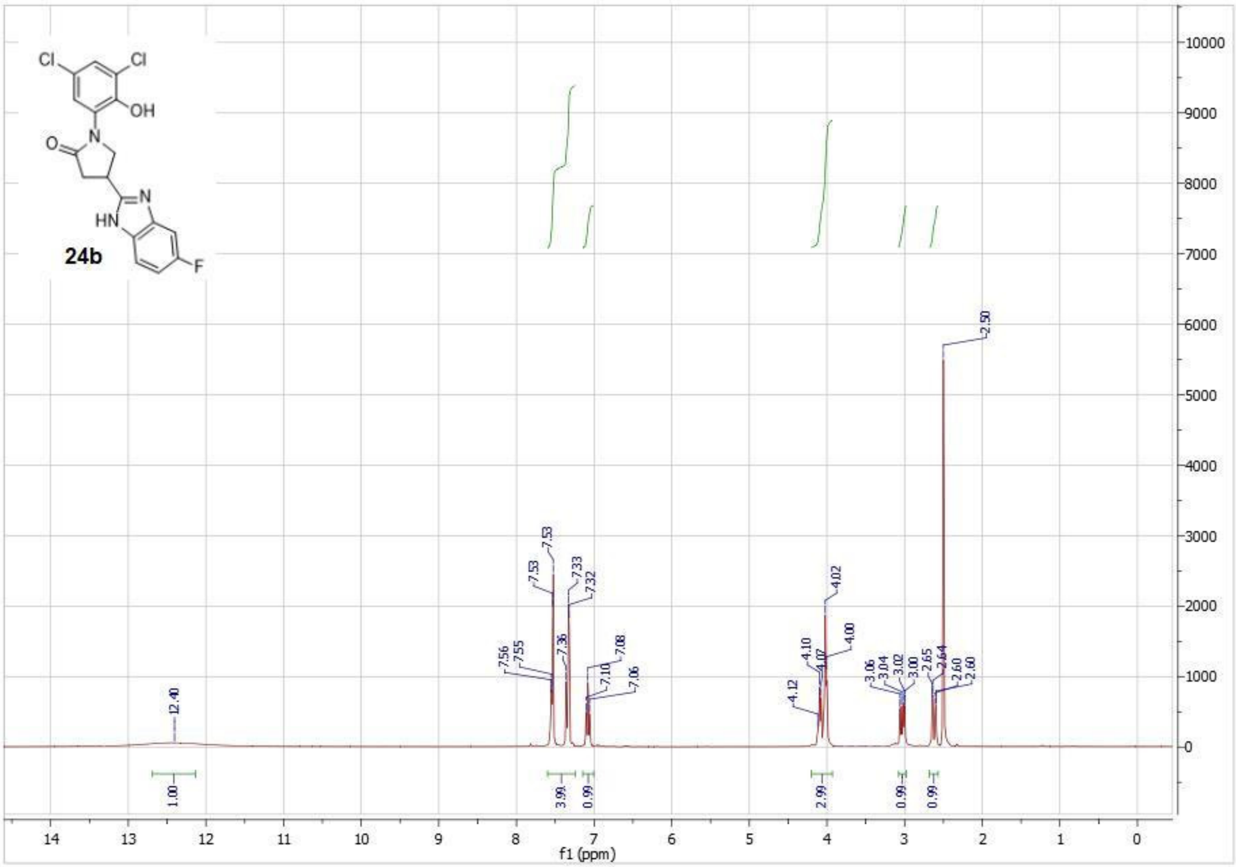

Figure S47. ^{1}H NMR spectrum of compound **24b.**

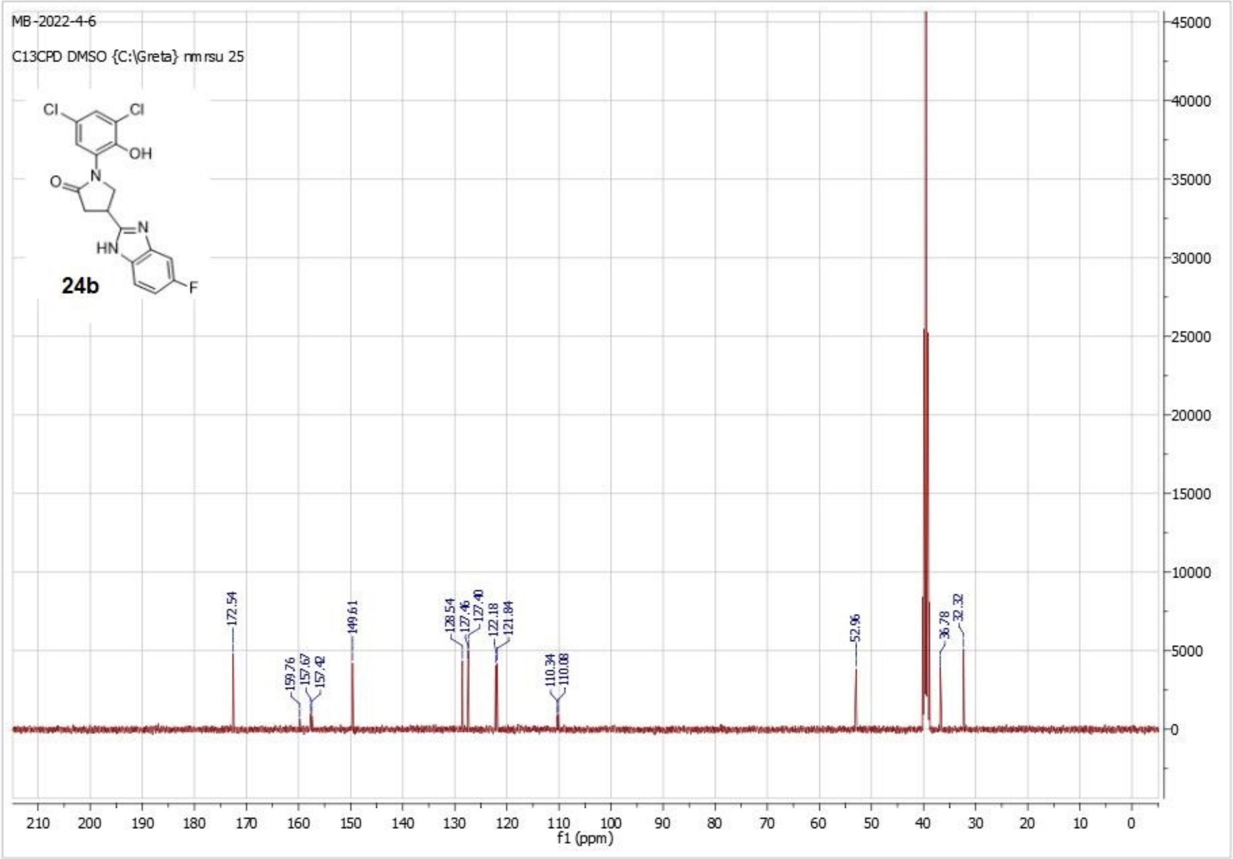

Figure S48. ^{13}C NMR spectrum of compound **24b.**

3-(1*H*-benzo[d]imidazol-2-yl)4-((2-hydroxyphenyl)amino)butanoic acid (25a)

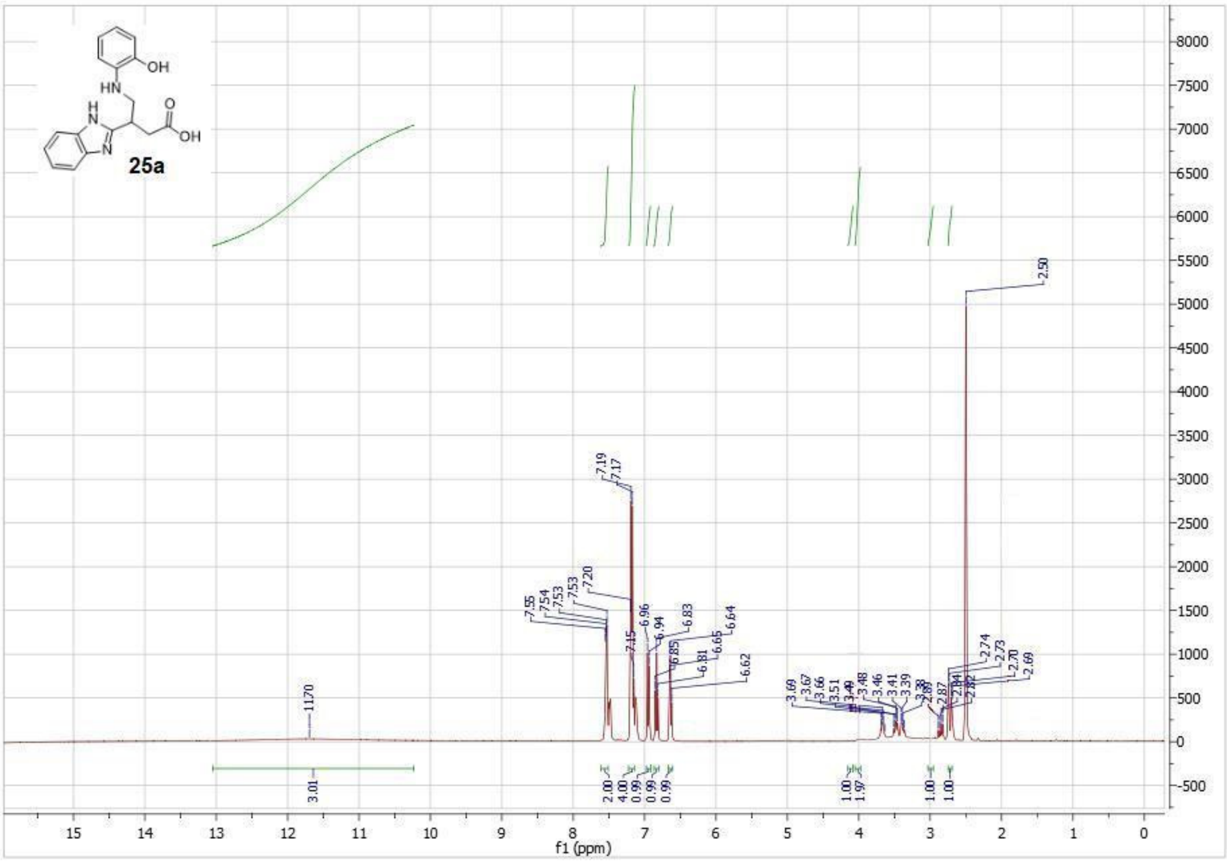

Figure S49. ^{1}H NMR spectrum of compound **25a.**

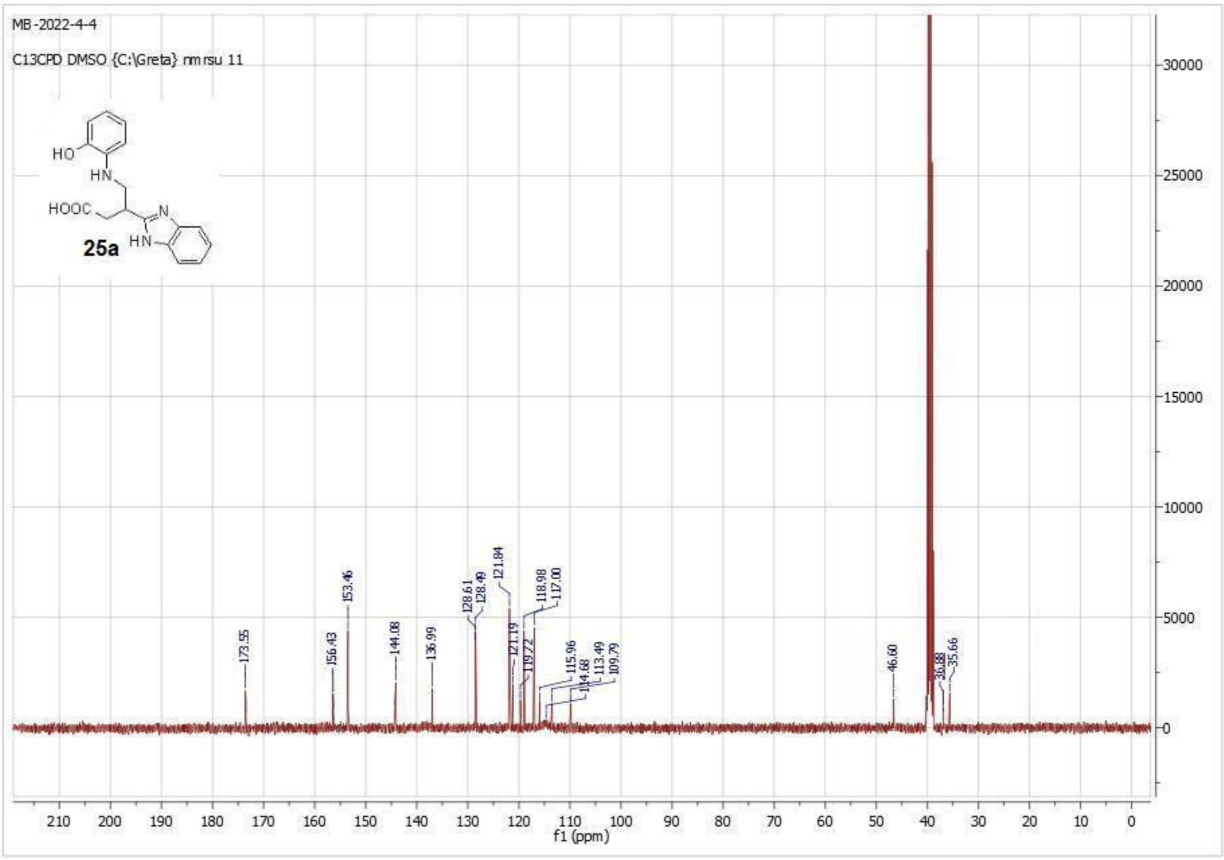

Figure S50. ^{13}C NMR spectrum of compound **25a.**

3-(1*H*-benzo[d]imidazol-2-yl)-4-((3,5-dichloro-2-hydroxyphenyl)amino)butanoic acid (25b)

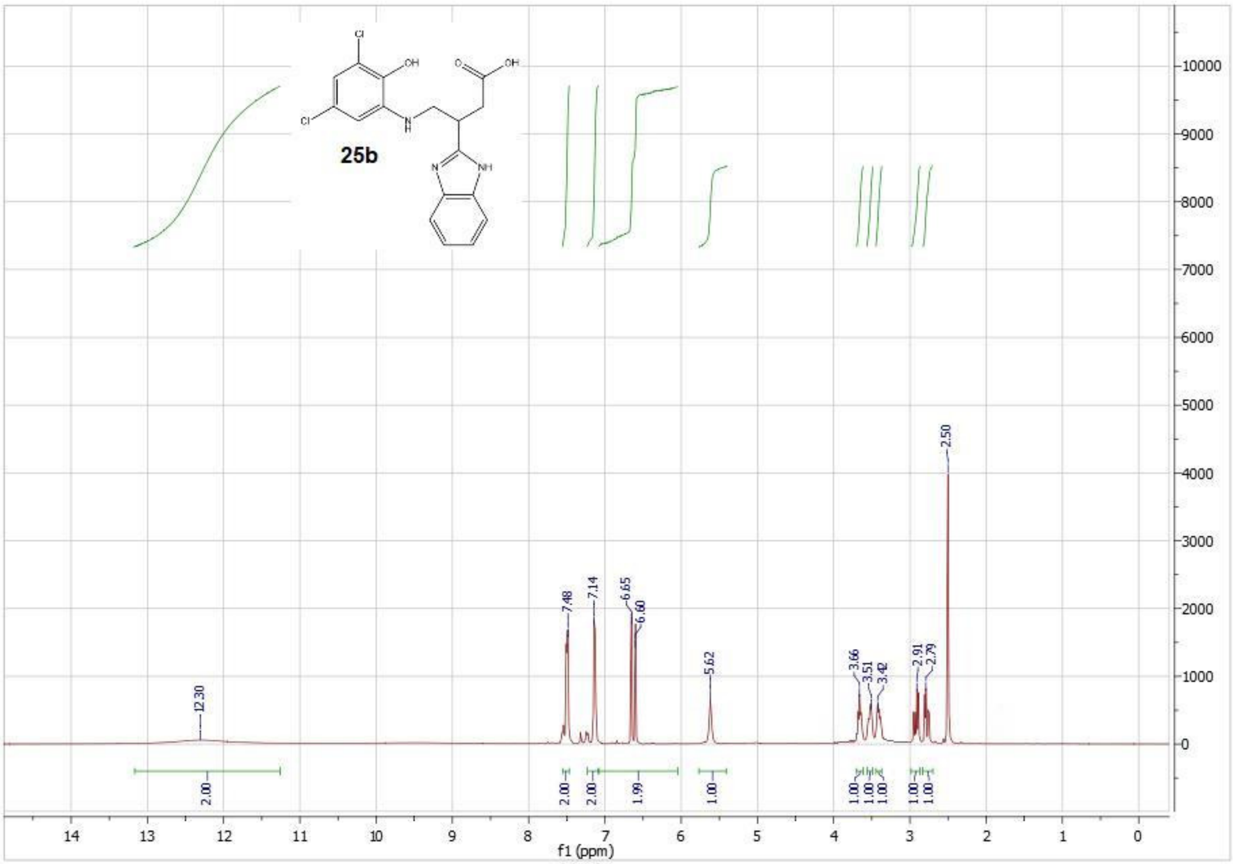

Figure S51. ^{1}H NMR spectrum of compound **25b.**

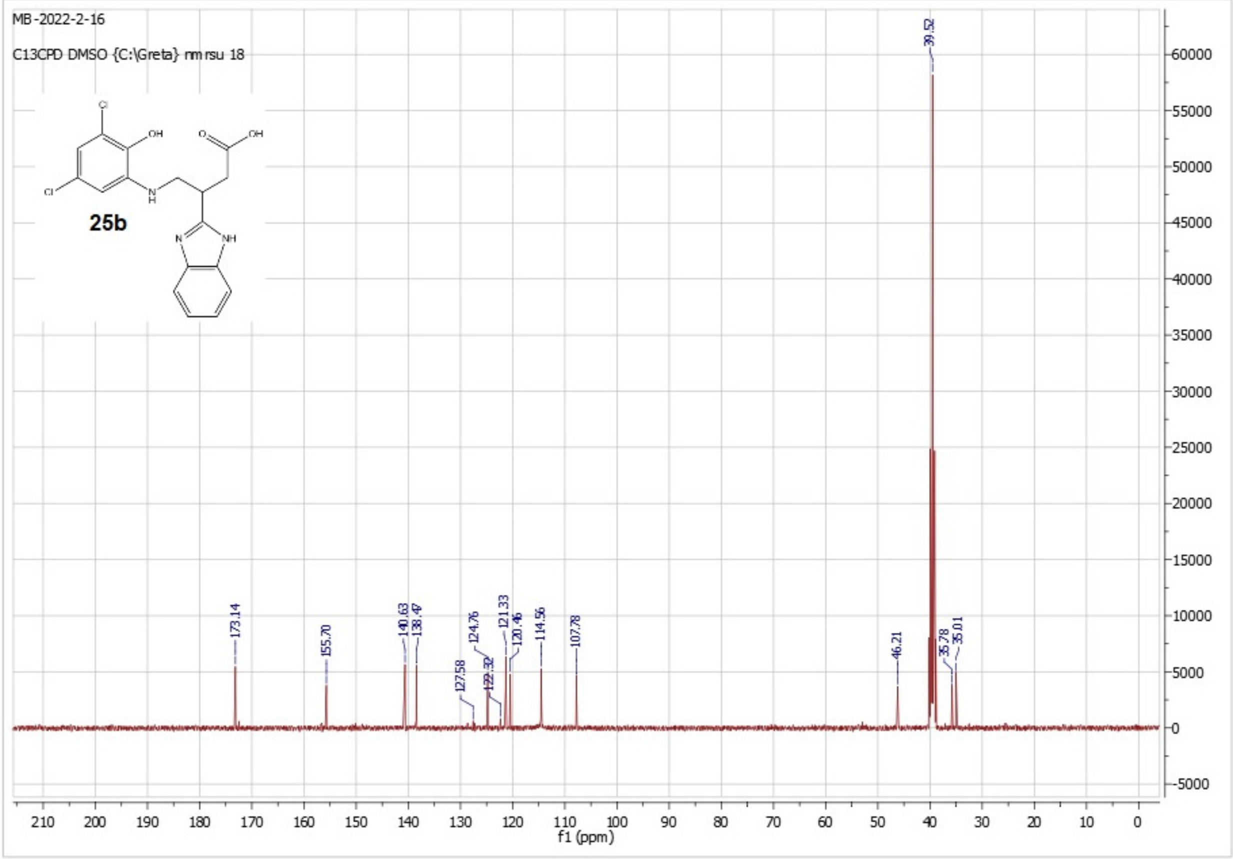

Figure S52. ^{13}C NMR spectrum of compound **25b.**

4-((2-Hydroxyphenyl)amino)-3-(5-methyl-1H-benzo[d]imidazol-2-yl)butanoic acid (26a)

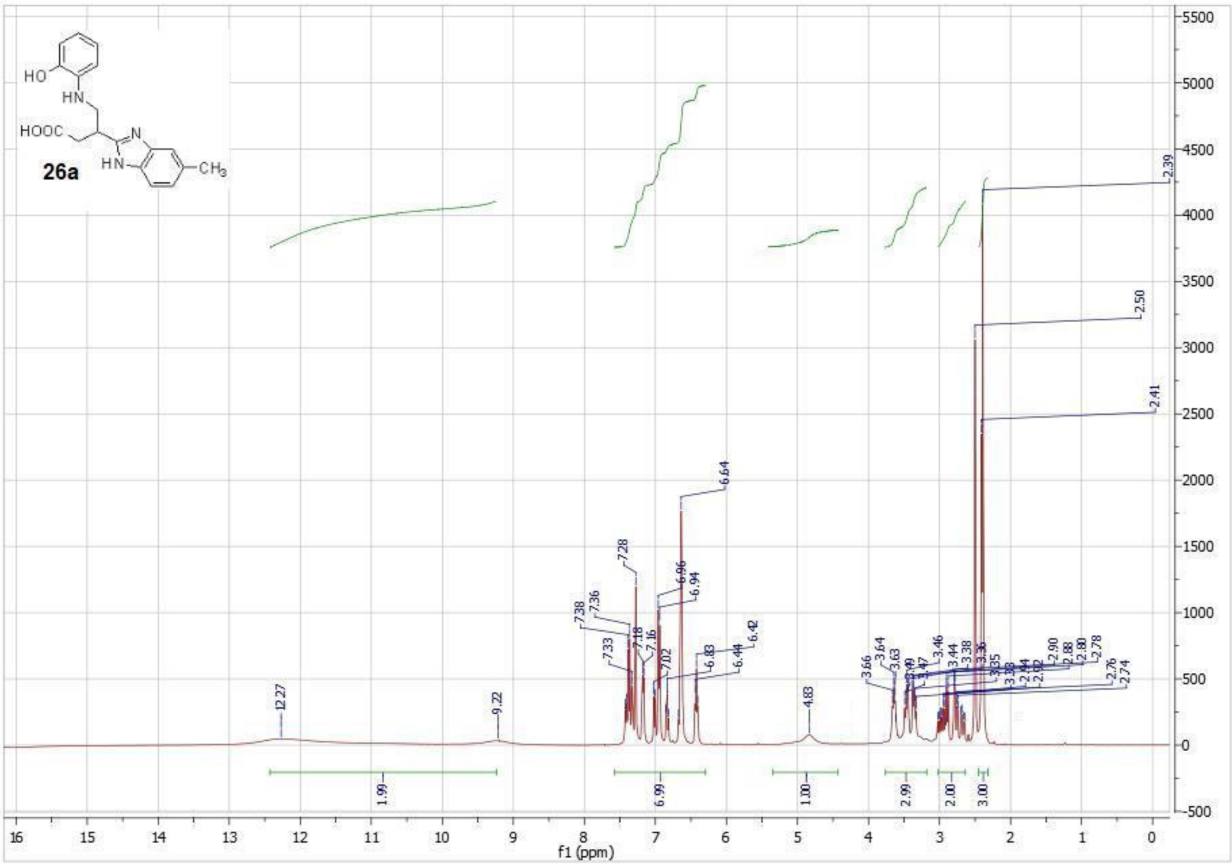

Figure S53. ^{1}H NMR spectrum of compound **26a.**

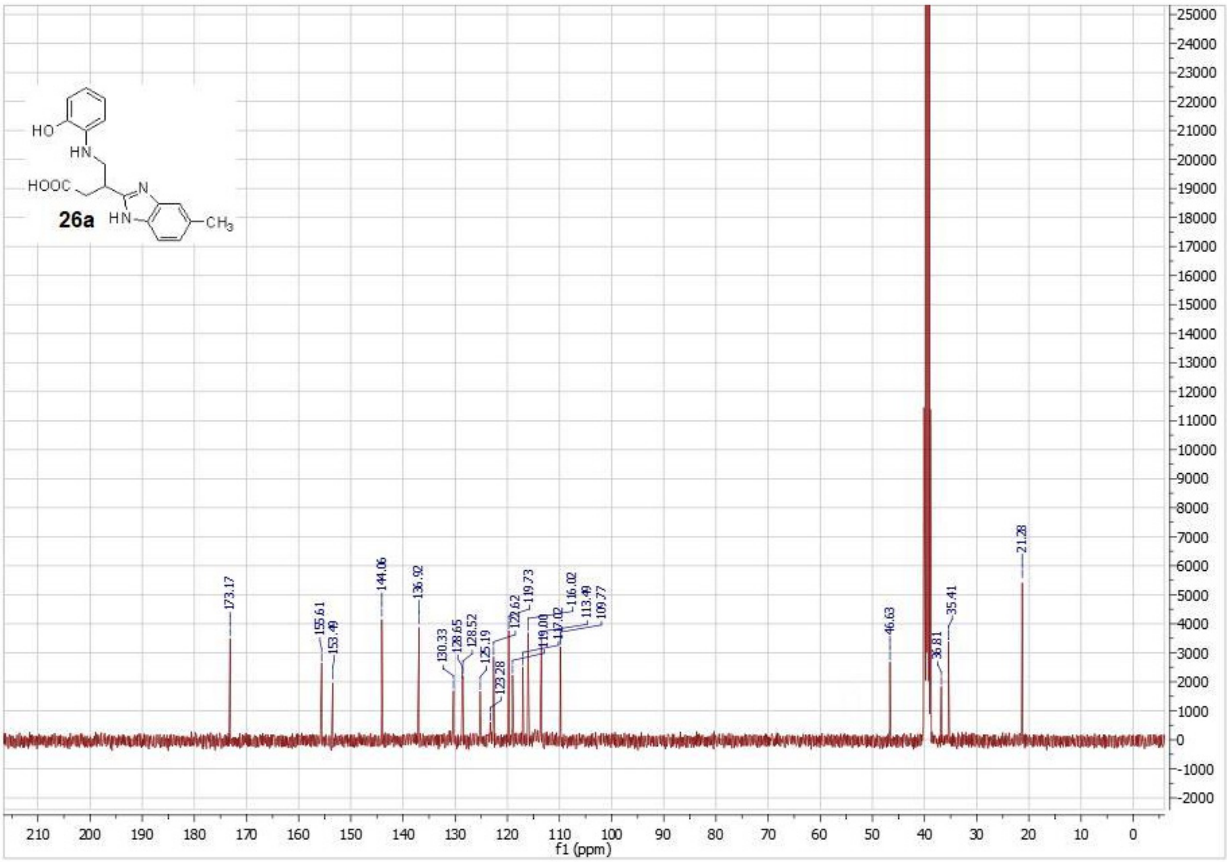

Figure S54. ^{13}C NMR spectrum of compound **26a.**

4-((3,5-Dichloro-2-hydroxyphenyl)amino)-3-(5-methyl-1H-benzo[d]imidazol-2-yl)butanoic acid (26b)

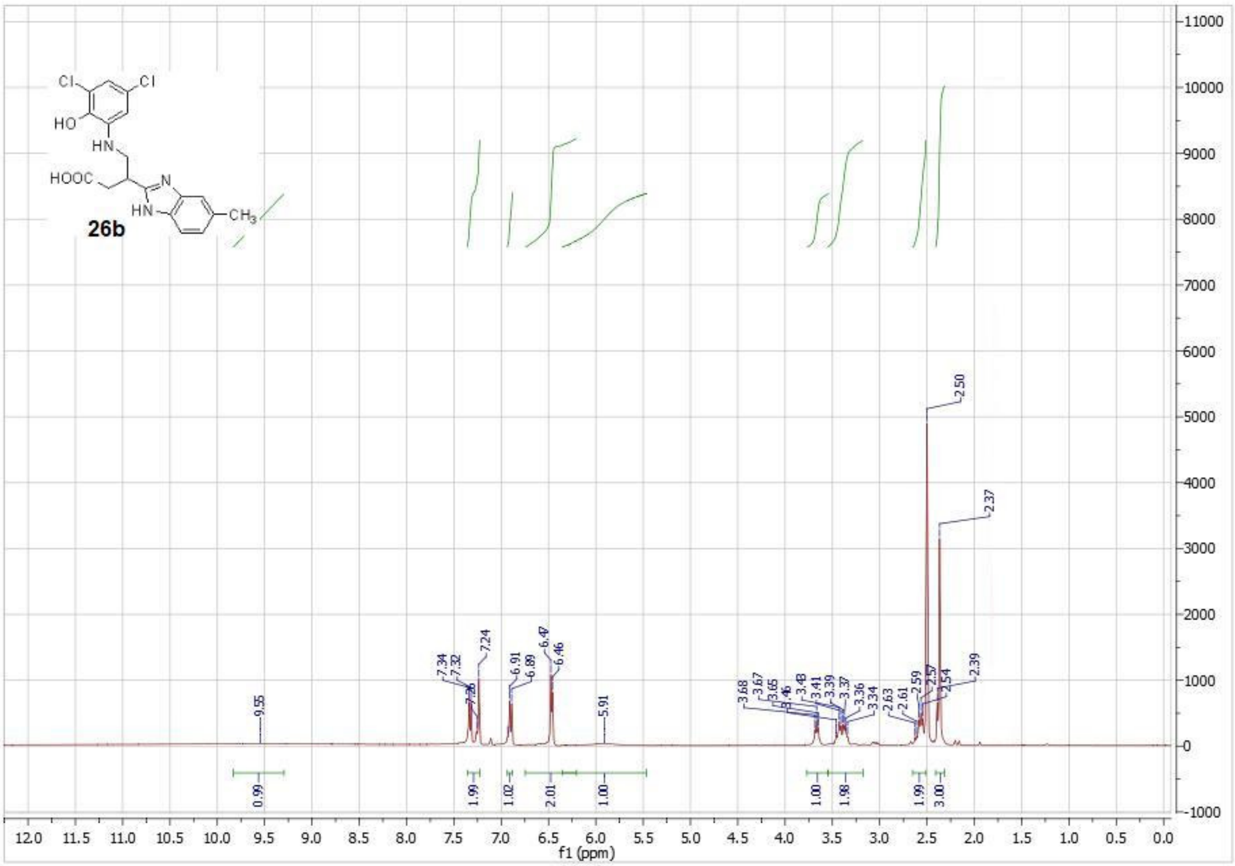

Figure S55. ^{1}H NMR spectrum of compound **26b.**

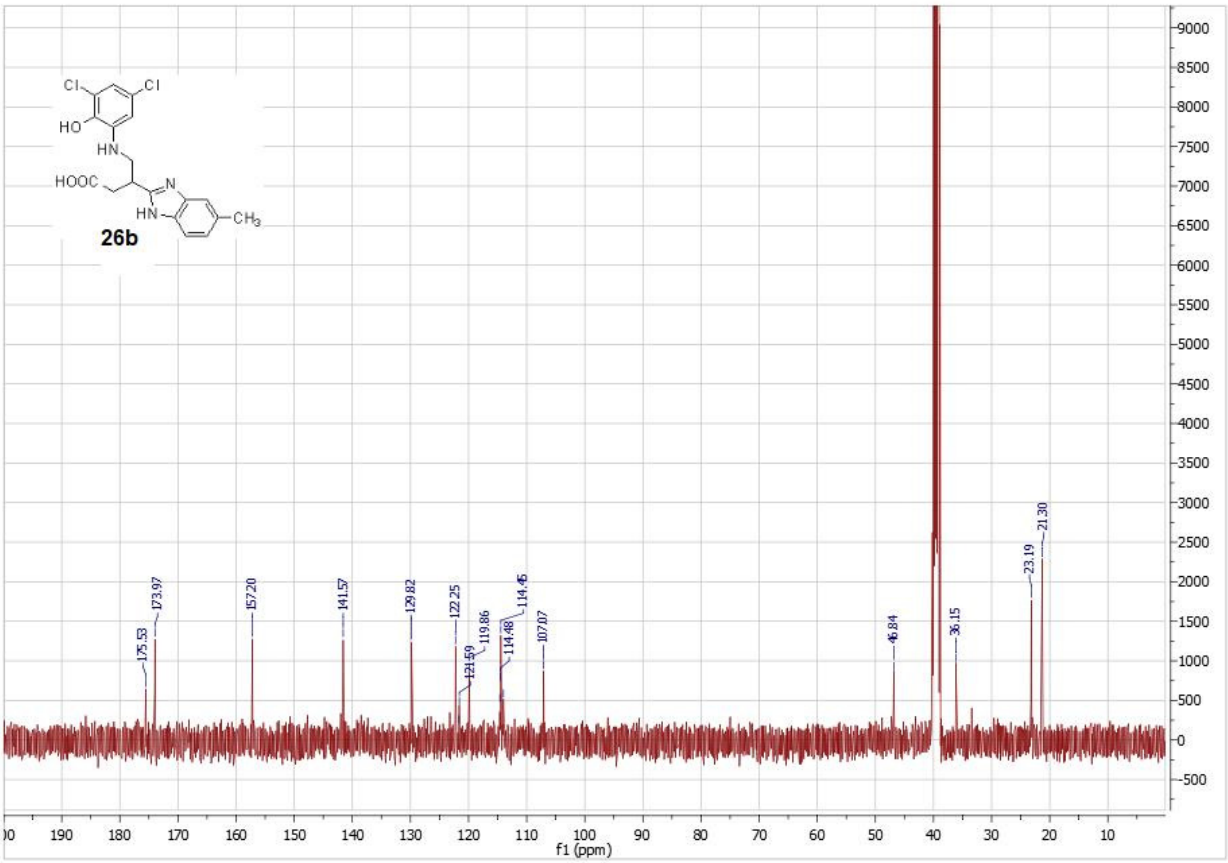

Figure S56. ^{13}C NMR spectrum of compound **26b.**

3-(5-Chloro-1H-benzo[d]imidazol-2-yl)-4-((2-hydroxyphenyl)amino)butanoic acid (27a)

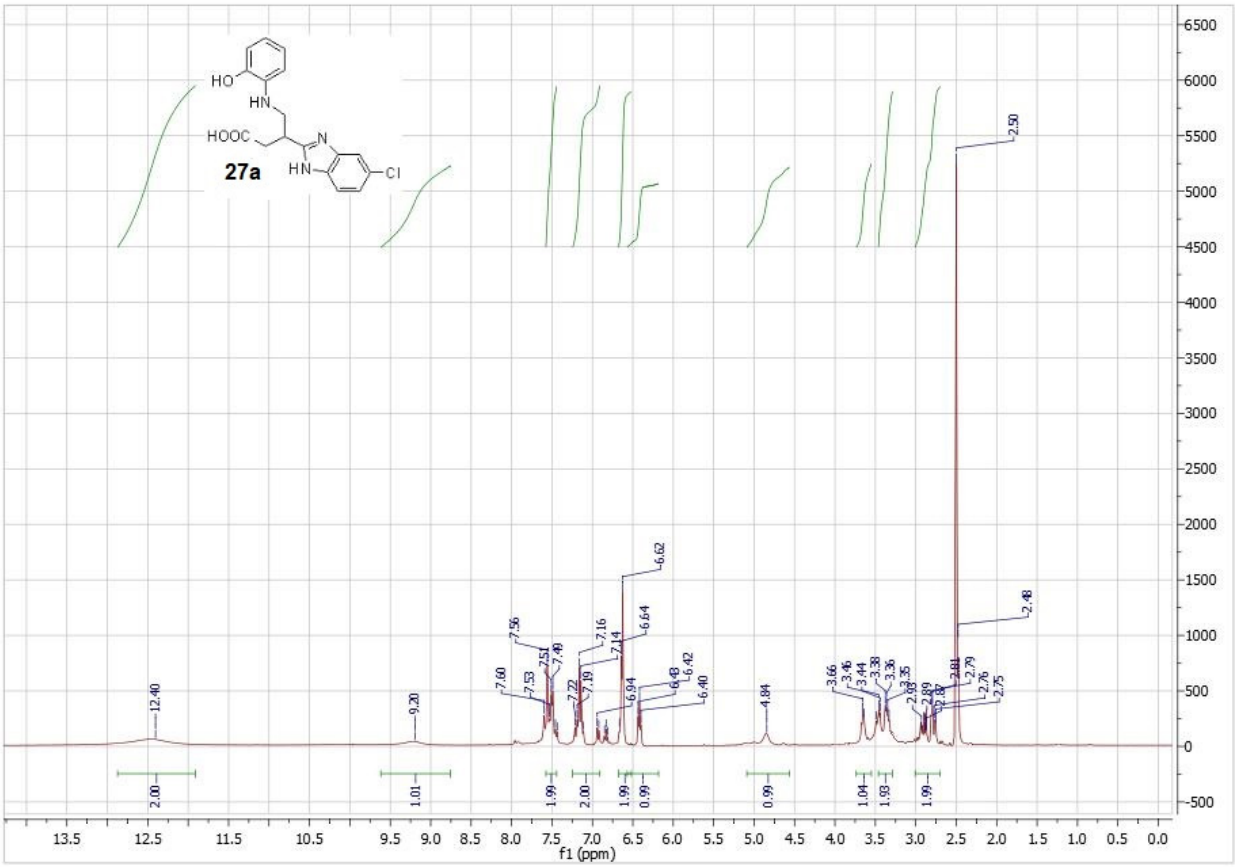

Figure S57. ^{1}H NMR spectrum of compound **27a.**

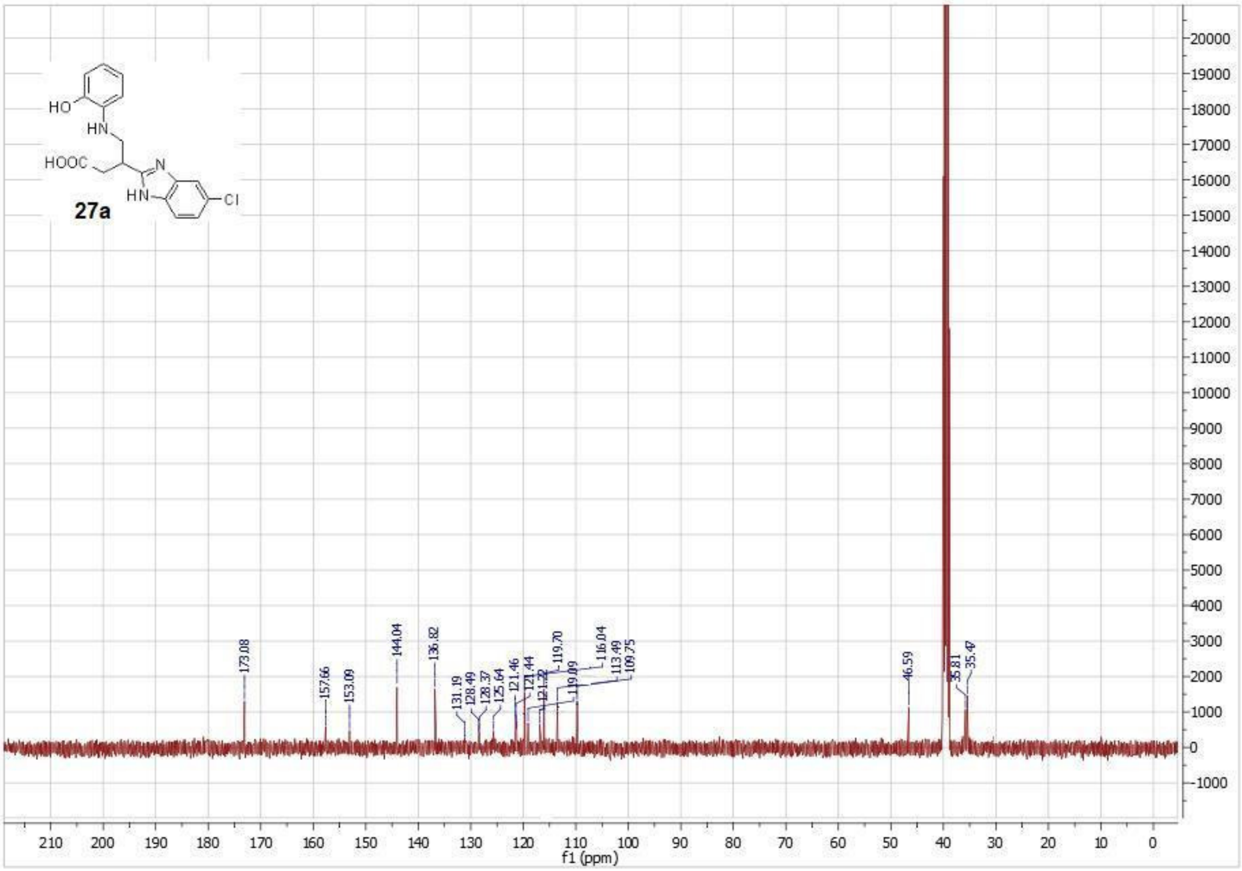

Figure S58. ^{13}C NMR spectrum of compound **27a.**

3-(5-Chloro-1H-benzo[d]imidazol-2-yl)-4-((3,5-dichloro-2-hydroxyphenyl)amino)butanoic acid (27b)

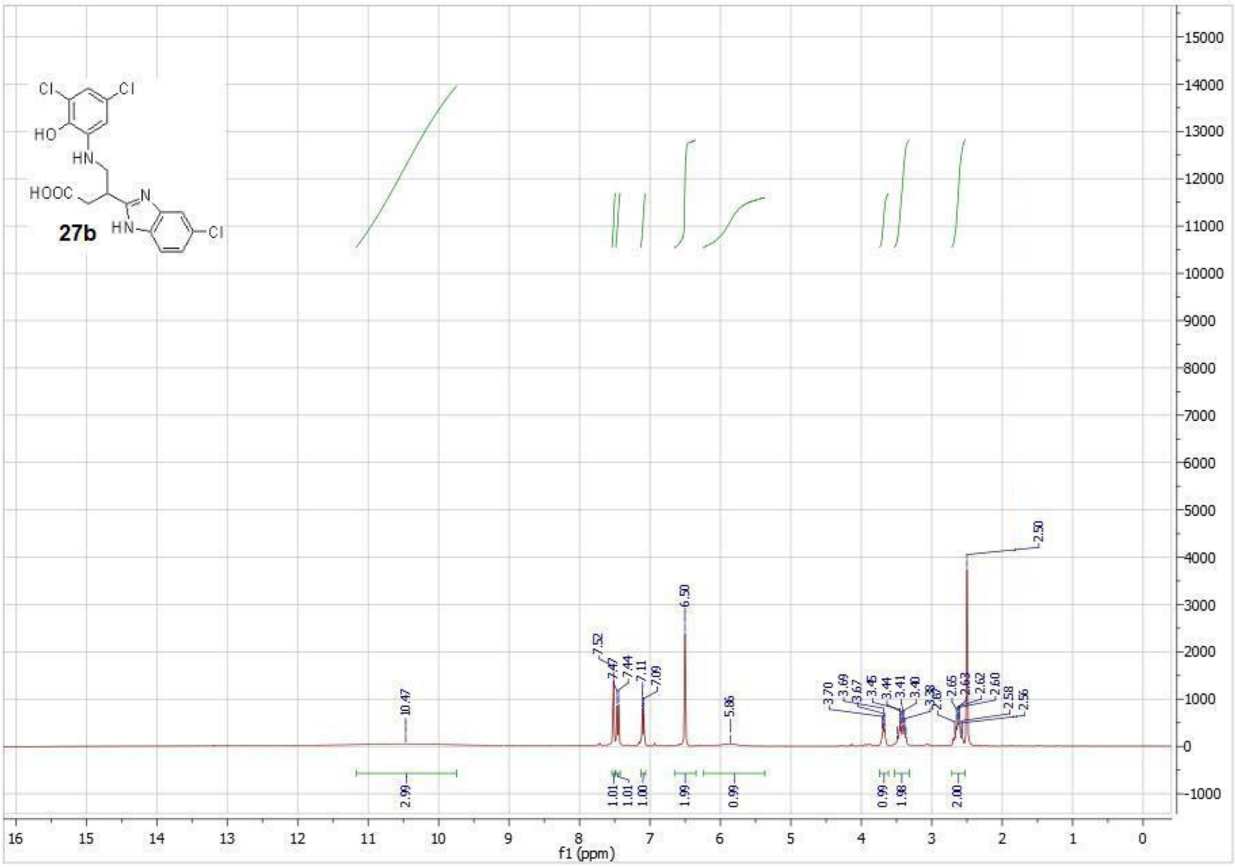

Figure S59. ^{1}H NMR spectrum of compound **27b.**

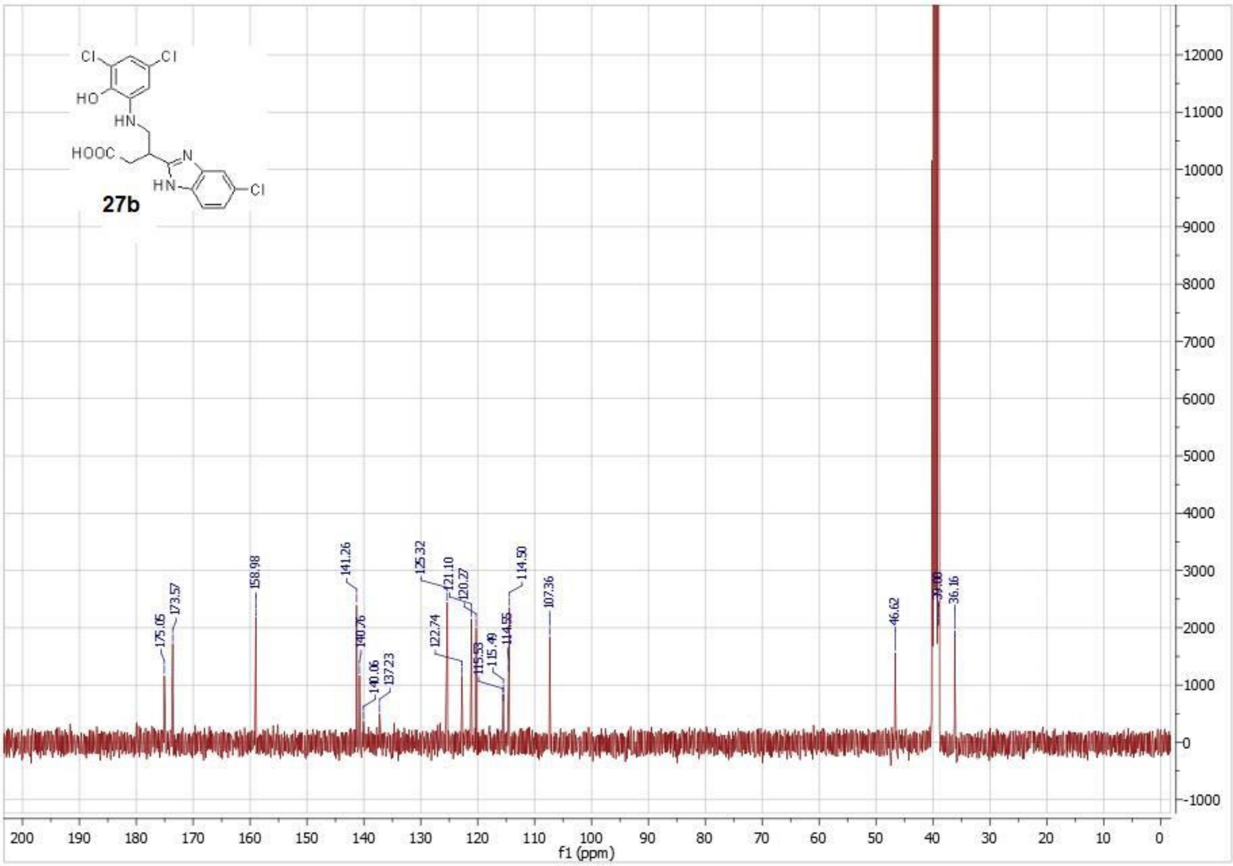

Figure S60. ^{13}C NMR spectrum of compound **27b.**

4-((3,5-Dichloro-2-hydroxyphenyl)amino)-3-(5-fluoro-1H-benzo[d]imidazol-2-yl)butanoic acid (28b)

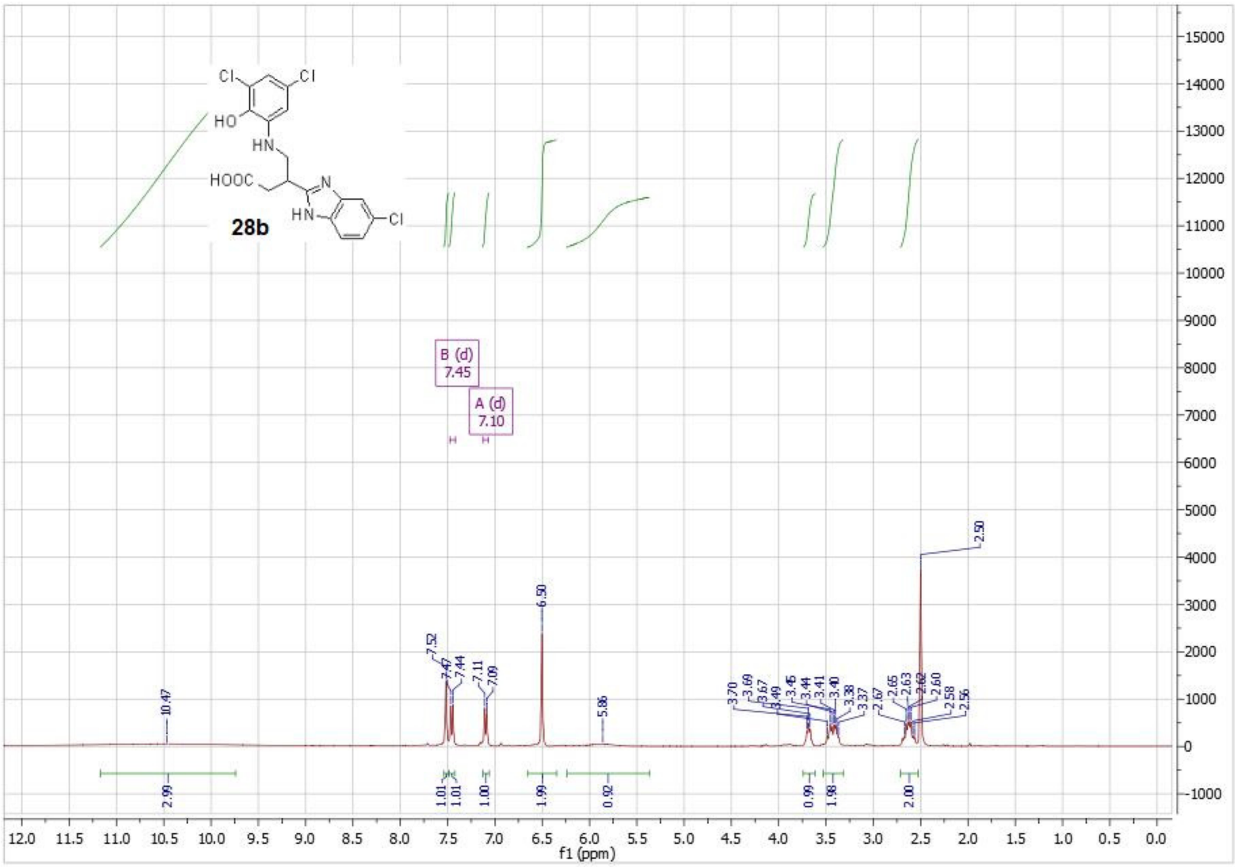

Figure S61. ^{1}H NMR spectrum of compound **28b.**

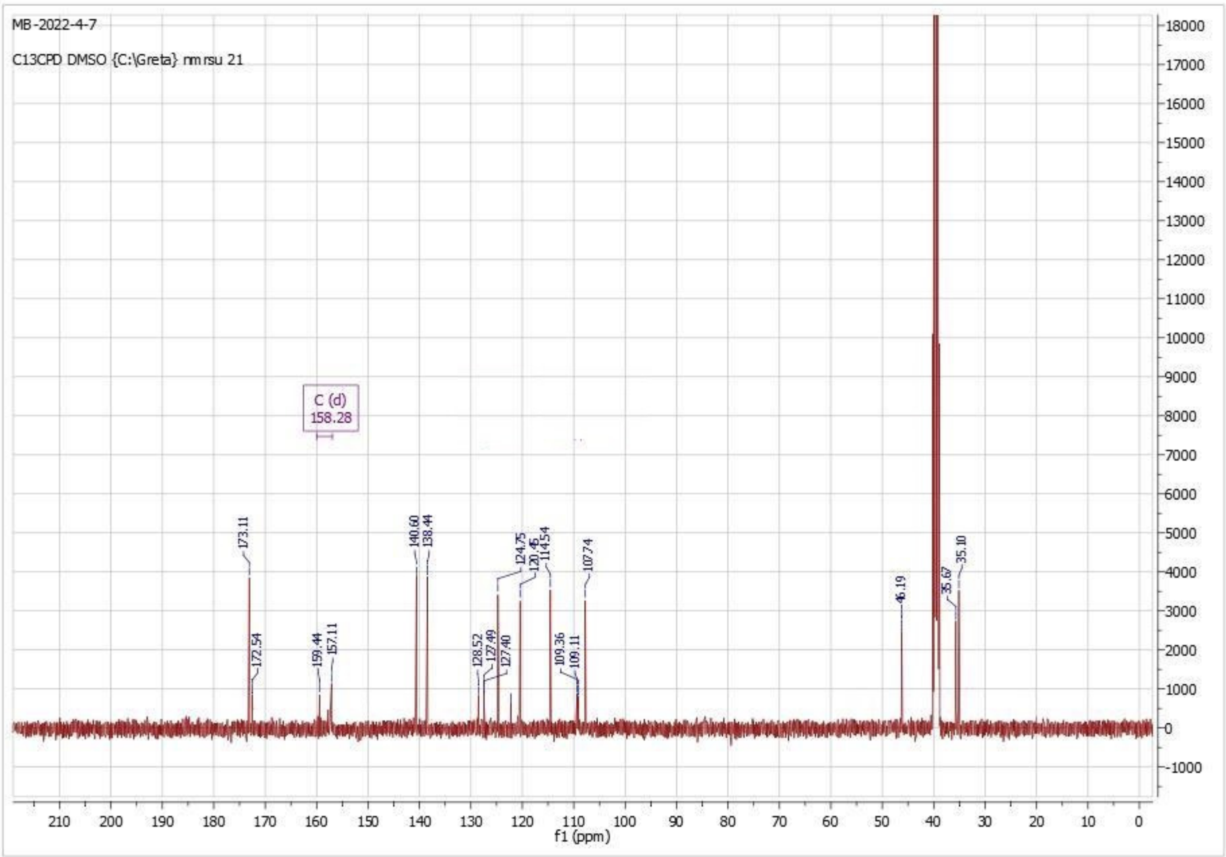

Figure S62. ^{13}C NMR spectrum of compound **28b.**

Mass Spectrum SmartFormula Report

Analysis Info

Analysis Name	D:\Data\Organikai\2022_01_19\14 Birute1_2-F,2_01_10001.d	Acquisition Date	3/2/2023 4:30:50 PM
Method	organikai_esi_pos_2013_recover.m	Operator	Milda
Sample Name	14 Birute1	Instrument / Ser#	maXis 4G 20218
Comment			

Acquisition Parameter

Source Type	ESI	Ion Polarity	Positive	Set Nebulizer	1.5 Bar
Focus	Not active	Set Capillary	4500 V	Set Dry Heater	180 °C
Scan Begin	40 m/z	Set End Plate Offset	-500 V	Set Dry Gas	8.0 l/min
Scan End	1800 m/z	Set Collision Cell RF	350.0 Vpp	Set Divert Valve	Waste

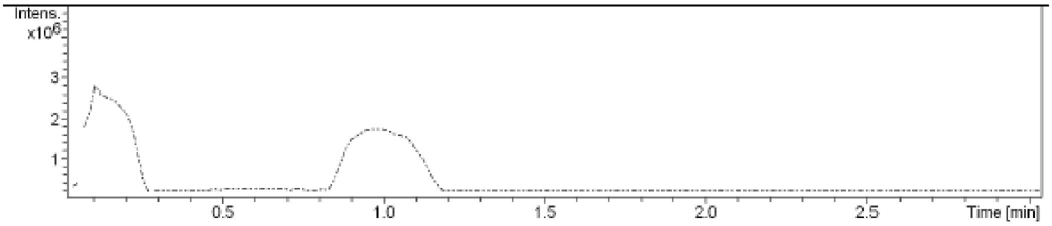

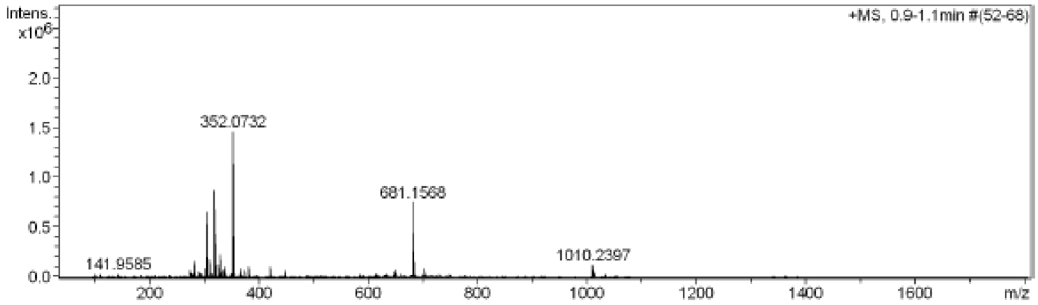

Meas. m/z	#	Formula	Score	m/z	err [ppm]	Mean err [ppm]	mSigma	rdb	e⁻ Conf	N-Rule
330.0908	1	C 14 H 14 N 6 O 2 S	53.06	330.0893	-4.3	-4.3	11.1	11.0	odd	ok
	2	C 16 H 16 N 3 O 3 S	100.00	330.0907	-0.2	-0.2	14.9	10.5	even	ok
	3	C 18 H 18 O 4 S	49.18	330.0920	3.8	3.9	19.6	10.0	odd	ok
	4	C 10 H 14 N 6 O 7	42.97	330.0918	3.3	4.2	30.3	7.0	odd	ok
	5	C 22 H 10 N 4	35.68	330.0900	-2.3	-1.1	44.1	20.0	odd	ok
	6	C 24 H 12 N O	33.09	330.0913	1.8	3.0	49.9	19.5	even	ok

Figure S63. HRMS of compound **14**.

Mass Spectrum SmartFormula Report

Analysis Info

Analysis Name	D:\Data\Organikai\2022_01_19\15 Birute2_2-F,3_01_10002.d	Acquisition Date	3/2/2023 4:35:20 PM
Method	organikai_esi_pos_2013_recover.m	Operator	Milda
Sample Name	15 Birute2	Instrument / Ser#	maXis 4G 20218
Comment			

Acquisition Parameter

Source Type	ESI	Ion Polarity	Positive	Set Nebulizer	1.5 Bar
Focus	Not active	Set Capillary	4500 V	Set Dry Heater	180 °C
Scan Begin	40 m/z	Set End Plate Offset	-500 V	Set Dry Gas	8.0 l/min
Scan End	1800 m/z	Set Collision Cell RF	350.0 Vpp	Set Divert Valve	Waste

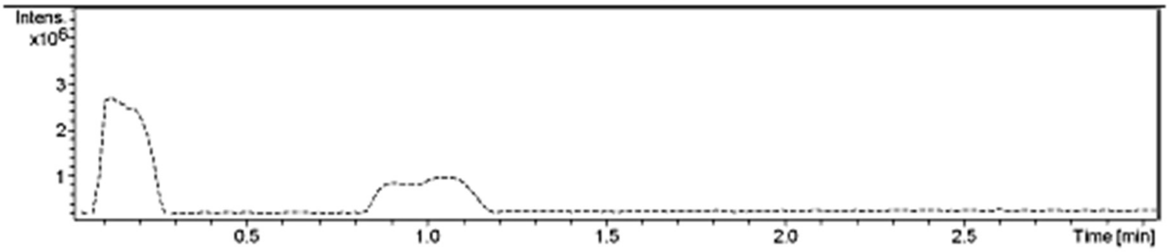

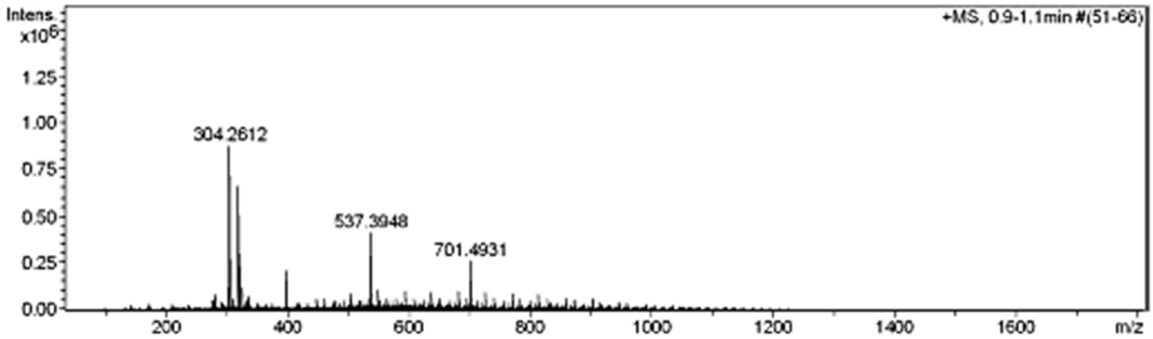

Meas. m/z	#	Formula	Score	m/z	err [ppm]	Mean err [ppm]	mSigma	rdb	e⁻ Conf	N-Rule
304.2612	1	C 15 H 34 N 3 O 3	51.54	304.2595	-5.7	-5.9	12.6	0.5	even	ok
	2	C 17 H 36 O 4	100.00	304.2608	-1.3	-1.4	18.5	0.0	odd	ok
	3	C 18 H 32 N 4	53.28	304.2621	3.1	2.9	33.1	5.0	odd	ok
318.2405	1	C 15 H 32 N 3 O 4	50.85	318.2387	-5.4	-5.2	14.2	1.5	even	ok
	2	C 17 H 34 O 5	100.00	318.2401	-1.2	-1.0	19.2	1.0	odd	ok
	3	C 16 H 28 N 7	96.60	318.2401	-1.2	-1.1	20.7	6.5	even	ok
	4	C 18 H 30 N 4 O	62.68	318.2414	3.0	3.2	26.7	6.0	odd	ok
397.0577	1	C 12 H 7 N 13 O 2 S	50.25	397.0561	-3.9	-4.1	4.8	16.0	odd	ok
	2	C 14 H 9 N 10 O 3 S	100.00	397.0574	-0.6	-0.7	7.8	15.5	even	ok
	3	C 15 H 15 N 3 O 8 S	93.79	397.0574	-0.5	-0.6	11.4	10.0	odd	ok
	4	C 13 H 13 N 6 O 7 S	43.79	397.0561	-3.9	-4.0	12.6	10.5	even	ok
	5	C 16 H 11 N 7 O 4 S	57.29	397.0588	2.8	2.7	12.9	15.0	odd	ok
	6	C 17 H 17 O 9 S	57.02	397.0588	2.8	2.8	13.0	9.5	even	ok
	7	C 21 H 9 N 4 O 5	42.19	397.0567	-2.3	-1.1	32.3	19.5	even	ok
	8	C 23 H 11 N O 6	46.82	397.0581	1.1	2.3	37.6	19.0	odd	ok
	9	C 20 H 3 N 11	32.66	397.0567	-2.3	-1.2	41.9	25.0	odd	ok
	10	C 22 H 5 N 8 O	35.82	397.0581	1.1	2.2	47.2	24.5	even	ok
	11	C 24 H 7 N 5 O 2	13.87	397.0594	4.5	5.6	52.3	24.0	odd	ok
537.3948	1	C 27 H 57 N 2 O 6 S	56.82	537.3932	-3.1	-3.5	16.9	0.5	even	ok
	2	C 26 H 51 N 9 O S	54.10	537.3932	-3.1	-3.5	19.1	6.0	odd	ok
	3	C 22 H 51 N 9 O 6	87.78	537.3957	1.6	2.2	19.5	2.0	odd	ok

Bruker Compass DataAnalysis 4.0 printed: 3/2/2023 5:12:01 PM Page 1 of 2

Figure S64. HRMS of compound **15**.

Mass Spectrum SmartFormula Report

Analysis Info			
Analysis Name	D:\Data\Organikai\2022_01_19\24 B Birute3_2-F,4_01_10003.d	Acquisition Date	3/2/2023 4:39:50 PM
Method	organikai_esi_pos_2013_recover.m	Operator	Milda
Sample Name	24 B Birute3	Instrument / Ser#	maXis 4G 20218
Comment			

Acquisition Parameter

Source Type	ESI	Ion Polarity	Positive	Set Nebulizer	1.5 Bar
Focus	Not active	Set Capillary	4500 V	Set Dry Heater	180 °C
Scan Begin	40 m/z	Set End Plate Offset	-500 V	Set Dry Gas	8.0 l/min
Scan End	1800 m/z	Set Collision Cell RF	350.0 Vpp	Set Divert Valve	Waste

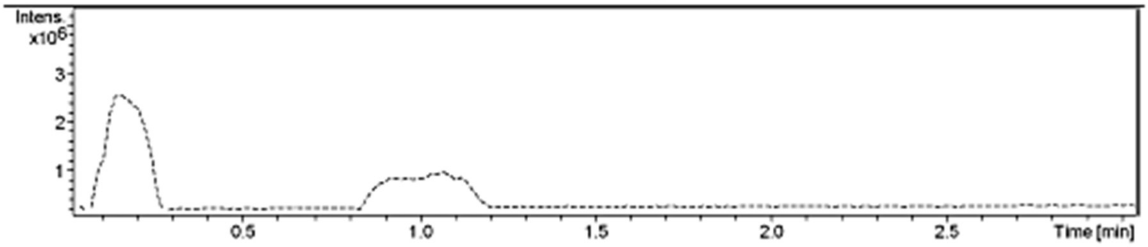

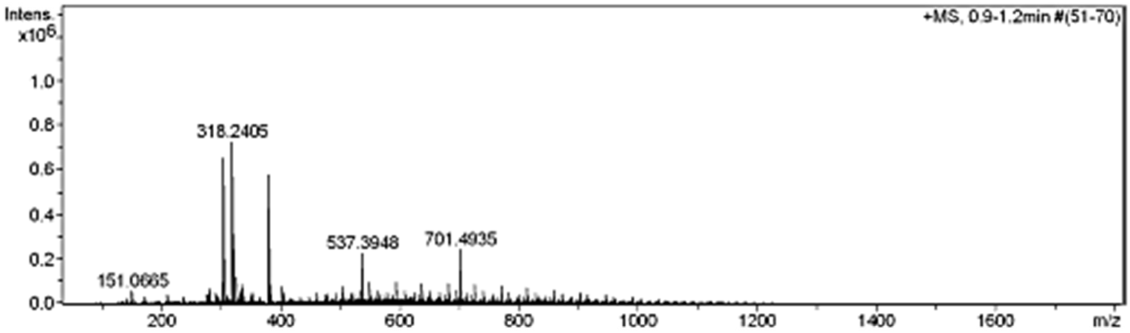

Meas. m/z	#	Formula	Score	m/z	err [ppm]	Mean err [ppm]	mSigma	rdb	e⁻ Conf	N-Rule
380.0365	1	C 11 H 2 N 13 O 4	49.94	380.0347	-4.6	-4.5	12.2	17.5	even	ok
	2	C 10 H 5 F N 10 O 6	87.42	380.0372	1.9	2.1	15.7	13.0	odd	ok
	3	C 13 H 4 N 10 O 5	100.00	380.0361	-1.1	-0.9	16.9	17.0	odd	ok
	4	C 12 H 8 N 6 O 9	44.86	380.0347	-4.6	-4.4	17.7	12.0	odd	ok
	5	C 14 H 10 N 3 O 10	94.89	380.0361	-1.1	-0.9	19.5	11.5	even	ok
	6	C 16 H 12 O 11	71.08	380.0374	2.5	2.7	20.3	11.0	odd	ok
	7	C 15 H 6 N 7 O 6	67.92	380.0374	2.5	2.6	22.6	16.5	even	ok
	8	C 11 H 11 F N 3 O 11	73.29	380.0372	1.9	2.2	23.9	7.5	even	ok
	9	C 14 N 14 O	62.74	380.0374	2.4	2.6	26.2	22.0	odd	ok
	10	C 9 H 9 F N 6 O 10	72.55	380.0359	-1.6	-1.4	27.2	8.0	odd	ok
	11	C 15 H 11 Cl 2 F N 6 O	35.89	380.0350	-3.9	-3.7	35.2	12.0	odd	ok
	12	C 9 H 12 Cl 2 N 9 O 4	26.15	380.0384	5.0	5.1	35.9	7.5	even	ok
	13	C 17 H 13 Cl 2 F N 3 O 2	65.09	380.0363	-0.4	-0.2	39.9	11.5	even	ok
	14	C 10 H 18 Cl 2 N 2 O 9	21.56	380.0384	5.0	5.2	42.9	2.0	odd	ok
	15	C 19 H 15 Cl 2 F O 3	33.57	380.0377	3.2	3.4	44.1	11.0	odd	ok
	16	C 20 H 12 Cl 2 N 3 O	27.19	380.0352	-3.4	-3.2	49.6	15.5	even	ok
	17	C 22 H 14 Cl 2 O 2	42.31	380.0365	0.2	0.4	55.5	15.0	odd	ok
	18	C 23 H 7 F N O 4	23.88	380.0354	-2.9	-2.7	57.0	20.5	even	ok
	19	C 24 H 3 F N 5	23.46	380.0367	0.6	0.8	70.5	25.5	even	ok
	20	C 26 H 5 F N 2 O	8.76	380.0380	4.1	4.3	76.8	25.0	odd	ok
	21	C 29 H 4 N 2	12.83	380.0369	1.1	1.3	83.5	29.0	odd	ok

Figure S65. HRMS of compound **24b**.

Mass Spectrum SmartFormula Report

Analysis Info

Analysis Name	D:\Data\Organikai\2022_01_19\28 B Birute4_2-F,5_01_10004.d	Acquisition Date	3/2/2023 4:44:22 PM
Method	organikai_esi_pos_2013_recover.m	Operator	Milda
Sample Name	28 B Birute4	Instrument / Ser#	maXis 4G 20218
Comment			

Acquisition Parameter

Source Type	ESI	Ion Polarity	Positive	Set Nebulizer	1.5 Bar
Focus	Not active	Set Capillary	4500 V	Set Dry Heater	180 °C
Scan Begin	40 m/z	Set End Plate Offset	-500 V	Set Dry Gas	8.0 l/min
Scan End	1800 m/z	Set Collision Cell RF	350.0 Vpp	Set Divert Valve	Waste

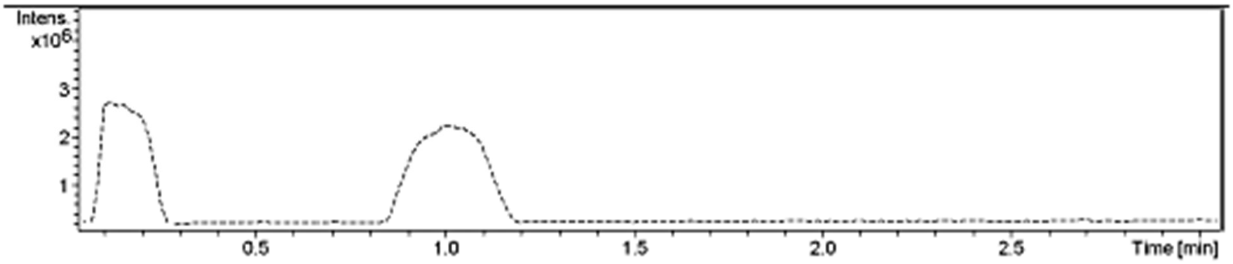

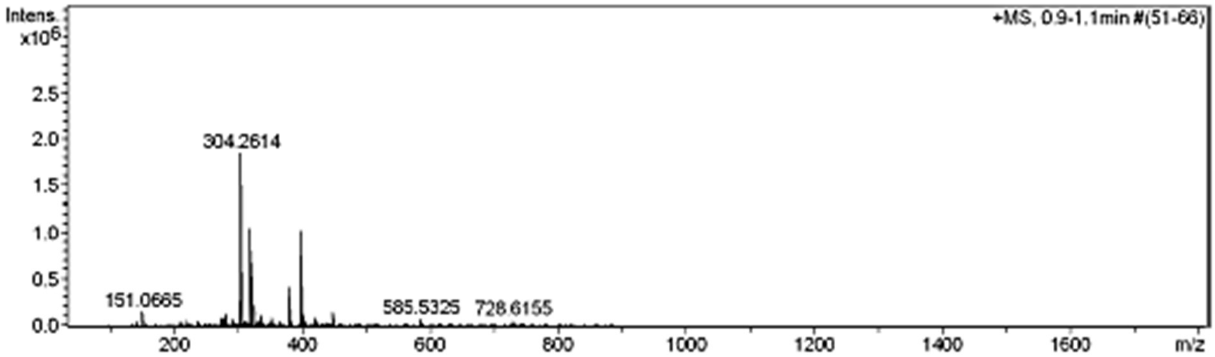

Meas. m/z	#	Formula	Score	m/z	err [ppm]	Mean err [ppm]	mSigma	rdb	e⁻ Conf	N-Rule
398.0472	1	C 13 H 15 F O 13	44.31	398.0491	4.9	5.1	18.9	6.0	odd	ok
	2	C 14 H 12 N 3 O 11	100.00	398.0466	-1.3	-1.1	21.7	10.5	even	ok
	3	C 11 H 13 F N 3 O 12	95.23	398.0478	1.5	1.7	22.3	6.5	even	ok
	4	C 16 H 14 O 12	84.60	398.0480	2.0	2.3	23.0	10.0	odd	ok
	5	C 10 H 20 Cl 2 N 2 O 10	23.92	398.0490	4.5	4.9	48.1	1.0	odd	ok
	6	C 17 H 15 Cl 2 F N 3 O 3	56.16	398.0469	-0.7	-0.3	48.8	10.5	even	ok
	7	C 19 H 17 Cl 2 F O 4	32.68	398.0482	2.7	3.1	52.9	10.0	odd	ok
	8	C 20 H 14 Cl 2 N 3 O 2	21.82	398.0458	-3.5	-3.1	59.0	14.5	even	ok
	9	C 23 H 9 F N O 5	22.53	398.0459	-3.1	-2.9	61.2	19.5	even	ok
	10	C 22 H 16 Cl 2 O 3	37.25	398.0471	-0.2	0.3	64.0	14.0	odd	ok
	11	C 26 H 7 F N 2 O 2	9.31	398.0486	3.6	3.8	81.5	24.0	odd	ok
	12	C 29 H 6 N 2 O	13.85	398.0475	0.7	1.0	86.9	28.0	odd	ok
	13	C 13 H 17 Cl N O 11	5.21	398.0485	3.3	3.5	96.8	5.5	even	ok
	14	C 10 H 19 Cl O 14	4.47	398.0458	-3.5	-3.2	98.9	1.0	odd	ok
	15	C 20 H 12 Cl F N 2 O 4	5.99	398.0464	-1.9	-1.6	100.5	15.0	odd	ok
	16	C 23 H 11 Cl N 2 O 3	2.53	398.0453	-4.8	-4.5	103.3	19.0	odd	ok
	17	C 23 H 10 Cl F N 3 O	2.16	398.0491	4.8	5.1	106.1	19.5	even	ok
	18	C 26 H 9 Cl N 3	3.69	398.0480	2.0	2.3	110.0	23.5	even	ok

Figure S66. HRMS of compound **28b**.

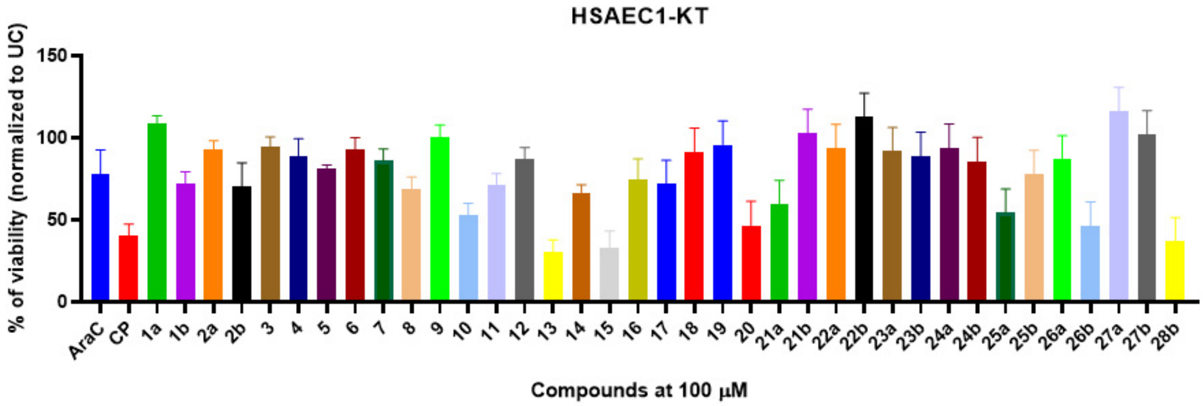

Figure S67. The viability of HSAEC1-KT primary-like pulmonary cells after 24 h treatment with compounds 1a-28b and reference drugs: cisplatin (CP) and cytosine arabinoside (AraC) with fixed concentration of 100 µM. The post-treatment viability was evaluated by MTT assay and % of viability was calculated from untreated control. Data shown are mean ± SD values from 3 separate experiments for each group.
